# Supplementary material for: High-performance blue OLED using multiresonance thermally activated delayed fluorescence host materials containing silicon atoms
Source: Nat Commun. 2023 Sep 11;14:5589. doi: 10.1038/s41467-023-41440-1 (PMC10495399; doi:10.1038/s41467-023-41440-1)
Supplement: Supplementary file 1 — Supplementary Information [file 41467_2023_41440_MOESM1_ESM.pdf]

Supplementary Information

High-performance blue OLED using multiresonance thermally activated delayed fluorescence host materials containing silicon atoms

Dongmin Park[1,¥], Seokwoo Kang[2,¥], Chi Hyun Ryoo[1], Byung Hak Jhun[1], Seyoung Jung[1], Thi Na Le[3], Min Chul Suh[3], Jaehyun Lee[4], Mi Eun Jun[5], Changwoong Chu[5], Jongwook Park[2*], Soo Young Park[1*]

[1]Center for Supramolecular Optoelectronic Materials (CSOM), Department of Materials Science and Engineering, Seoul National University, 1 Gwanak-ro, Gwanak-gu, Seoul, 08826, Republic of Korea. [2]Integrated Engineering, Department of Chemical Engineering, Kyung Hee University, Gyeonggi 17104, Republic of Korea. [3]Department of Information Display, Kyung Hee University, Dongdaemoon-Gu, Seoul, 02447, Republic of Korea. [4]Advanced Chemical Materials R&D Team, Korea Testing & Research Institute, Gwangyang, 57765, Republic of Korea. [5]Samsung Display, 1 Samsung-ro Giheung-Gu, Yongin, 17113, Republic of Korea. [¥]These authors contributed equally: Dongmin Park, Seokwoo Kang *e-mail: jongpark@khu.ac.kr and parksy@snu.ac.kr

Table of Contents

Supplementary	Contents	Page
**Supplementary Discussion**		
**Fig. S1**	**Single crystal XRD analysis**. **a** TDBA-Ph. **b** TDBA-Si.	**S7**
**Fig. S2**	**Optimized (B3LYP/6-31g(d,p)) geometries at the ground state and dihedral angle between TDBA core and peripheral side groups (phenyl and tetraphenylsilane). a** TDBA. **b** TDBA-Ph. **c** mTDBA-Ph. **d** mTDBA-2Ph. **e** TDBA-Si. **f** mTDBA-Si. **g** mTDBA-2Si.	**S8**
**Fig. S3**	Isosurface of HOMO and LUMO composing $S_0 \rightarrow S_1$ transition (isovalue = 0.02) with representative electronic transition energies with SOC values of TDBA. TD-B3LYP calculation was conducted at the level of 6-31G(d,p).	**S9**
**Fig. S4**	Isosurface of HOMO and LUMO composing $S_0 \rightarrow S_1$ transition (isovalue = 0.02) with representative electronic transition energies with SOC values of TDBA-Ph. TD-B3LYP calculation was conducted at the level of 6-31G(d,p).	**S9**
**Fig. S5**	Isosurface of HOMO and LUMO composing $S_0 \rightarrow S_1$ transition (isovalue = 0.02) with representative electronic transition energies with SOC values of mTDBA-Ph. TD-B3LYP calculation was conducted at the level of 6-31G(d,p).	**S10**
**Fig. S6**	Isosurface of HOMO and LUMO composing $S_0 \rightarrow S_1$ transition (isovalue = 0.02) with representative electronic transition energies with SOC values of mTDBA-2Ph. TD-B3LYP calculation was conducted at the level of 6-31G(d,p).	**S10**
**Fig. S7**	Isosurface of HOMO and LUMO composing $S_0 \rightarrow S_1$ transition (isovalue = 0.02) with representative electronic transition energies with SOC values of TDBA-Si. TD-B3LYP calculation was conducted at the level of 6-31G(d,p).	**S11**
**Fig. S8**	Isosurface of HOMO and LUMO composing $S_0 \rightarrow S_1$ transition (isovalue = 0.02) with representative electronic transition energies with SOC values of mTDBA-Si. TD-B3LYP calculation was conducted at the level of 6-31G(d,p).	**S11**
**Fig. S9**	Isosurface of HOMO and LUMO composing $S_0 \rightarrow S_1$ transition (isovalue = 0.02) with representative electronic transition energies with SOC values of mTDBA-Si. TD-B3LYP calculation was conducted at the level of 6-31G(d,p).	**S12**
**Fig. S10**	Calculated electronic transitions and each transition character of TDBA.	**S13**
**Fig. S11**	Calculated electronic transitions and each transition character of TDBA-Ph.	**S14**
**Fig. S12**	Calculated electronic transitions and each transition character of mTDBA-Ph.	**S15**
**Fig. S13**	Calculated electronic transitions and each transition character of mTDBA-2Ph.	**S16**
**Fig. S14**	Calculated electronic transitions and each transition character of TDBA-Si.	**S17**
**Fig. S15**	Calculated electronic transitions and each transition character of mTDBA-Si.	**S18**
**Fig. S16**	Calculated electronic transitions and each transition character of mTDBA-2Si.	**S19**
**Fig. S17**	**Single-crystal XRD structures with Oak Ridge Thermal Ellipsoid Plot (ORTEP) drawing of the selected materials with 50% probability level**. **a** TDBA-Ph. **b** TDBA-Si **c** mTDBA-Ph. **d** mTDBA-Si.	**S20**

**Fig. S18**	**Photoelectron spectroscopy (AC-2) of TDBA-based host materials. a** TDBA. **b** TDBA-Ph. **c** mTDBA-Ph. **d** mTDBA-2Ph. **e** TDBA-Si. **f** mTDBA-Si. **g** mTDBA-2Si.	**S21**
**Fig. S19**	**Absorption spectra(dash) at room temperature, low temeperature photoluminescence without (line) and with delay (dot) at 77K spectra of neat film**. **a** TDBA-Ph. **b** mTDBA-Ph. **c** mTDBA-2Ph. **d** TDBA-Si. **e** mTDBA-Si. **f** mTDBA-2Si.	**S22**
**Fig. S20**	**UV-Visible absorption and excitation spectra of 2% ν-DABNA films using TDBA-based host materials. a** TDBA-Ph. **b** mTDBA-Ph. **c** mTDBA-2Ph. **d** TDBA-Si. **e** mTDBA-Si. **f** mTDBA-2Si.	**S23**
**Fig. S21**	**Spectral overlap between ν-DABNA and TDBA-based host. a** TDBA-Ph. **b** mTDBA-Ph. **c** mTDBA-2Ph. **d** TDBA-Si. **e** mTDBA-Si. **f** mTDBA-2Si.	**S24**
**Fig. S22**	**Molecule orientation in doping films according to TDBA-host materials. a** TDBA-Ph. **b** mTDBA-Ph. **c** mTDBA-2Ph. **d** TDBA-Si. **e** mTDBA-Si. **f** mTDBA-2Si.	**S25**
**Fig. S23**	**Optical microscopy image of the 2 wt% ν-DABNA-doped TDBA-based film. a** TDBA-Ph. **b** mTDBA-Ph. **c** mTDBA-2Ph. **d** TDBA-Si. **e** mTDBA-Si. **f** mTDBA-2Si.	**S26**
**Fig. S24**	**AFM image of the 2 wt% ν-DABNA-doped TDBA-based film**. **a** TDBA-Ph. **b** mTDBA-Ph. **c** mTDBA-2Ph. **d** TDBA-Si. **e** mTDBA-Si. **f** mTDBA-2Si.	**S26**
**Fig. S25**	Thermo gravimetric analyzer (TGA) results of TDBA-based host materials.	**S27**
**Fig. S26**	**Differential scanning calorimetry (DSC) results of TDBA-based host materials**. **a** TDBA-Ph. **b** mTDBA-Ph. **c** mTDBA-2Ph. **d** TDBA-Si. **e** mTDBA-Si. **f** mTDBA-2Si.	**S27**
**Fig. S27**	Synthetic route of TDBA-based host materials.	**S28**
**Fig. S28**	^{1}H-NMR spectra of **4,4'-((2-bromo-1,3-phenylene)bis(oxy))bis(*tert*-butylbenzene).**	**S29**
**Fig. S29**	^{13}C-NMR spectra of **4,4'-((2-bromo-1,3-phenylene)bis(oxy))bis(*tert*-butylbenzene).**	**S29**
**Fig. S30**	^{1}H-NMR spectra of **TDBA.**	**S30**
**Fig. S31**	^{13}C-NMR spectra of **TDBA.**	**S30**
**Fig. S32**	^{1}H-NMR spectra of **mTDBA-Br.**	**S31**
**Fig. S33**	^{13}C-NMR spectra of **mTDBA-Br.**	**S31**
**Fig. S34**	^{1}H-NMR spectra of **mTDBA-2Br.**	**S32**
**Fig. S35**	^{13}C-NMR spectra of **mTDBA-2Br.**	**S32**
**Fig. S36**	^{1}H-NMR spectra of **TDBA-Ph.**	**S33**
**Fig. S37**	^{13}C-NMR spectra of **TDBA-Ph.**	**S33**
**Fig. S38**	^{1}H-NMR spectra of **mTDBA-Ph.**	**S34**
**Fig. S39**	^{13}C-NMR spectra of **mTDBA-Ph**.	**S34**
**Fig. S40**	^{1}H-NMR spectra of **mTDBA-2Ph.**	**S35**
**Fig. S41**	^{13}C-NMR spectra of **mTDBA-2Ph.**	**S35**
**Fig. S42**	^{1}H-NMR spectra of **TDBA-Si.**	**S36**

**Fig. S43**	^{13}C-NMR spectra of **TDBA-Si.**	**S36**
**Fig. S44**	^{1}H-NMR spectra of **mTDBA-Si.**	**S37**
**Fig. S45**	^{13}C-NMR spectra of **mTDBA-Si.**	**S37**
**Fig. S46**	^{1}H-NMR spectra of **mTDBA-2Si.**	**S38**
**Fig. S47**	^{13}C-NMR spectra of **mTDBA-2Si.**	**S38**
**Table S1**	Summary of TD-DFT calculation results of TDBA-based material calculated at the B3LYP/6-31G(d,p).	**S39**
**Table S2**	Summary of Energy state TD-DFT calculation results of TDBA-based material calculated at the B3LYP/6-31G(d,p).	**S40**
**Table S3**	Summary of SOC values and representative electronic transition energies of TDBA-based materials.	**S41**
**Table S4**	Rate constant for TDBA based host materials (non-doped film) at room temperature.	**S42**
**Table S5**	Fitting the decay curves triexponentially according to host materials in doped films.	**S43**
**Table S6**	Rate constant for ν-DABNA in TDBA based host materials (2wt% doped) at room temperature.	**S44**
**Table S7**	Rate constant of energy transfer between host and dopant based on Stern-Volmer equation.	**S45**
**Table S8**	FRET calculation summary between newly synthesized host materials and ν-DABNA.	**S46**
**Table S9**	Charge mobility of TDBA-based host materials.	**S47**
**Table S10**	Summary of the reported multiple resonance (MR) and donor-acceptor (DA) type OLEDs (CIE y < 0.15)	**S48**
**Supplemtetary Methods**		
**4,4'-((2-bromo-1,3-phenylene)bis(oxy))bis(*tert*-butylbenzene) (1).**		**S49**
**2,12-di-*tert*-butyl-5,9-dioxa-13b-boranaphtho[3,2,1-*de*]anthracene (TDBA).**		**S49**
**6-bromo-2,12-di-*tert*-butyl-5,9-dioxa-13b-boranaphtho[3,2,1-*de*]anthracene (mTDBA-Br).**		**S50**
**6,8-dibromo-2,12-di-*tert*-butyl-5,9-dioxa-13b-boranaphtho[3,2,1-*de*]anthracene (mTDBA-2Br).**		**S50**
**4,4'-((2,5-dibromo-1,3-phenylene)bis(oxy))bis(tert-butylbenzene) (2)**		**S50**
**7-bromo-2,12-di-*tert*-butyl-5,9-dioxa-13b-boranaphtho[3,2,1-*de*]anthracene (TDBA-Br).**		**S51**
**2,12-di-*tert*-butyl-7-phenyl-5,9-dioxa-13b-boranaphtho[3,2,1-*de*]anthracene (TDBA-Ph).**		**S51**
**2,12-di-*tert*-butyl-6-phenyl-5,9-dioxa-13b-boranaphtho[3,2,1-*de*]anthracene (mTDBA-Ph).**		**S51**
**2,12-di-*tert*-butyl-6,8-diphenyl-5,9-dioxa-13b-boranaphtho[3,2,1-*de*]anthracene (mTDBA-2Ph).**		**S51**
**4-bromo-triphenylsilylbenzene (3).**		**S51**

**4-triphenylsilylphenylboronic acid (4).**	**S52**
**(4-(2,12-di-*tert*-butyl-5,9-dioxa-13b-boranaphtho[3,2,1-*de*]anthracen-7-yl)phenyl) triphenylsilane (TDBA-Si).**	**S52**
**(4-(2,12-di-*tert*-butyl-5,9-dioxa-13b-boranaphtho[3,2,1-*de*]anthracen-6-yl)phenyl) triphenylsilane (mTDBA-Si).**	**S52**
**((2,12-di-*tert*-butyl-5,9-dioxa-13b-boranaphtho[3,2,1-*de*]anthracene-6,8-diyl)bis(4,1-phenylene))bis(triphenylsilane) (mTDBA-2Si).**	**S52**
**Supplementary References**	**S53**

Supplementary Discussion

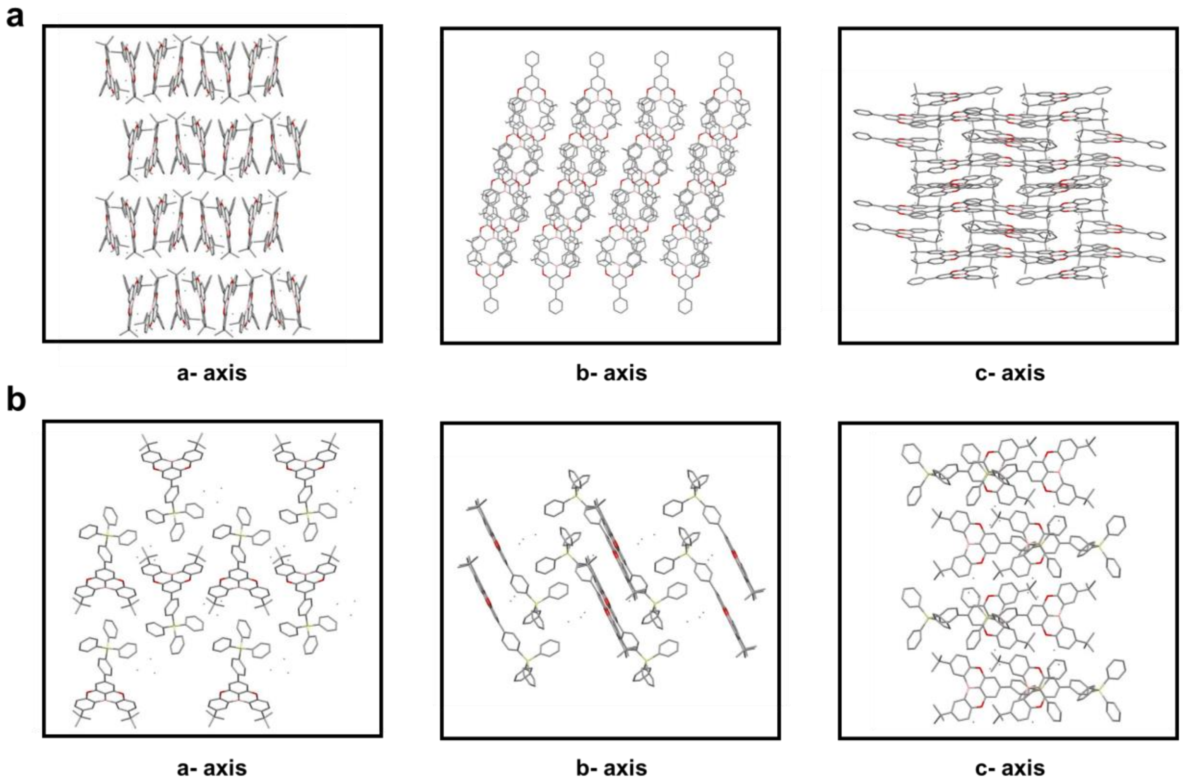

	Distance of plant to plane
TDBA-Ph	3.426 Å
TDBA-Si	4.197 Å

Supplementary Fig. S1 Single crystal XRD analysis. **a** TDBA-Ph. **b** TDBA-Si.

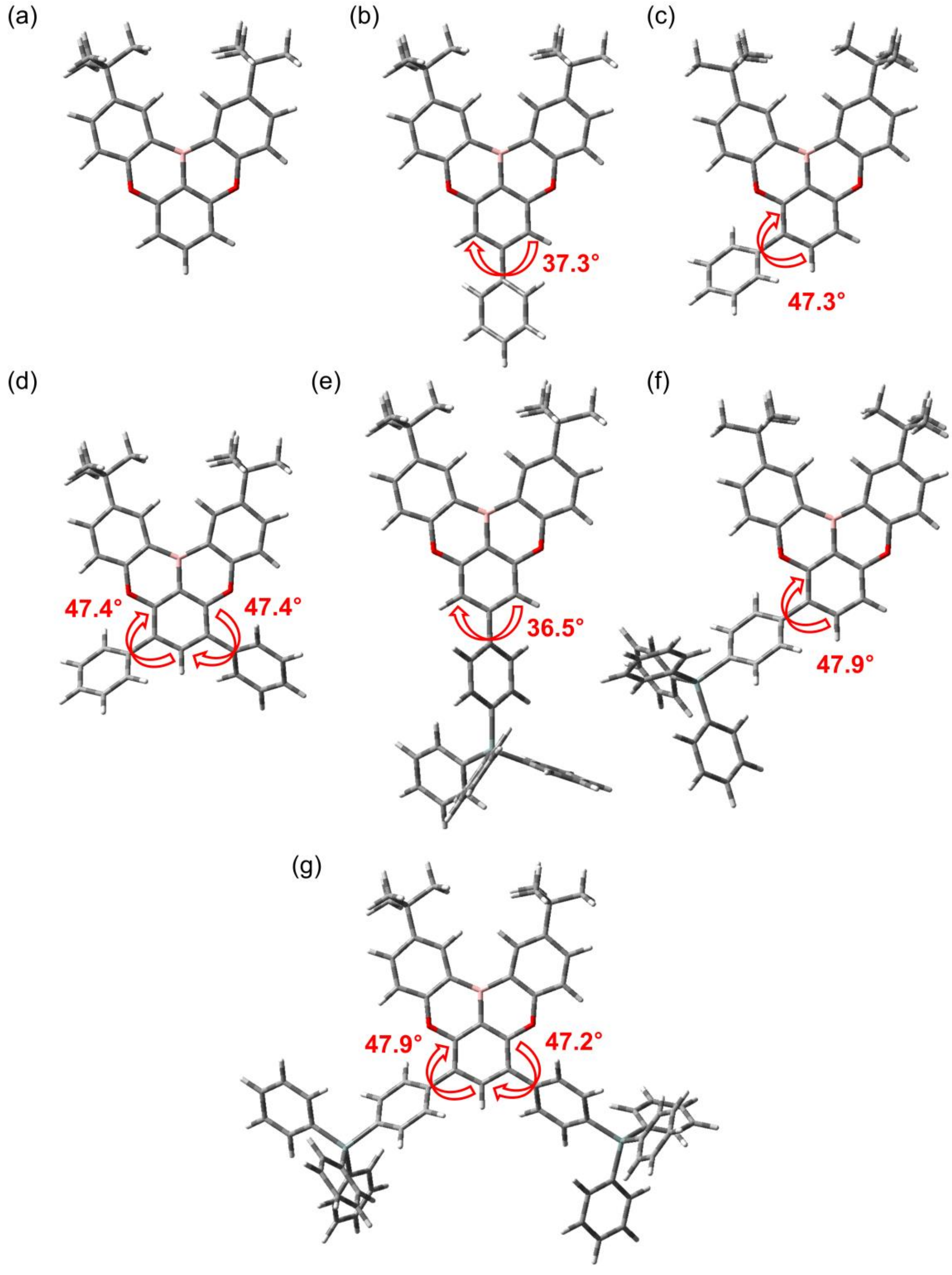

Supplementary Fig. S2 Optimized (B3LYP/6-31g(d,p)) geometries at the ground state and dihedral angle between TDBA core and peripheral side groups (phenyl and tetraphenylsilane). a TDBA. **b** TDBA-Ph. **c** mTDBA-Ph. **d** mTDBA-2Ph. **e** TDBA-Si. **f** mTDBA-Si. **g** mTDBA-2Si.

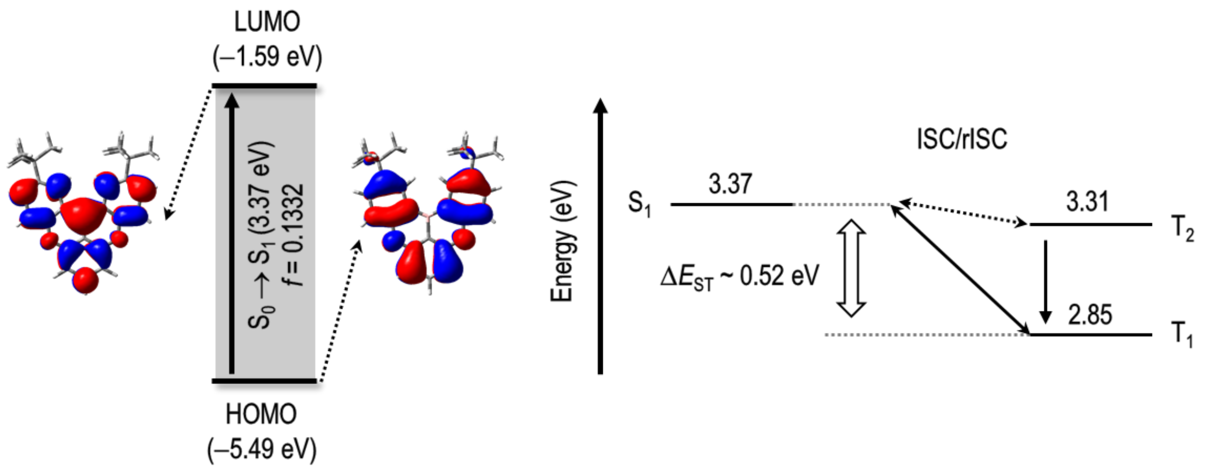

Supplementary Fig. S3 Isosurface of HOMO and LUMO composing $S_0 \rightarrow S_1$ transition (isovalue = 0.02) with representative electronic transition energies with SOC values of TDBA. TD-B3LYP calculation was conducted at the level of 6-31G(d,p).

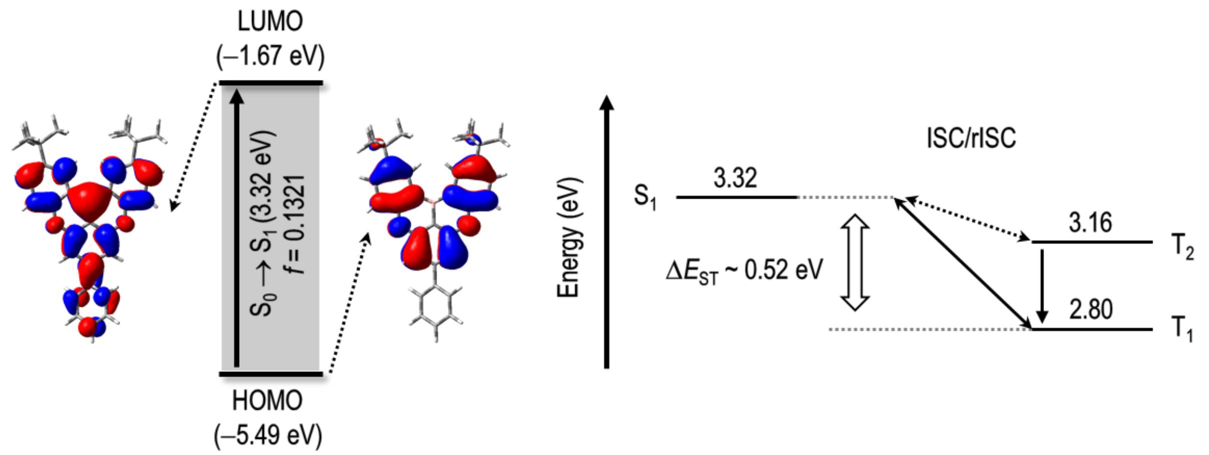

Supplementary Fig. S4 Isosurface of HOMO and LUMO composing $S_0 \rightarrow S_1$ transition (isovalue = 0.02) with representative electronic transition energies with SOC values of TDBA-Ph. TD-B3LYP calculation was conducted at the level of 6-31G(d,p).

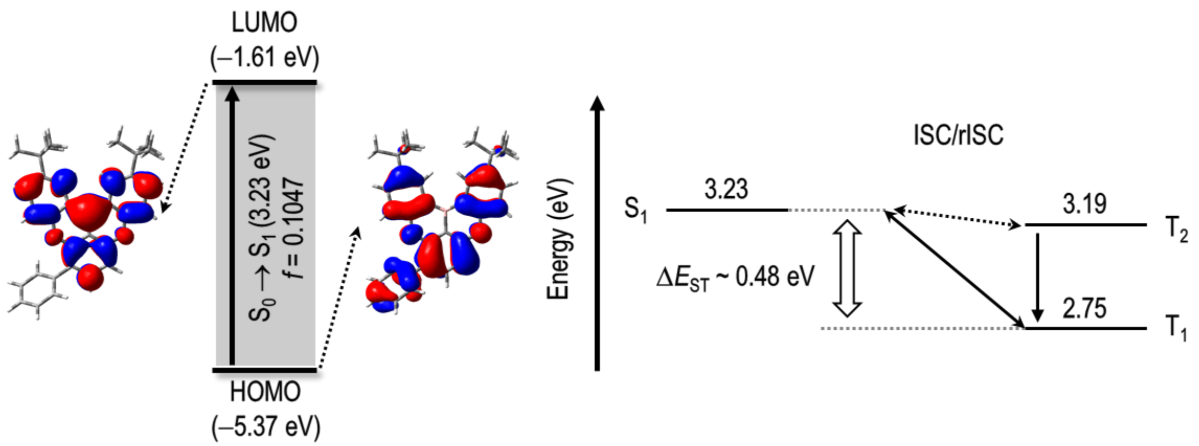

Supplementary Fig. S5 Isosurface of HOMO and LUMO composing $S_0 \rightarrow S_1$ transition (isovalue = 0.02) with representative electronic transition energies with SOC values of mTDBA-Ph. TD-B3LYP calculation was conducted at the level of 6-31G(d,p).

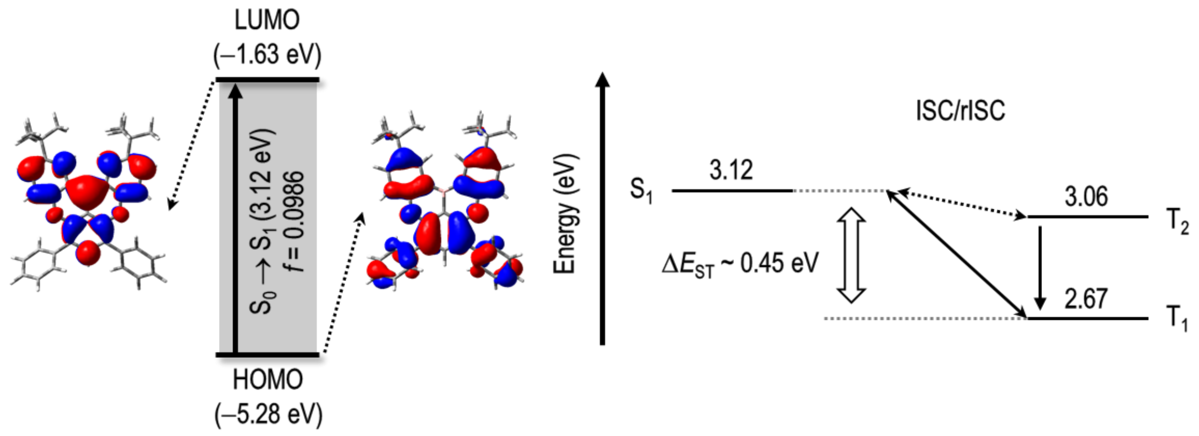

Supplementary Fig. S6 Isosurface of HOMO and LUMO composing $S_0 \rightarrow S_1$ transition (isovalue = 0.02) with representative electronic transition energies with SOC values of mTDBA-2Ph. TD-B3LYP calculation was conducted at the level of 6-31G(d,p).

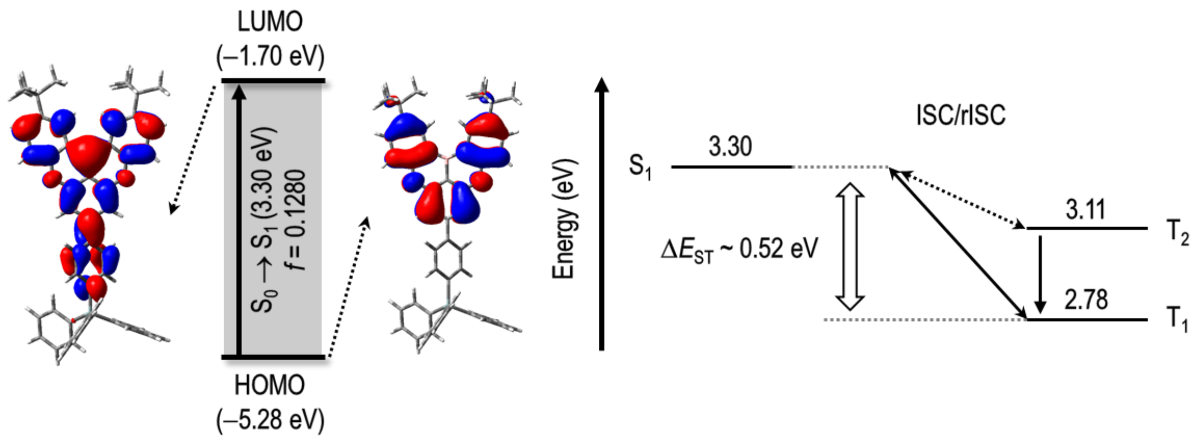

Supplementary Fig. S7 Isosurface of HOMO and LUMO composing $S_0 \rightarrow S_1$ transition (isovalue = 0.02) with representative electronic transition energies with SOC values of TDBA-Si. TD-B3LYP calculation was conducted at the level of 6-31G(d,p).

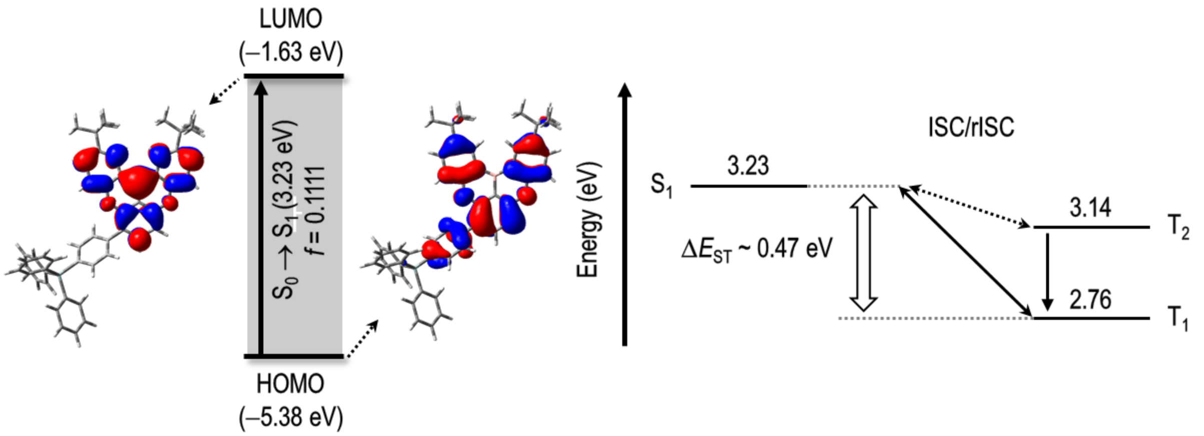

Supplementary Fig. S8 Isosurface of HOMO and LUMO composing $S_0 \rightarrow S_1$ transition (isovalue = 0.02) with representative electronic transition energies with SOC values of mTDBA-Si. TD-B3LYP calculation was conducted at the level of 6-31G(d,p).

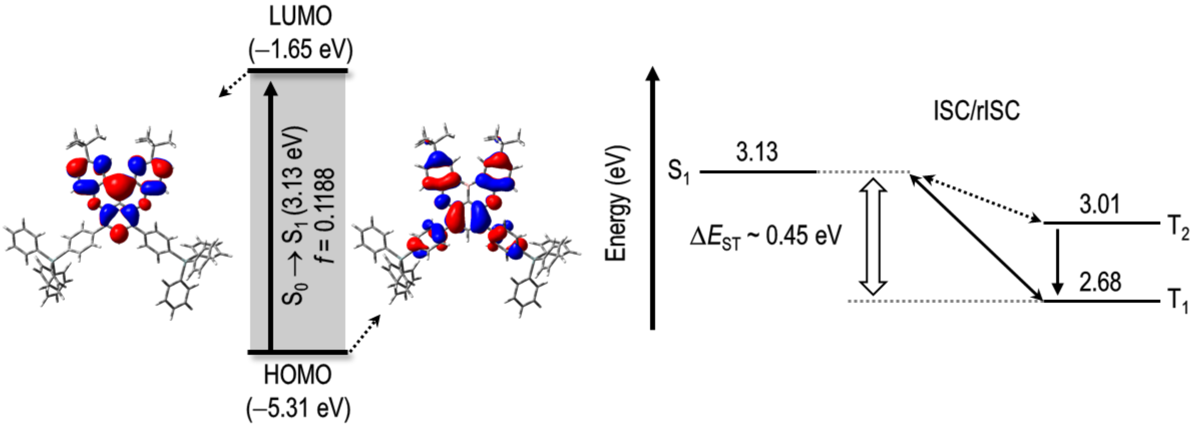

Supplementary Fig. S9 Isosurface of HOMO and LUMO composing $S_0 \rightarrow S_1$ transition (isovalue = 0.02) with representative electronic transition energies with SOC values of mTDBA-Si. TD-B3LYP calculation was conducted at the level of 6-31G(d,p).

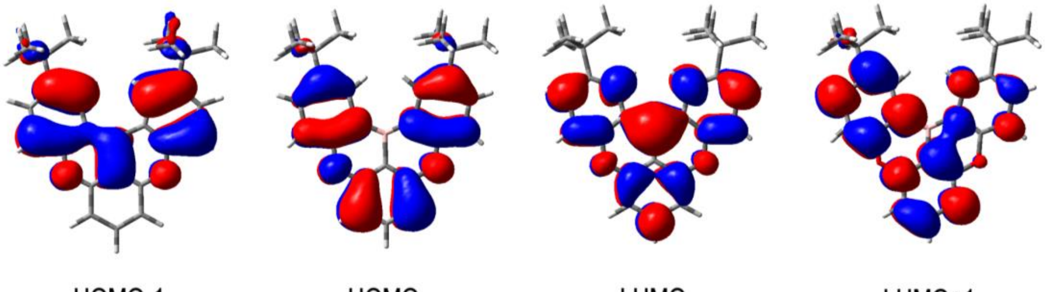

state	energy (eV)	participating molecular orbitals	transition character
T_1	2.85	HOMO → LUMO (96%)	$\pi \rightarrow \pi^*$
T_2	3.31	HOMO-1 → LUMO (58%)	$\pi \rightarrow \pi^*$
S_1	3.37	HOMO → LUMO (98%)	$\pi \rightarrow \pi^*$

Only molecular orbitals of transition more than 10% were described in the table. Please note that if there are no orbital transitions above 10%, only the three highest orbital transitions are indicated.

Supplementary Fig. S10 Calculated electronic transitions and each transition character of TDBA.

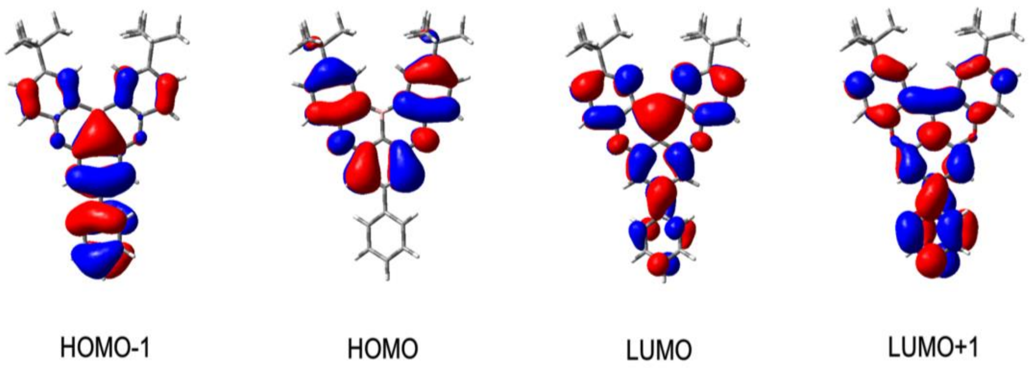

state	energy (eV)	participating molecular orbitals	transition character
T_1	2.80	HOMO → LUMO (97%)	$\pi\rightarrow\pi^*$
T_2	3.16	HOMO-1 → LUMO (62%)	$\pi\rightarrow\pi^*$
S_1	3.32	HOMO → LUMO (97%)	$\pi\rightarrow\pi^*$

Only molecular orbitals of transition more than 10% were described in the table. Please note that if there are no orbital transitions above 10%, only the three highest orbital transitions are indicated.

Supplementary Fig. S11 Calculated electronic transitions and each transition character of TDBA-Ph.

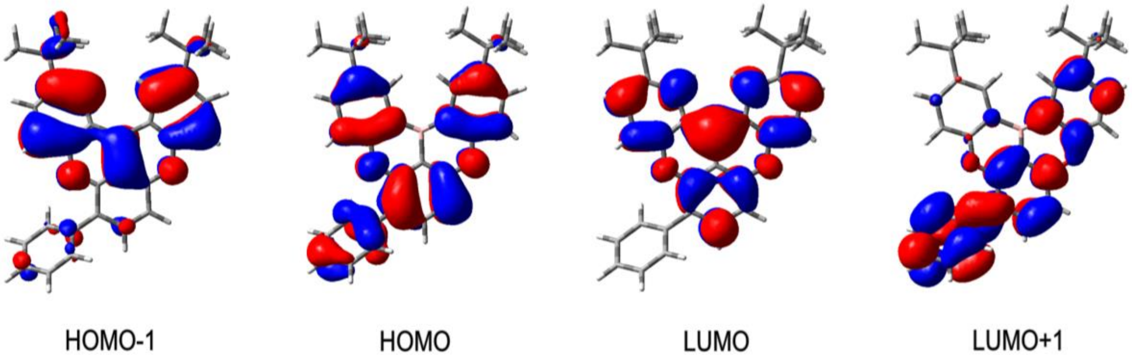

state	energy (eV)	participating molecular orbitals	transition character
T_1	2.75	HOMO → LUMO (96%)	π→π*
T_2	3.19	HOMO → LUMO+1 (47%), HOMO-1 → LUMO (15%)	π→π* / partial CT
S_1	3.23	HOMO → LUMO (98%)	π→π*

Only molecular orbitals of transition more than 10% were described in the table. Please note that if there are no orbital transitions above 10%, only the three highest orbital transitions are indicated.

Supplementary Fig. S12 Calculated electronic transitions and each transition character of mTDBA-Ph.

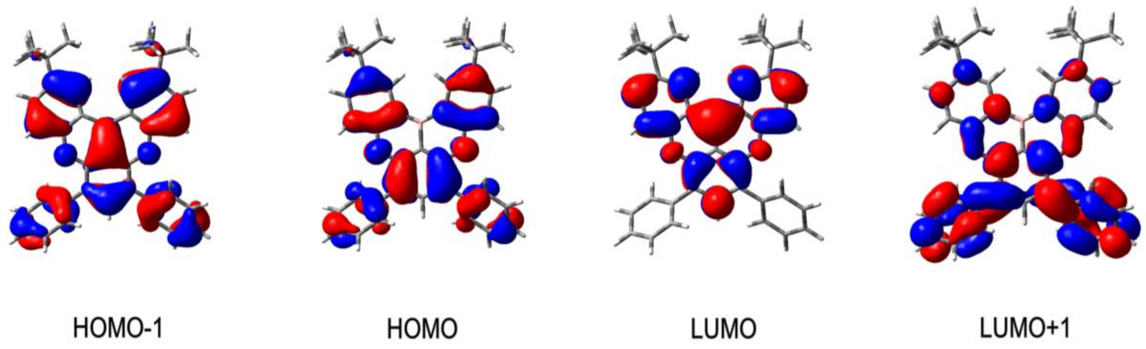

state	energy (eV)	participating molecular orbitals	transition character
T_1	2.67	HOMO → LUMO (96%)	$\pi\rightarrow\pi^*$
T_2	3.06	HOMO → LUMO+1 (63%)	$\pi\rightarrow\pi^*$
S_1	3.12	HOMO → LUMO (98%)	$\pi\rightarrow\pi^*$

Only molecular orbitals of transition more than 10% were described in the table. Please note that if there are no orbital transitions above 10%, only the three highest orbital transitions are indicated.

Supplementary Fig. S13 Calculated electronic transitions and each transition character of mTDBA-2Ph.

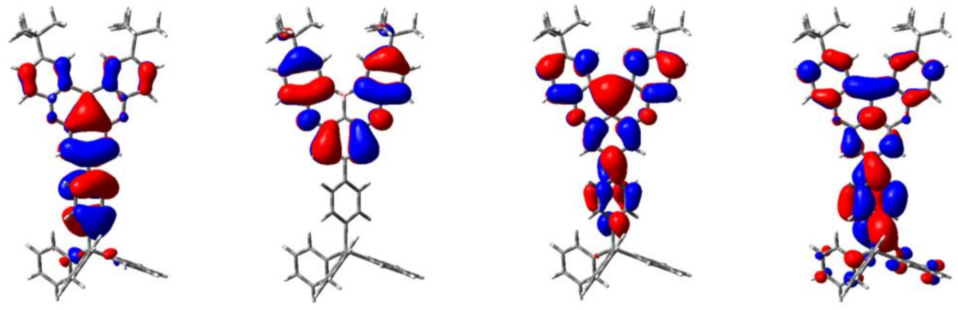

state	energy (eV)	participating molecular orbitals	transition character
T_1	2.78	HOMO → LUMO (97%)	$\pi\rightarrow\pi^*$
T_2	3.11	HOMO-1 → LUMO (62%), HOMO-1 → LUMO+1 (12%)	$\pi\rightarrow\pi^*$
S_1	3.30	HOMO → LUMO (97%)	$\pi\rightarrow\pi^*$

Only molecular orbitals of transition more than 10% were described in the table. Please note that if there are no orbital transitions above 10%, only the three highest orbital transitions are indicated.

Supplementary Fig. S14 Calculated electronic transitions and each transition character of TDBA-Si.

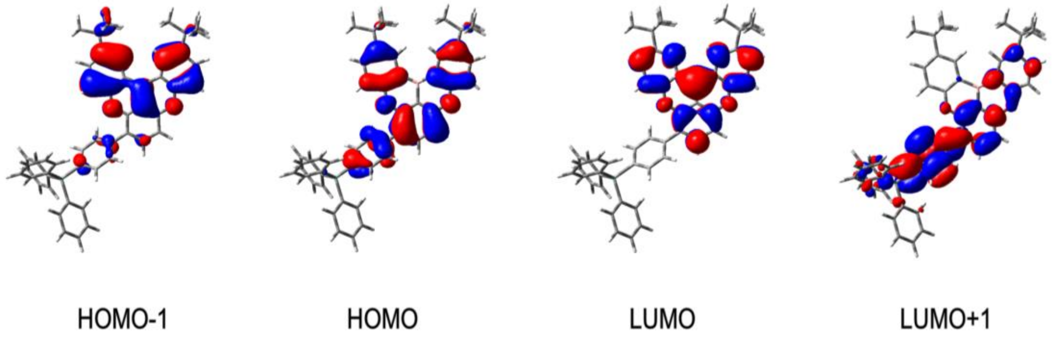

state	energy (eV)	participating molecular orbitals	transition character
T_1	2.76	HOMO → LUMO (96%)	$\pi \rightarrow \pi^*$
T_2	3.14	HOMO → LUMO+1 (54%)	$\pi \rightarrow \pi^*$ / partial CT
S_1	3.23	HOMO → LUMO (98%)	$\pi \rightarrow \pi^*$

Only molecular orbitals of transition more than 10% were described in the table. Please note that if there are no orbital transitions above 10%, only the three highest orbital transitions are indicated.

Supplementary Fig. S15 Calculated electronic transitions and each transition character of mTDBA-Si.

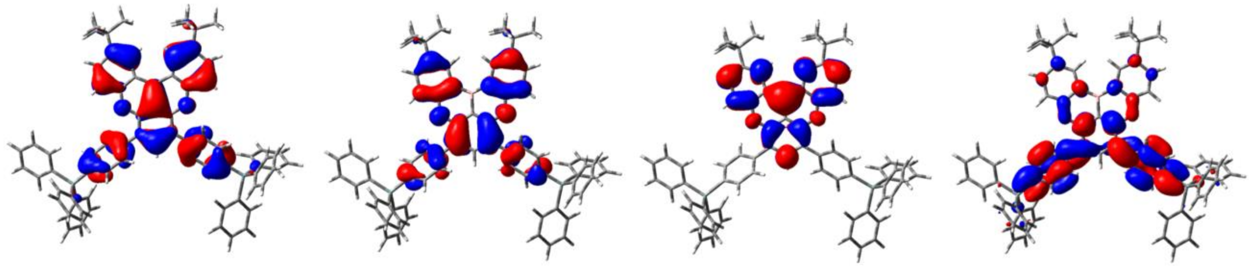

state	energy (eV)	participating molecular orbitals	transition character
T_1	2.68	HOMO → LUMO (96%)	$\pi \rightarrow \pi^*$
T_2	3.01	HOMO → LUMO+1 (64%)	$\pi \rightarrow \pi^*$
S_1	3.13	HOMO → LUMO (98%)	$\pi \rightarrow \pi^*$

Only molecular orbitals of transition more than 10% were described in the table. Please note that if there are no orbital transitions above 10%, only the three highest orbital transitions are indicated.

Supplementary Fig. S16 Calculated electronic transitions and each transition character of mTDBA-2Si.

(a)

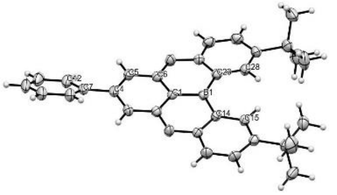

Selected torsion angles
C1-B1-C23-C28 178.2(3)°
C1-B1-C14-C15 -177.7(3)°
C5-C4-C7-C12 29.0(4)°

(b)

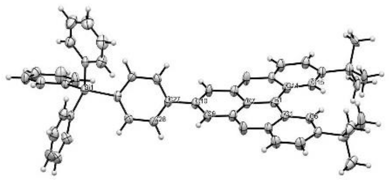

Selected torsion angles
C6-C1-B1-C7 -179.9(3)°
C15-C14-B1-C7 -177.7(3)°
C9-C10-C27-C28 -40.2(5)°

(c)

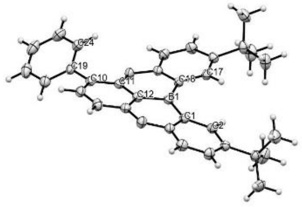

Selected torsion angles
C12-B1-C18-C17 164.7(1)°
C12-B1-C1-C2 168.8(1)°
C11-C10-C19-C20 137.6(2)°

(d)

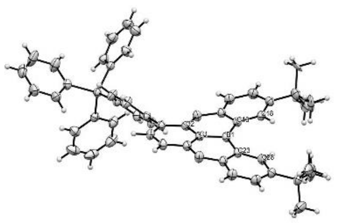

Selected torsion angles
C1-B1-C13-C18 166.5(2)°
C1-B1-C23-C24 -8.7(2)°
C2-C3-C7-C8 44.9(3)°

Supplementary Fig. S17 Single-crystal XRD structures with Oak Ridge Thermal Ellipsoid Plot (ORTEP) drawing of the selected materials with 50% probability level. **a** TDBA-Ph. **b** TDBA-Si **c** mTDBA-Ph. **d** mTDBA-Si.

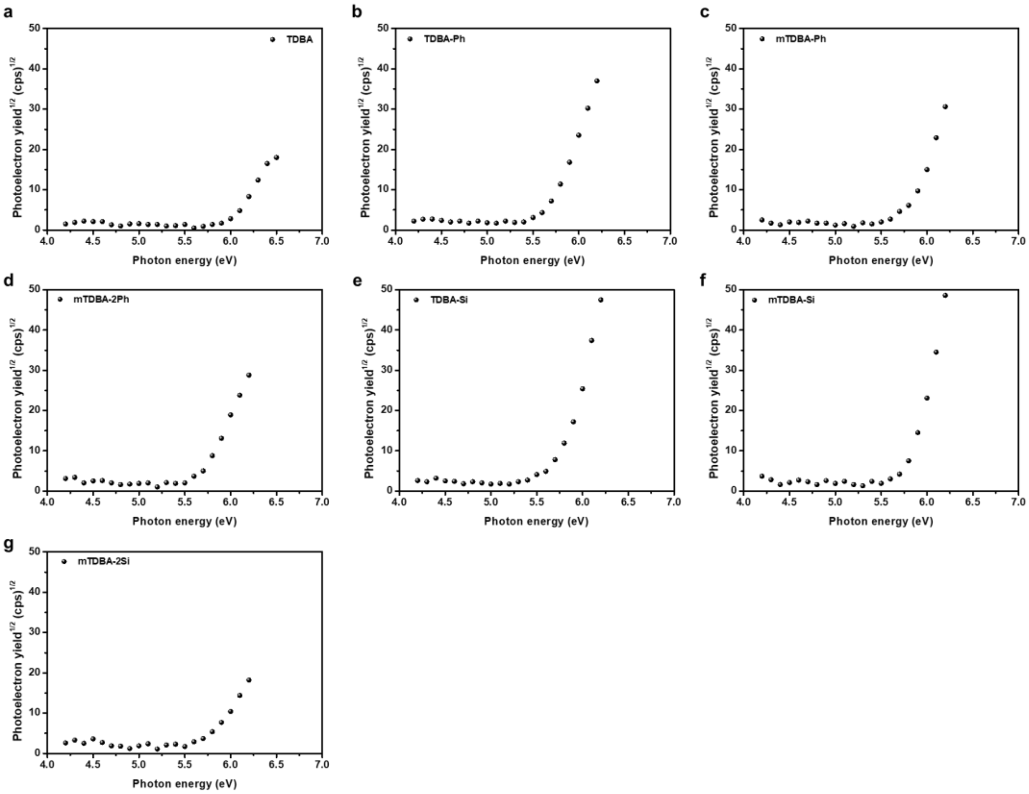

Supplementary Fig. S18 Photoelectron spectroscopy (AC-2) of TDBA-based host materials. a TDBA. **b** TDBA-Ph. **c** mTDBA-Ph. **d** mTDBA-2Ph. **e** TDBA-Si. **f** mTDBA-Si. **g** mTDBA-2Si.

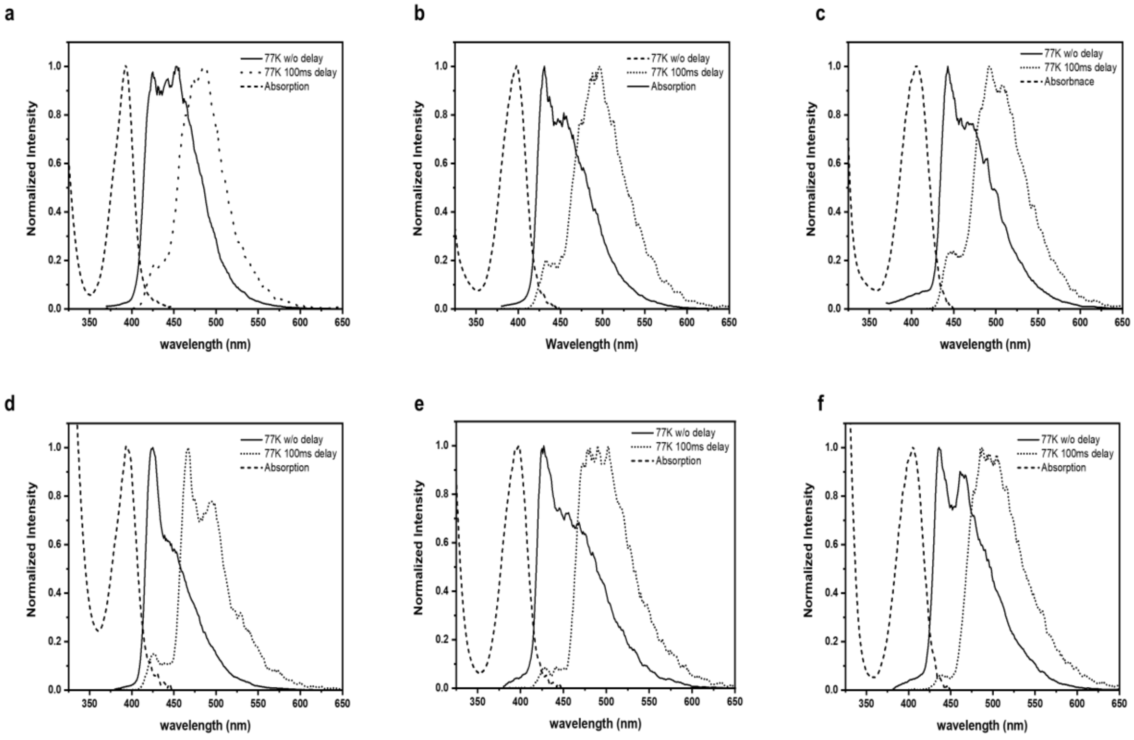

Supplementary Fig. S19 Absorption spectra(dash) at room temperature, low temeperature photoluminescence without (line) and with delay (dot) at 77K spectra of neat film. **a** TDBA-Ph. **b** mTDBA-Ph. **c** mTDBA-2Ph. **d** TDBA-Si. **e** mTDBA-Si. **f** mTDBA-2Si.

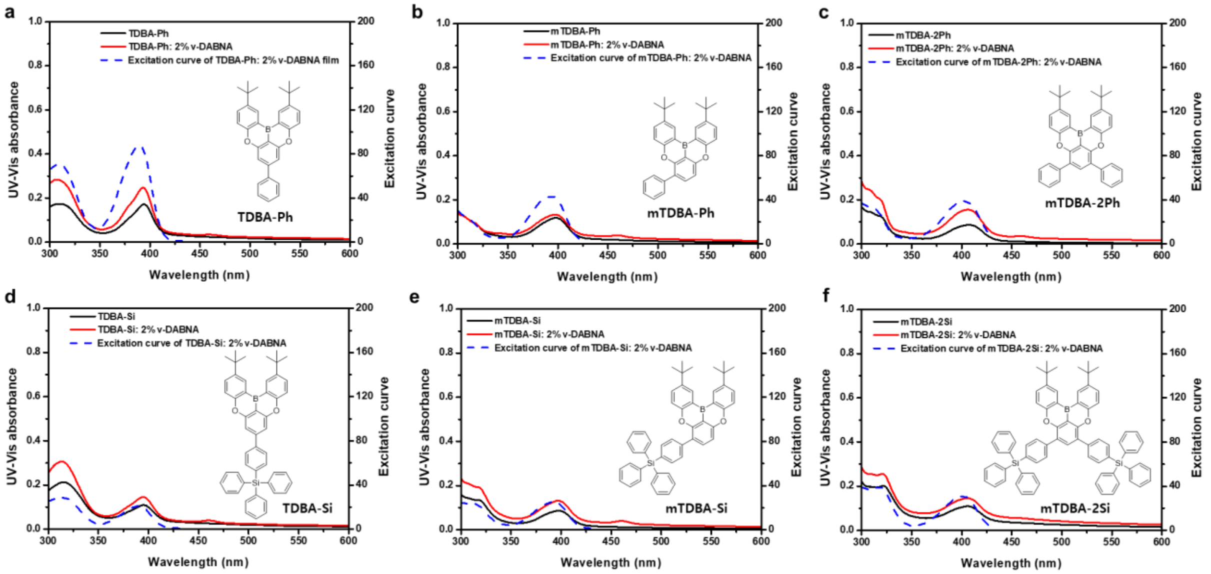

Supplementary Fig. S20 UV-Visible absorption and excitation spectra of 2% v-DABNA films using TDBA-based host materials. **a** TDBA-Ph. **b** mTDBA-Ph. **c** mTDBA-2Ph. **d** TDBA-Si. **e** mTDBA-Si. **f** mTDBA-2Si.

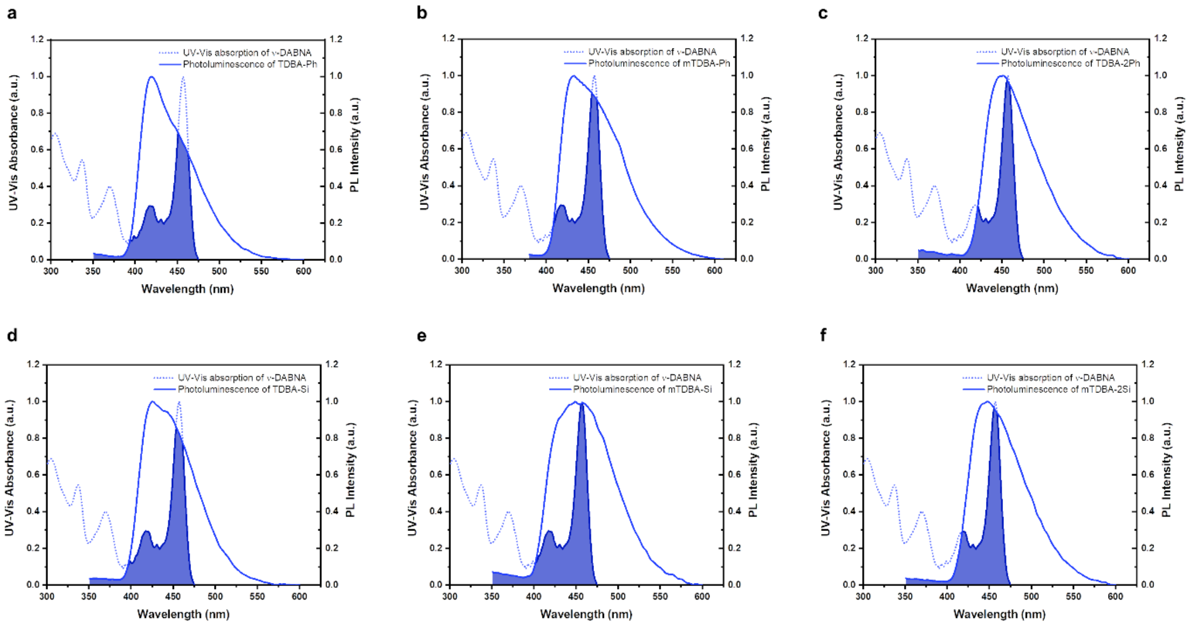

Supplementary Fig. S21 Spectral overlap between ν-DABNA and TDBA-based host. a TDBA-Ph. **b** mTDBA-Ph. **c** mTDBA-2Ph. **d** TDBA-Si. **e** mTDBA-Si. **f** mTDBA-2Si.

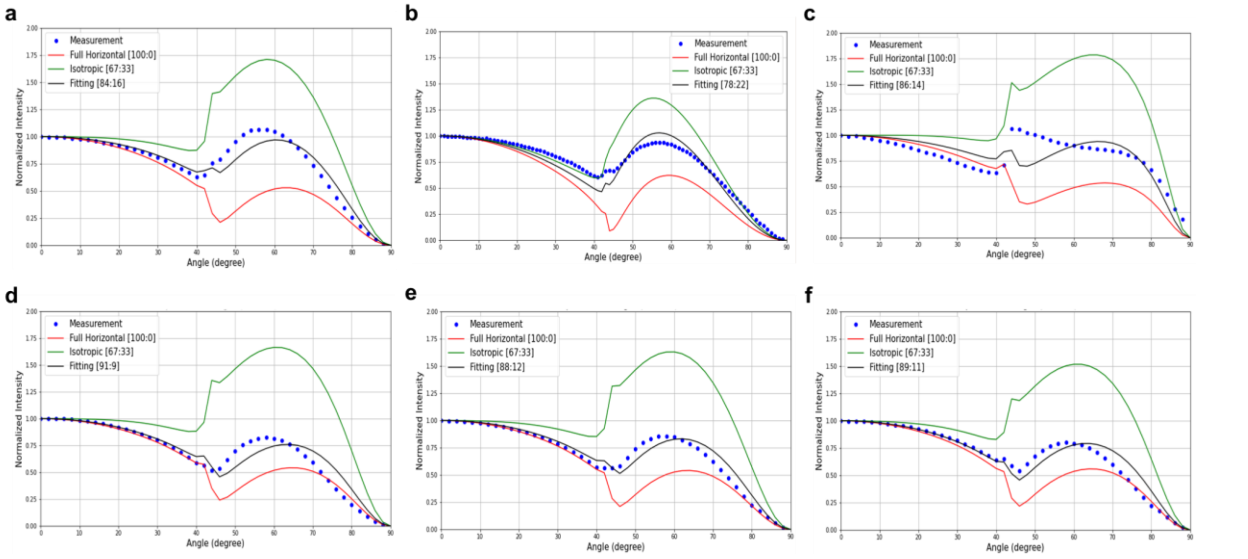

Supplementary Fig. S22 Molecule orientation in doping films according to TDBA-host materials. a TDBA-Ph. **b** mTDBA-Ph. **c** mTDBA-2Ph. **d** TDBA-Si. **e** mTDBA-Si. **f** mTDBA-2Si.

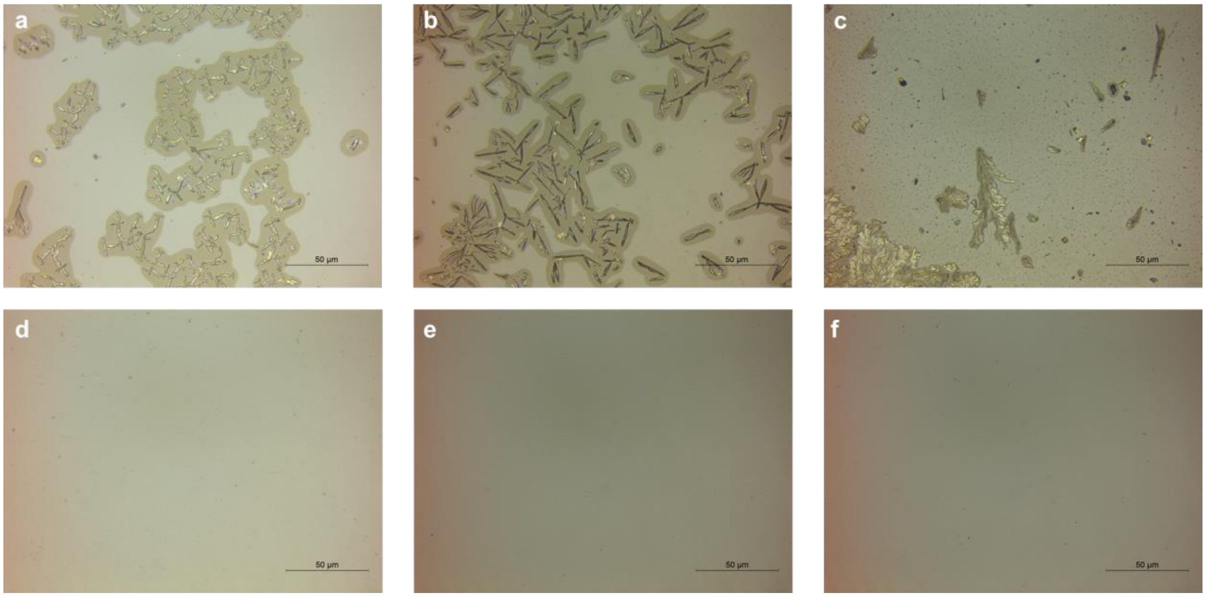

Supplementary Fig. S23 Optical microscopy image of the 2 wt% ν-DABNA-doped TDBA-based film. **a** TDBA-Ph. **b** mTDBA-Ph. **c** mTDBA-2Ph. **d** TDBA-Si. **e** mTDBA-Si. **f** mTDBA-2Si.

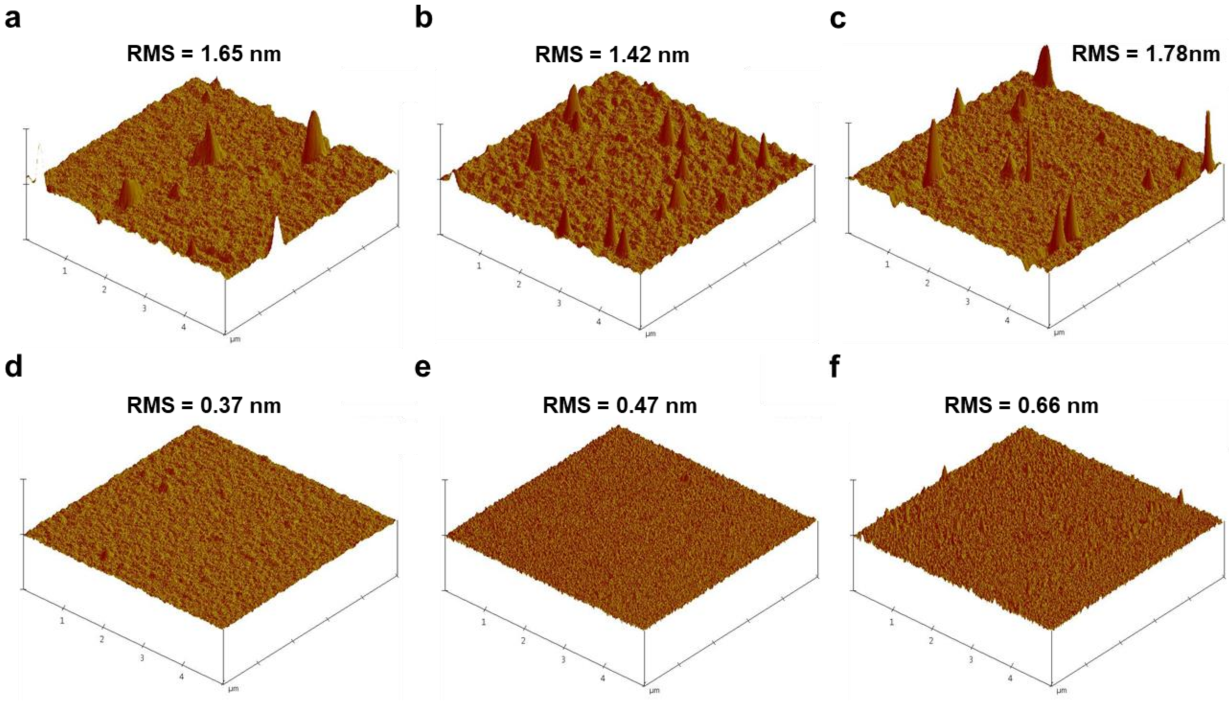

Supplementary Fig. S24 AFM image of the 2 wt% ν-DABNA-doped TDBA-based film. **a** TDBA-Ph. **b** mTDBA-Ph. **c** mTDBA-2Ph. **d** TDBA-Si. **e** mTDBA-Si. **f** mTDBA-2Si.

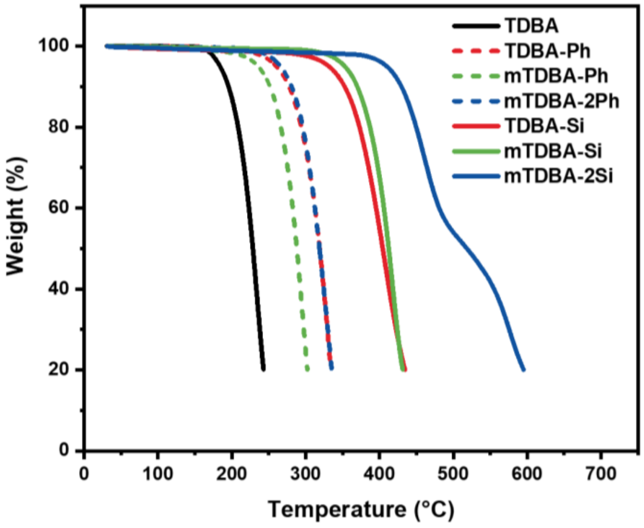

Supplementary Fig. S25 Thermo gravimetric analyzer (TGA) results of TDBA-based host materials.

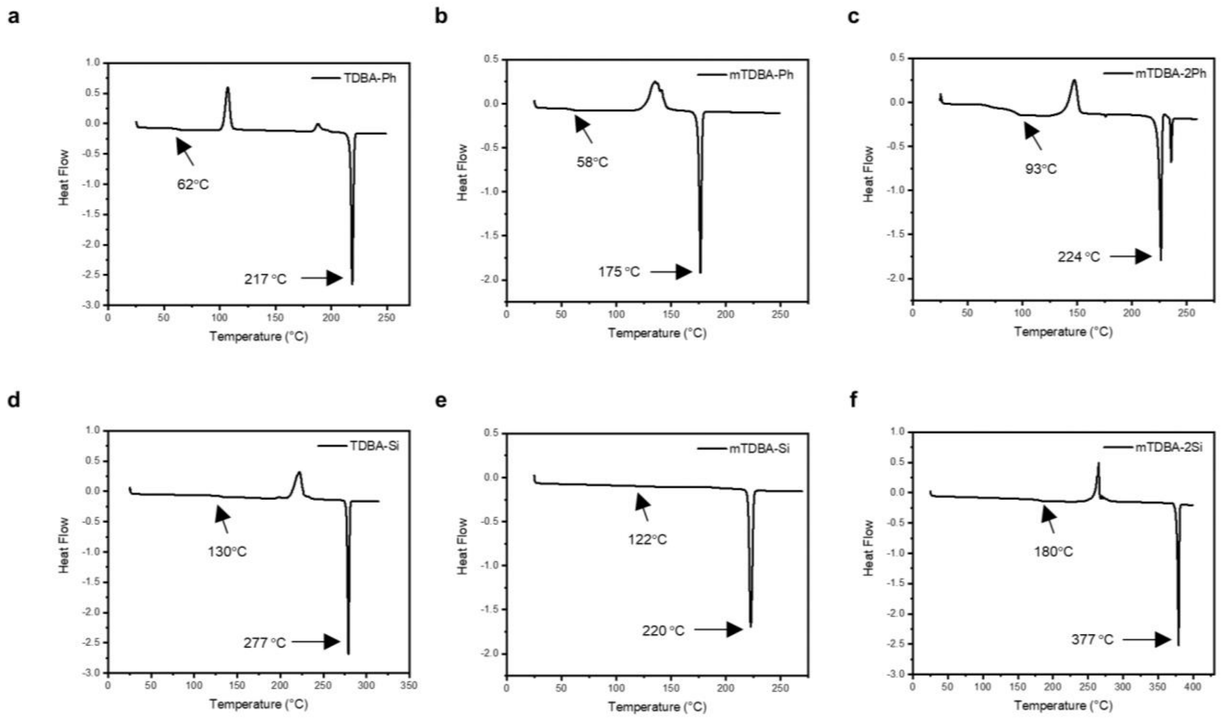

Supplementary Fig. S26 Differential scanning calorimetry (DSC) results of TDBA-based host materials. **a** TDBA-Ph. **b** mTDBA-Ph. **c** mTDBA-2Ph. **d** TDBA-Si. **e** mTDBA-Si. **f** mTDBA-2Si.

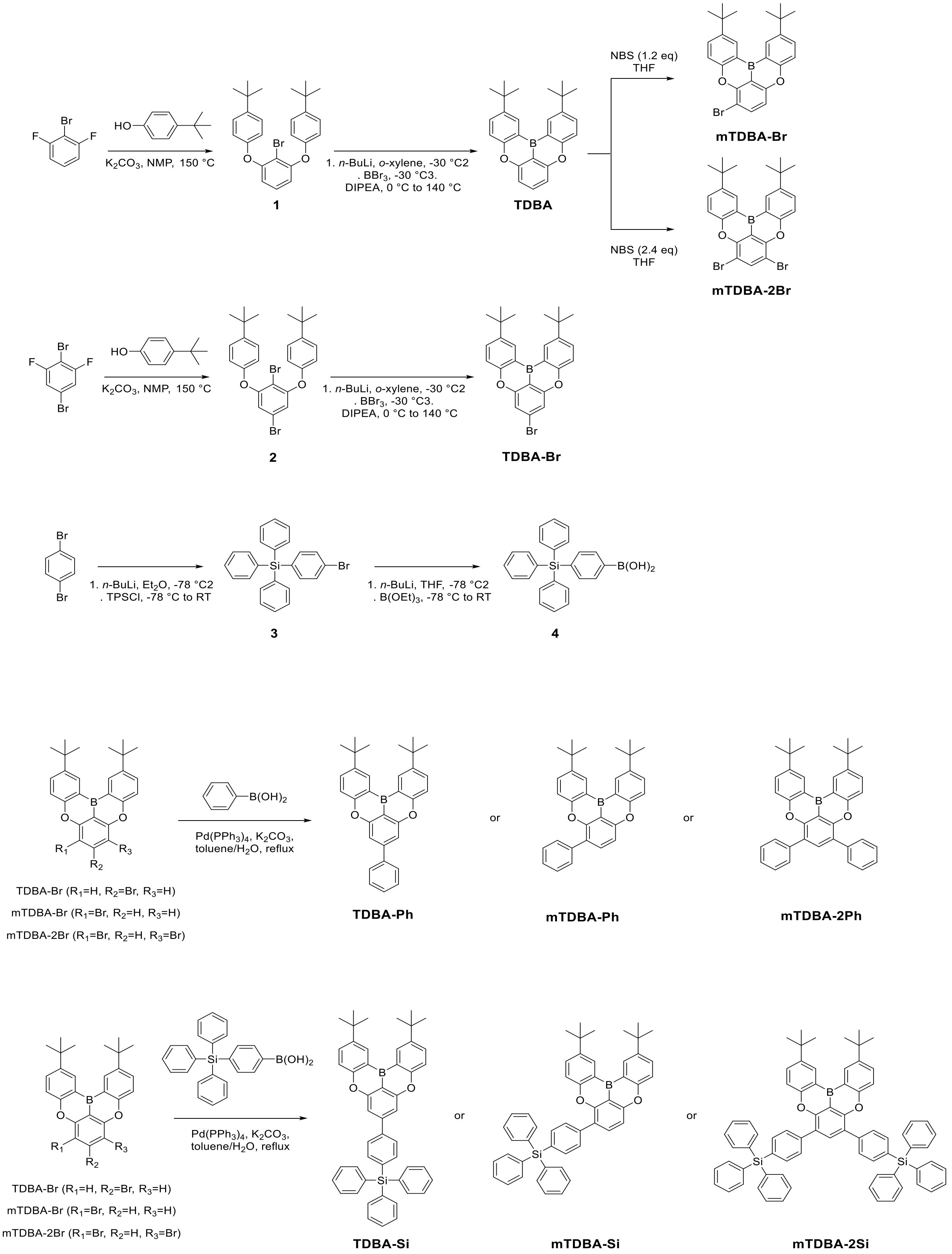

Supplementary Fig. S27 Synthetic route of TDBA-based host materials.

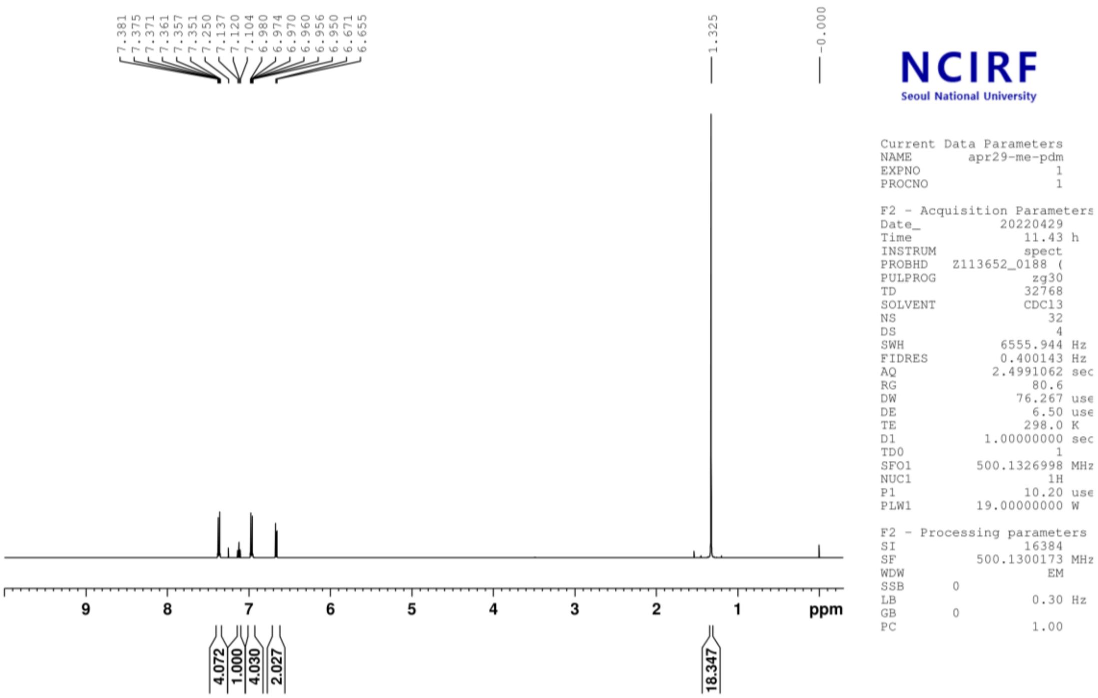

Supplementary Fig. S28 ^{1}H-NMR spectra of **4,4'-((2-bromo-1,3-phenylene)bis(oxy))bis(*tert*-butylbenzene).**

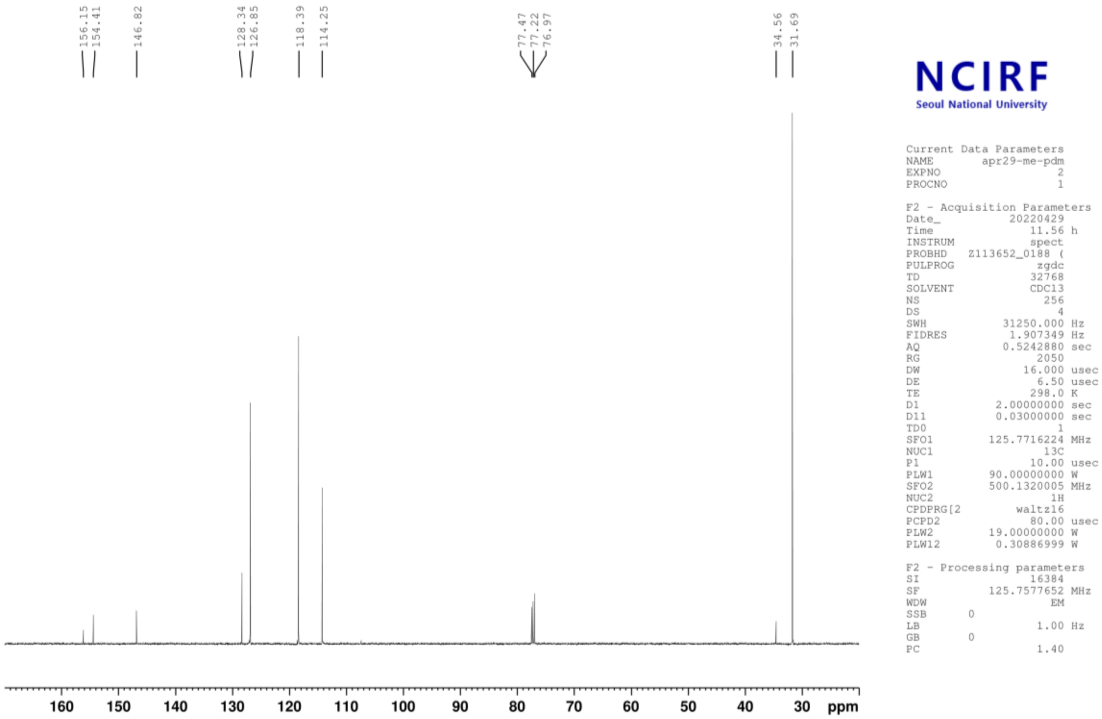

Supplementary Fig. S29 ^{13}C-NMR spectra of **4,4'-((2-bromo-1,3-phenylene)bis(oxy))bis(*tert*-butylbenzene).**

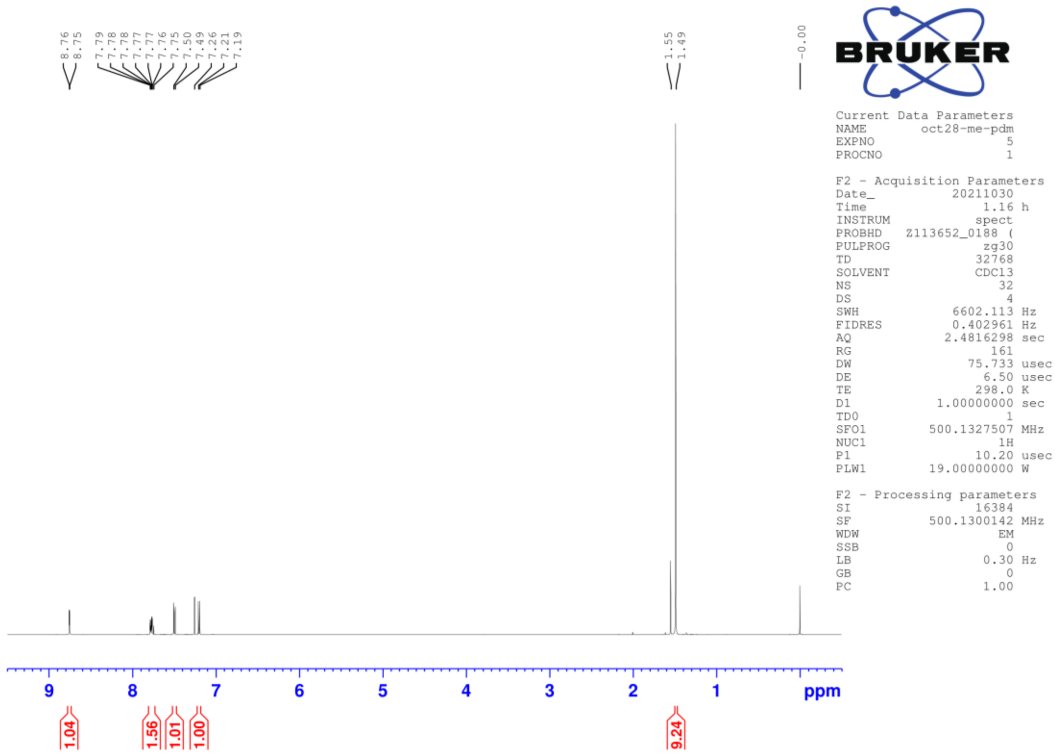

Supplementary Fig. S30 ^{1}H-NMR spectra of **TDBA.**

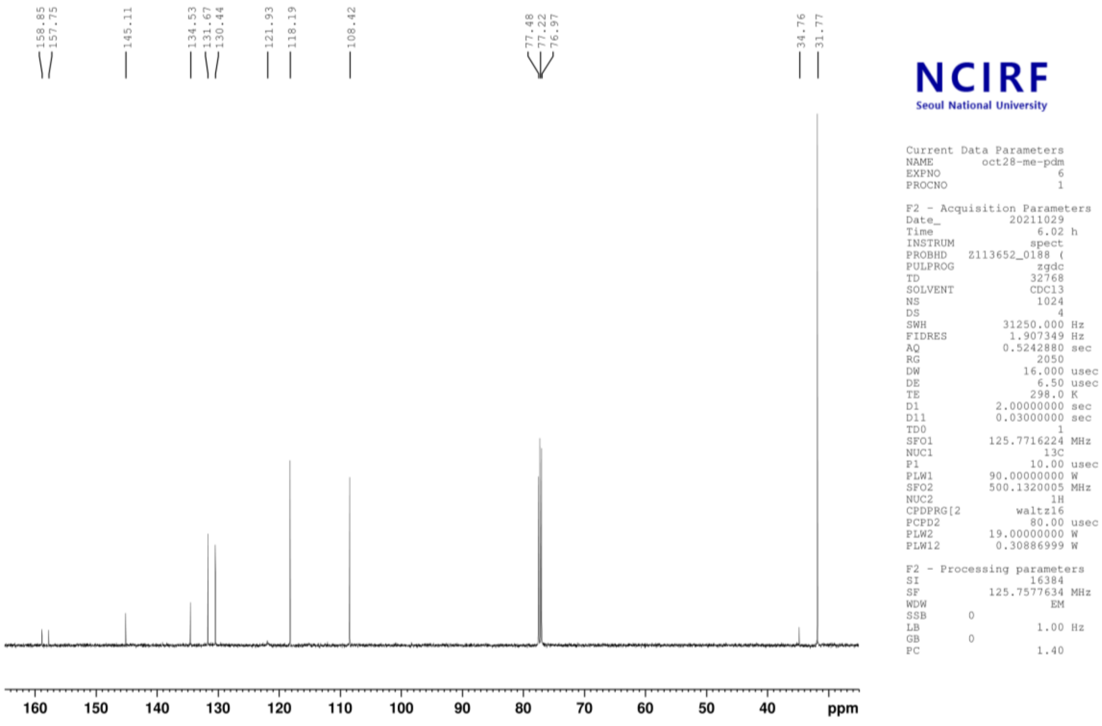

Supplementary Fig. S31 ^{13}C-NMR spectra of **TDBA.**

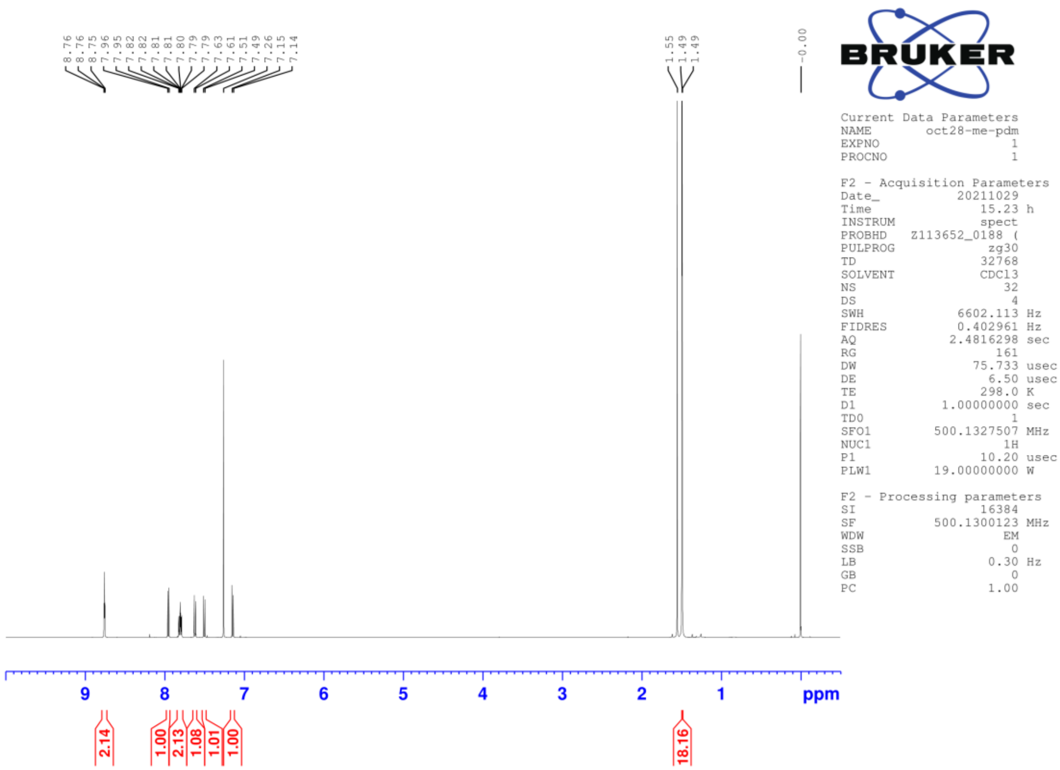

Supplementary Fig. S32 ^{1}H-NMR spectra of **mTDBA-Br.**

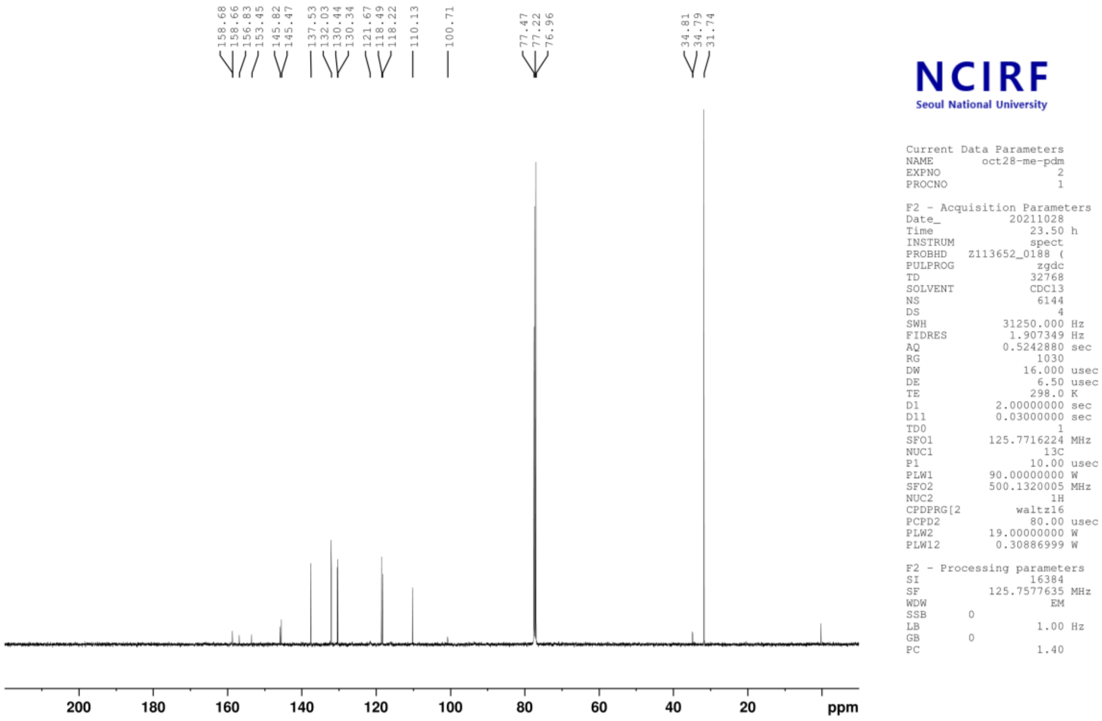

Supplementary Fig. S33 ^{13}C-NMR spectra of **mTDBA-Br.**

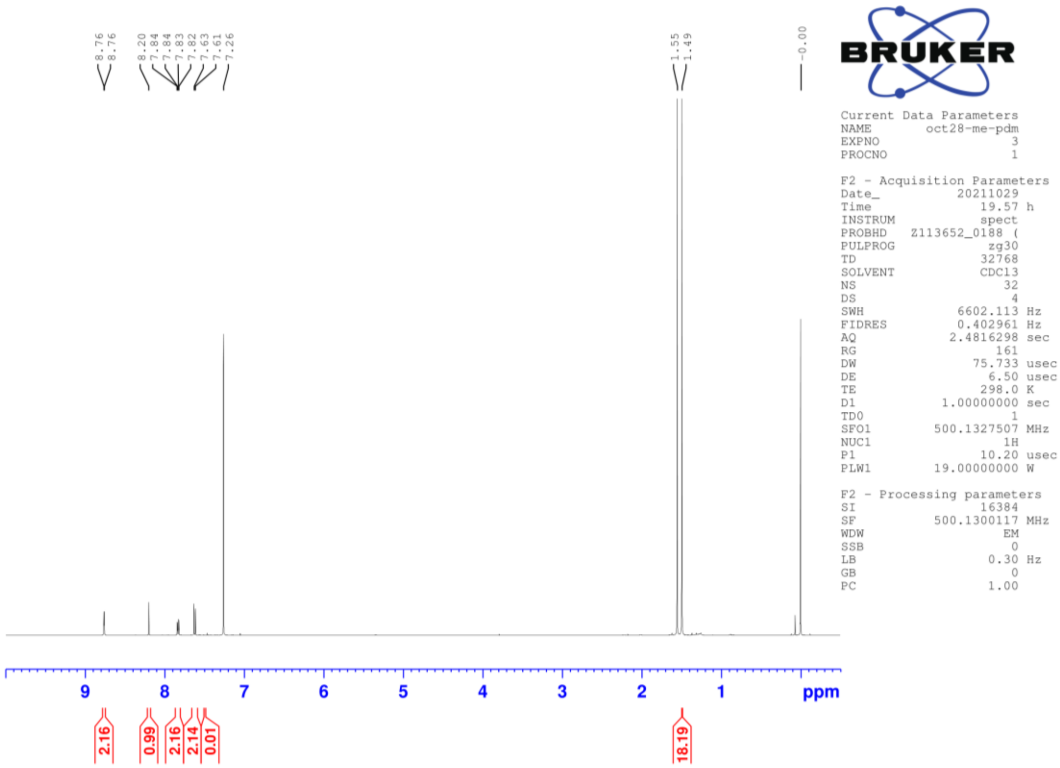

Supplementary Fig. S34 ^{1}H-NMR spectra of **mTDBA-2Br.**

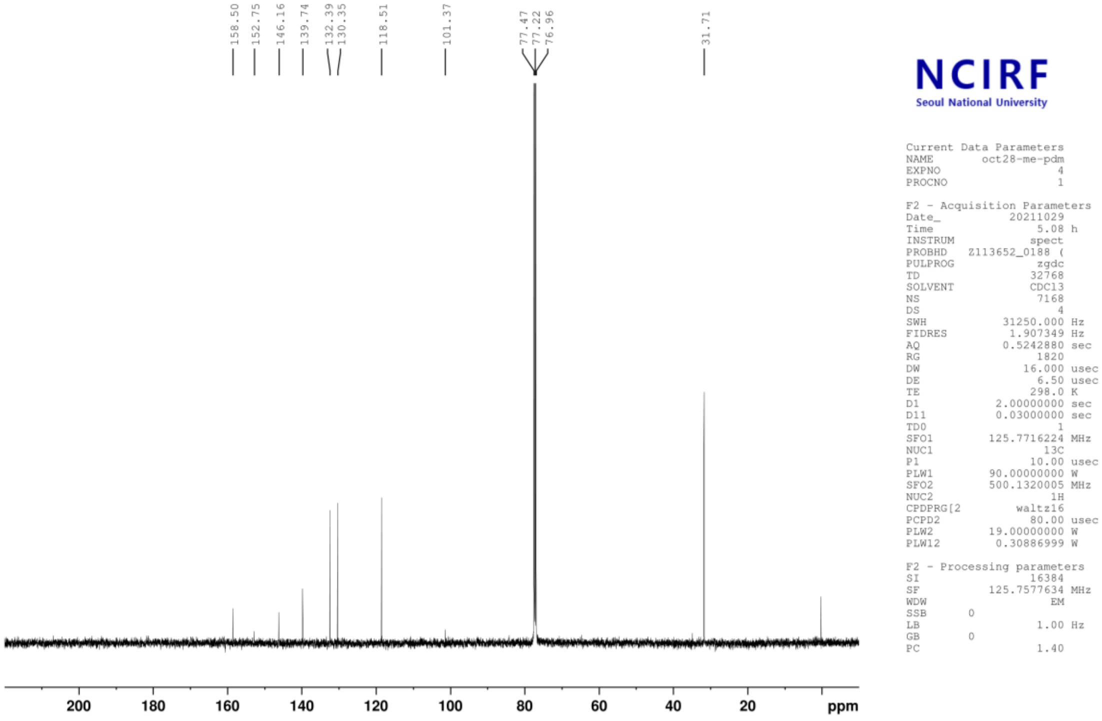

Supplementary Fig. S35 ^{13}C-NMR spectra of **mTDBA-2Br.**

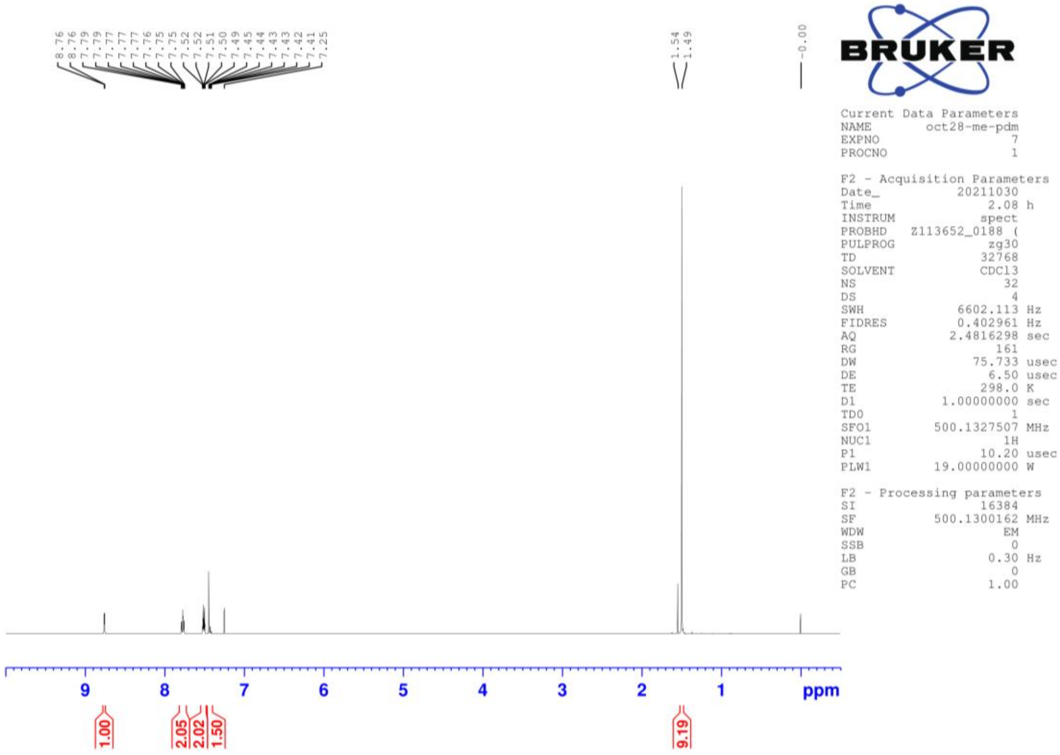

Supplementary Fig. S36 ^{1}H-NMR spectra of **TDBA-Ph.**

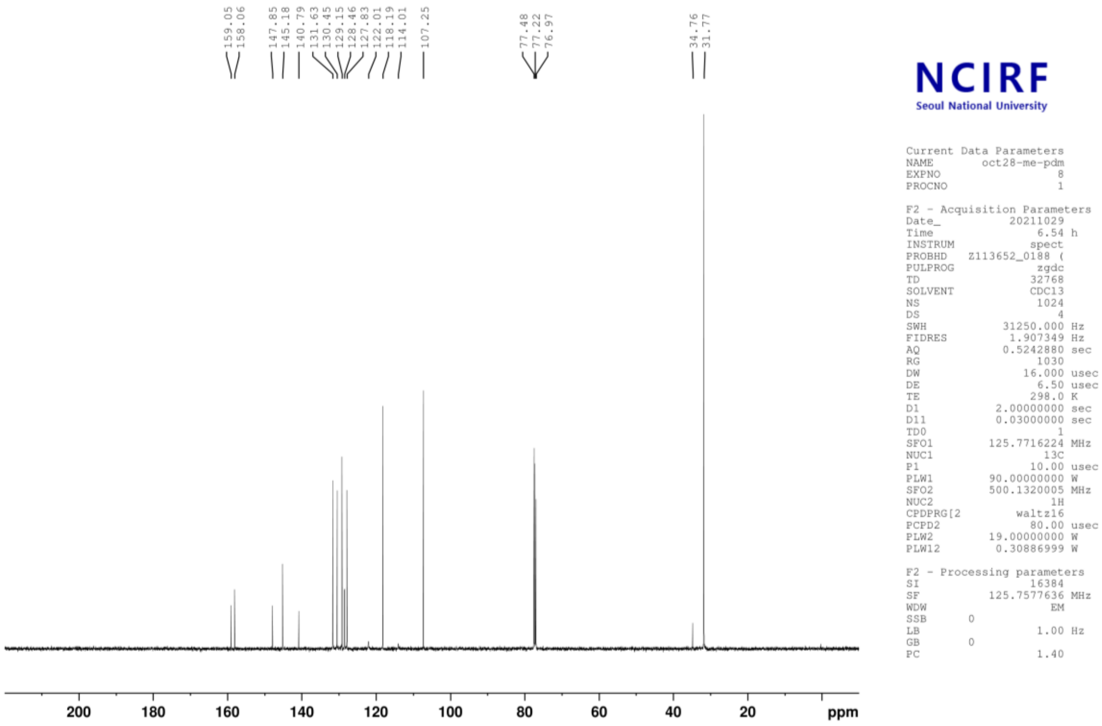

Supplementary Fig. S37 ^{13}C-NMR spectra of **TDBA-Ph.**

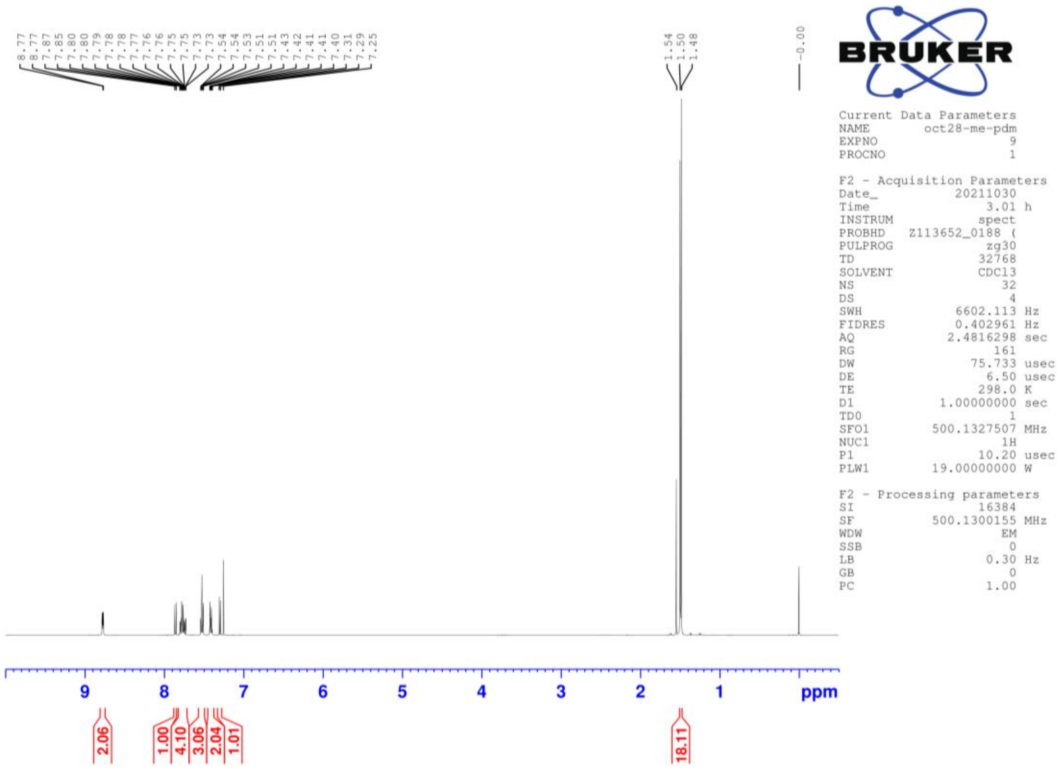

Supplementary Fig. S38 ^{1}H-NMR spectra of **mTDBA-Ph.**

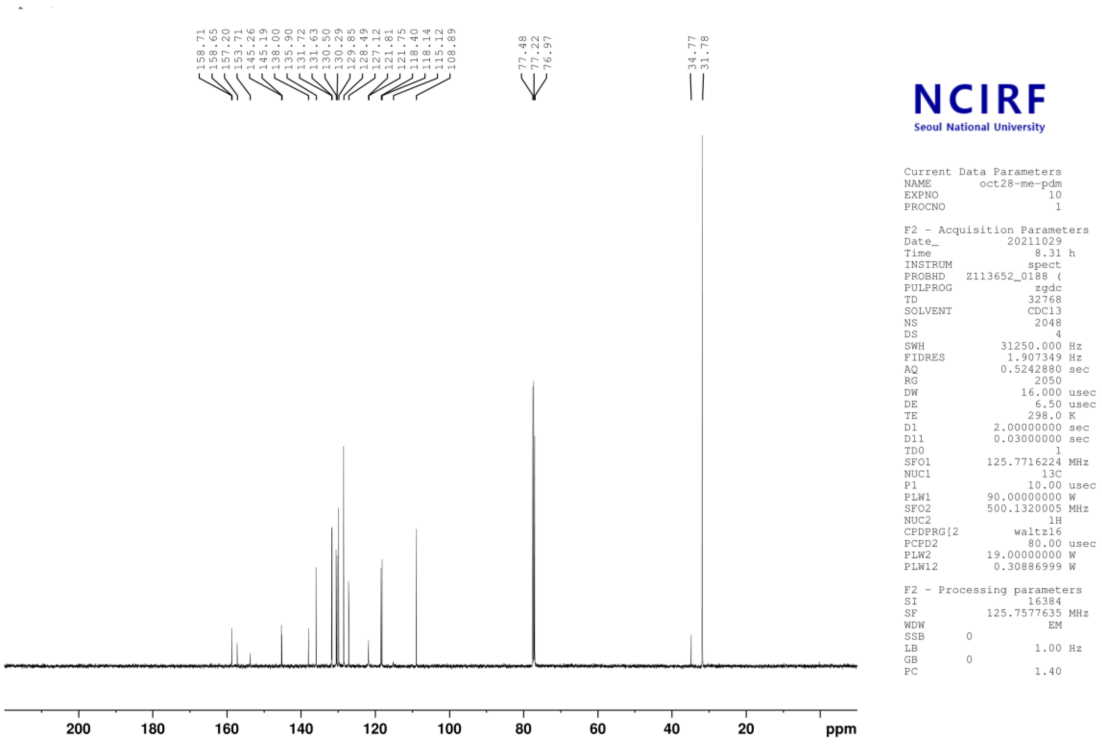

Supplementary Fig. S39 ^{13}C-NMR spectra of **mTDBA-Ph.**

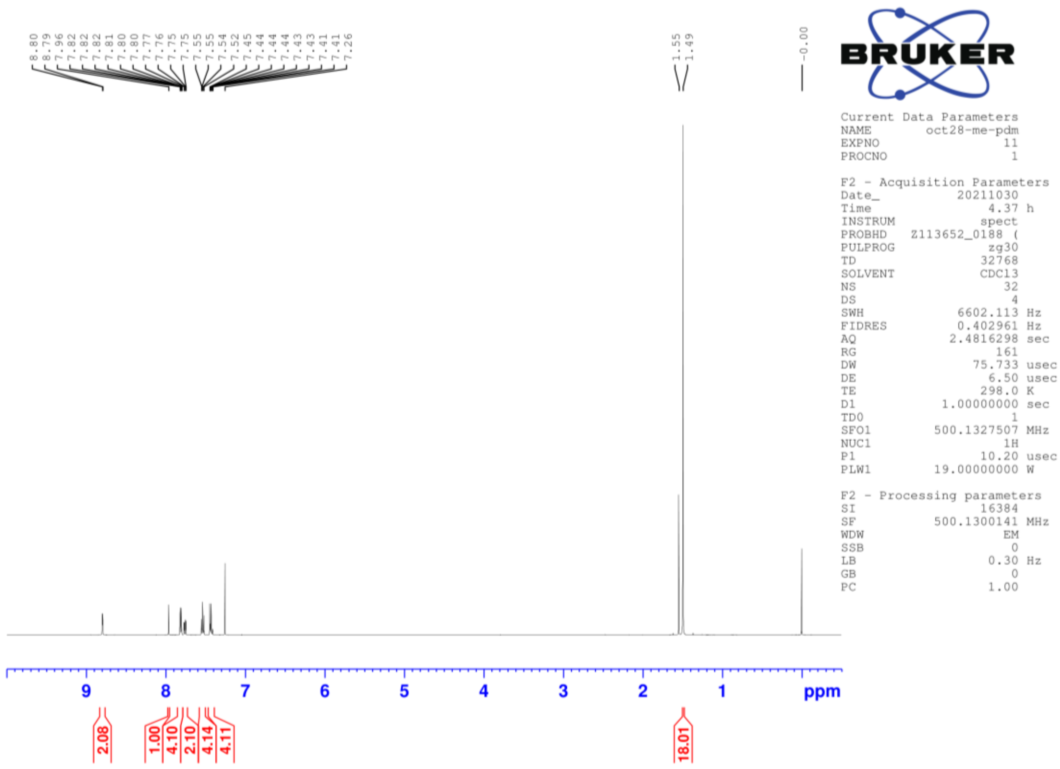

Supplementary Fig. S40 ^{1}H-NMR spectra of **mTDBA-2Ph.**

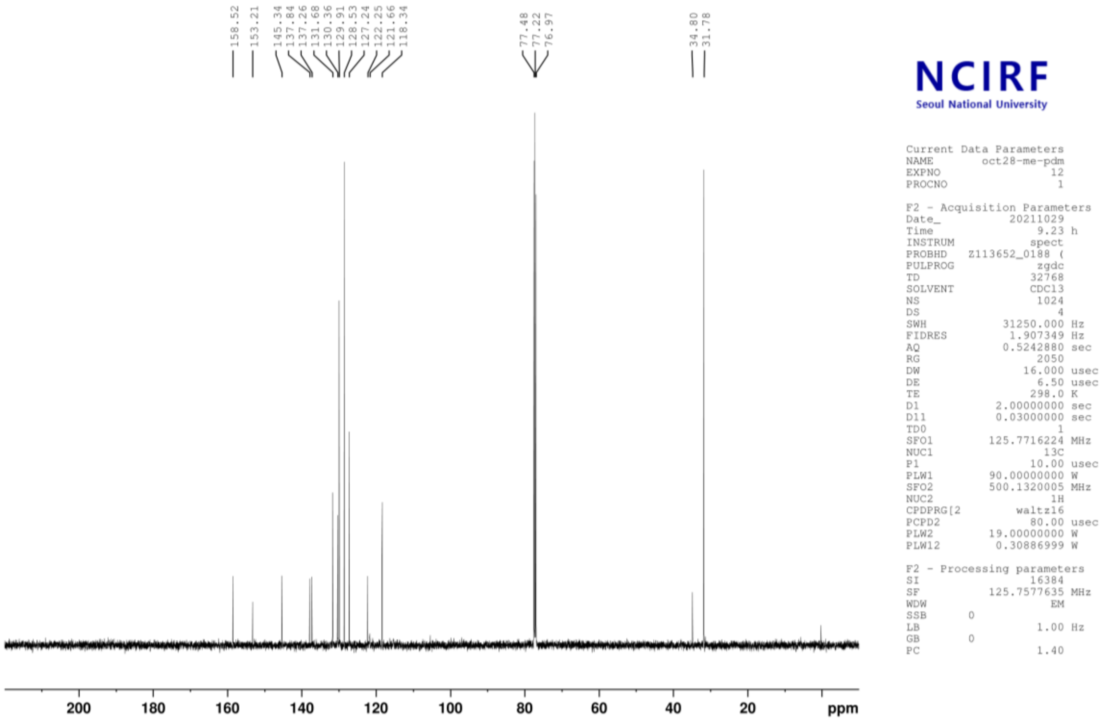

Supplementary Fig. S41 ^{13}C-NMR spectra of **mTDBA-2Ph.**

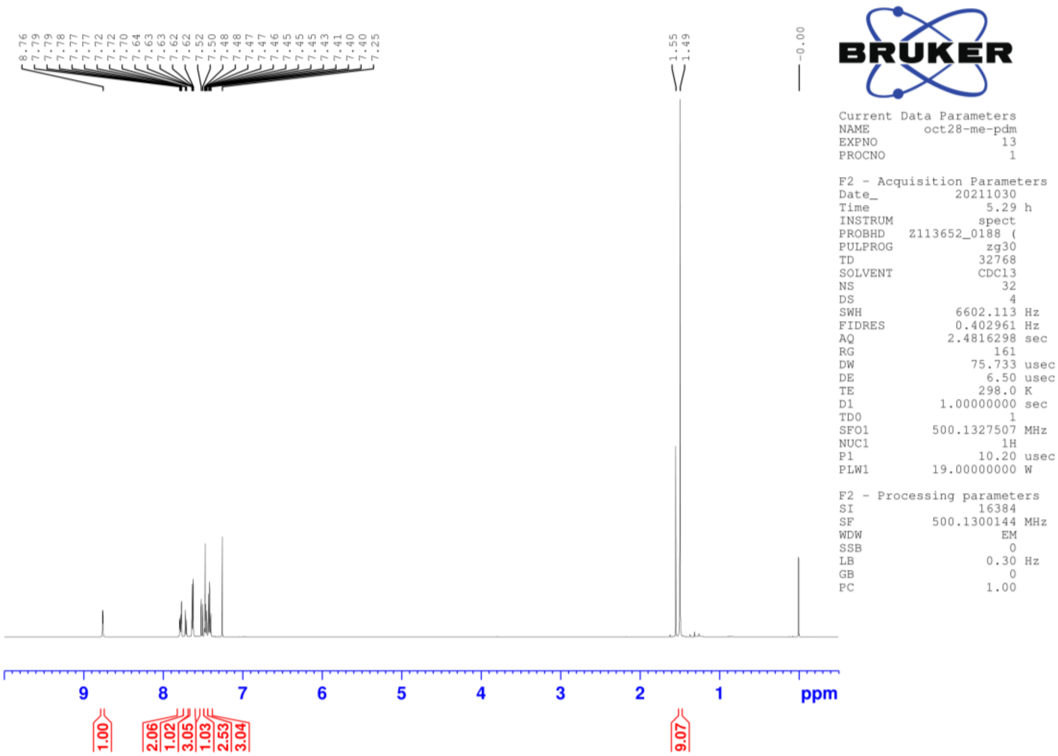

Supplementary Fig. S42 ^{1}H-NMR spectra of **TDBA-Si.**

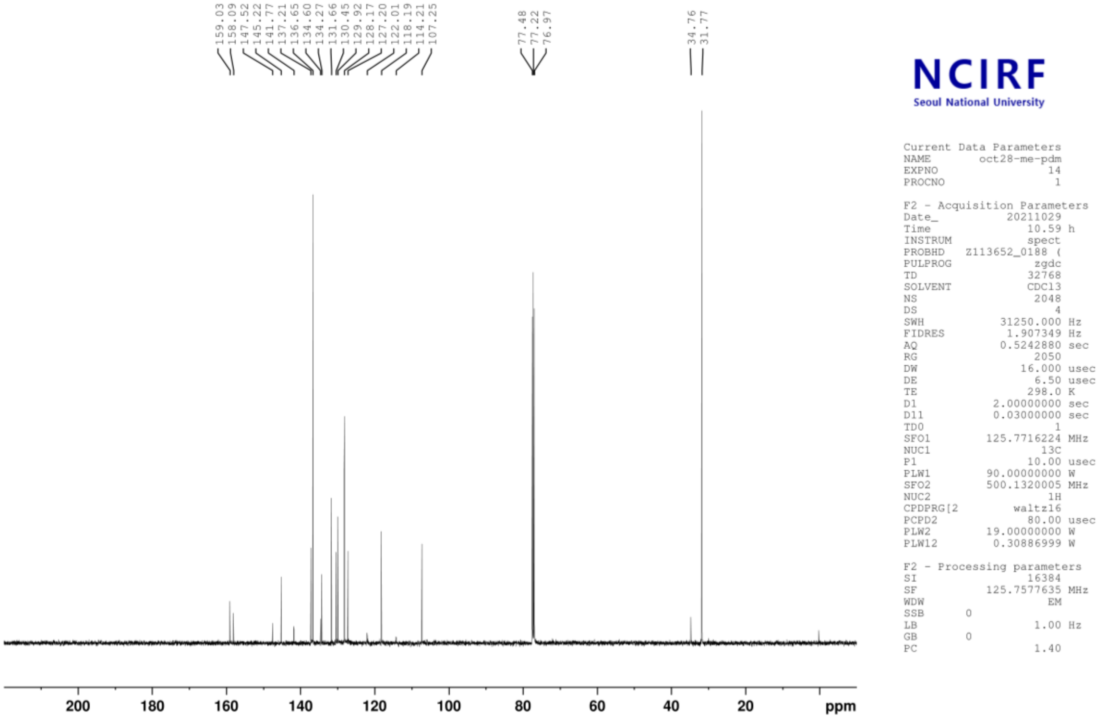

Supplementary Fig. S43 ^{13}C-NMR spectra of **TDBA-Si.**

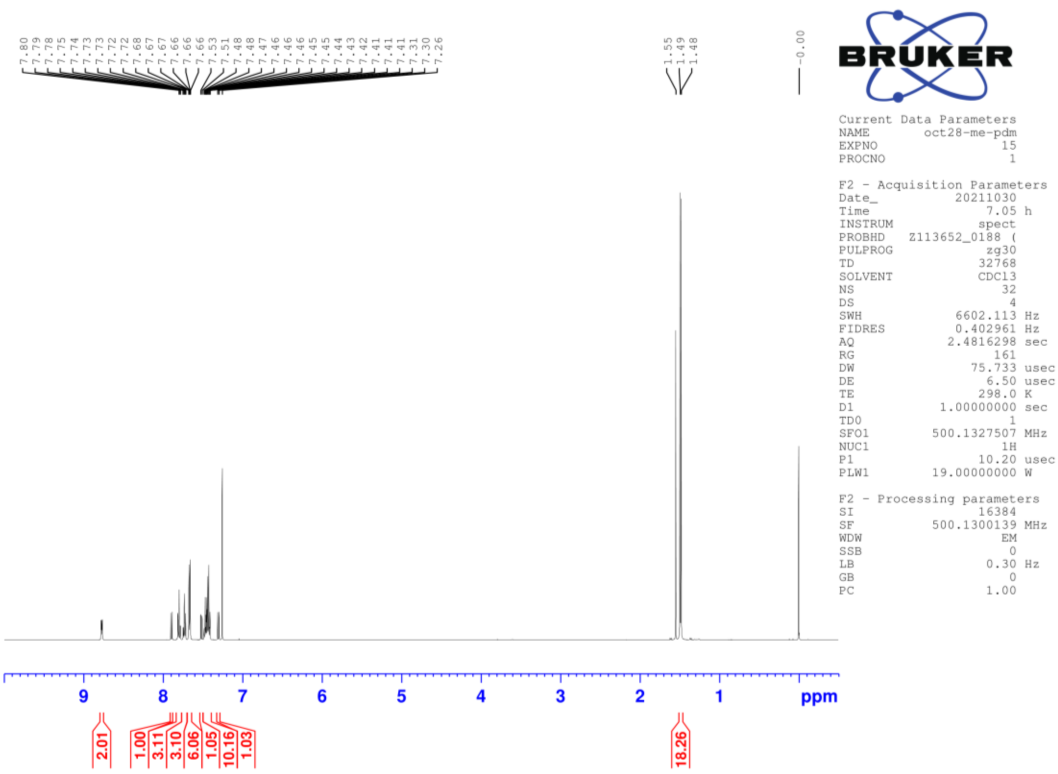

Supplementary Fig. S44 ^{1}H-NMR spectra of **mTDBA-Si.**

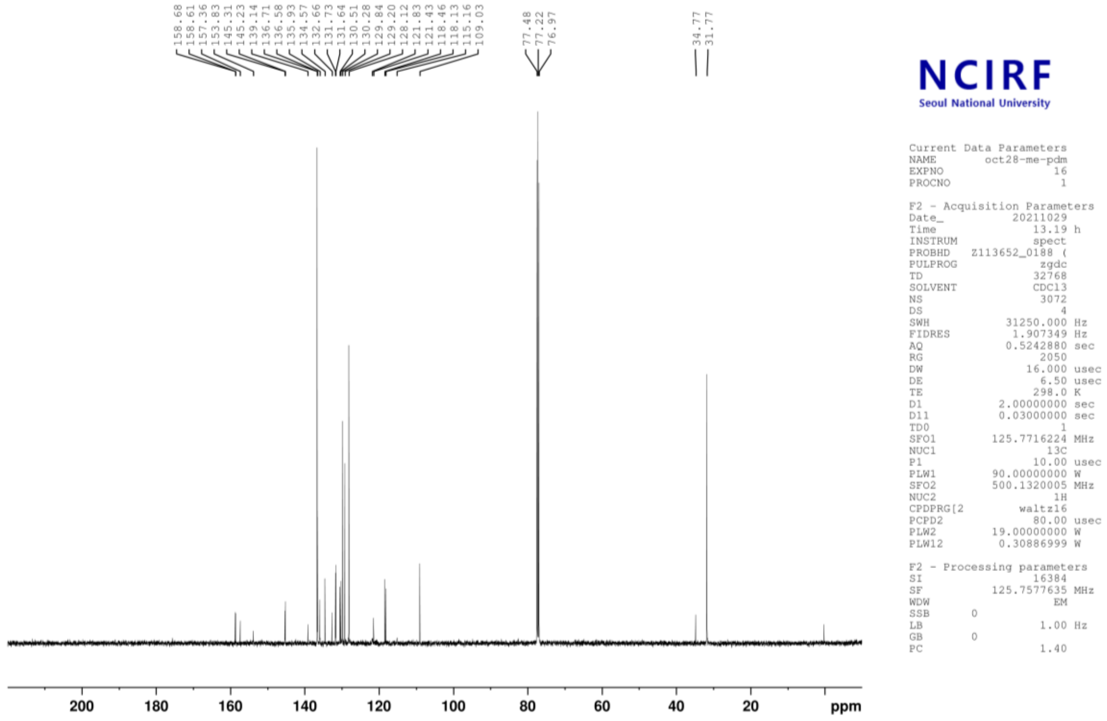

Supplementary Fig. S45 ^{13}C-NMR spectra of **mTDBA-Si.**

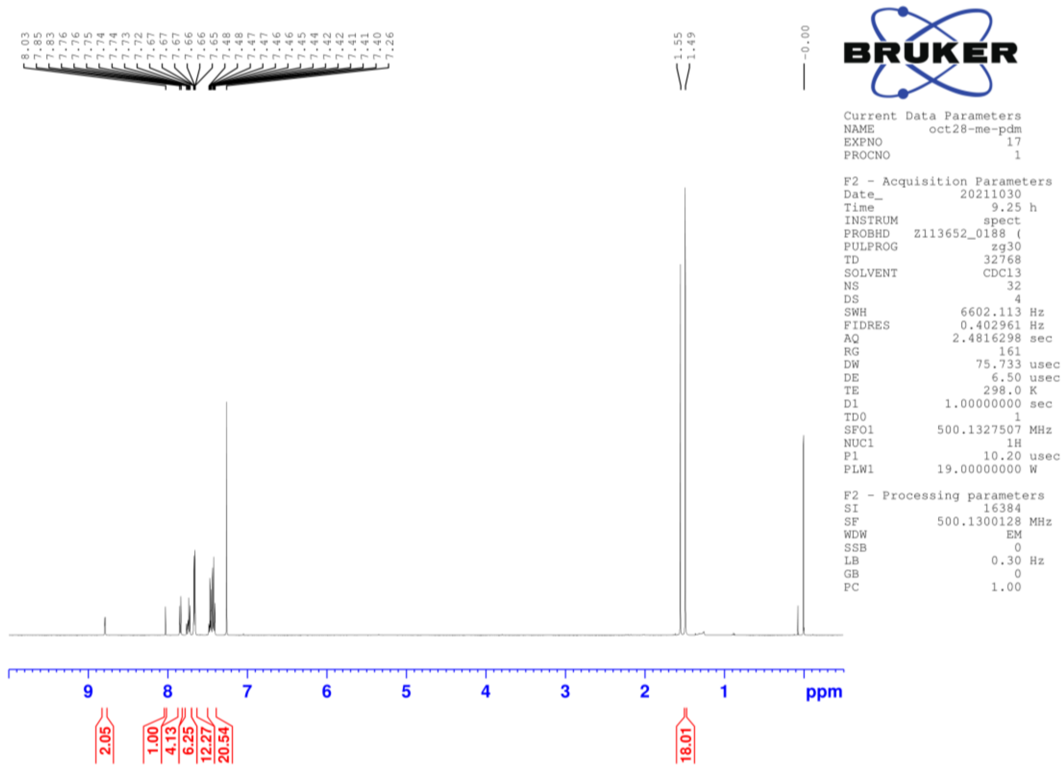

Supplementary Fig. S46 ^{1}H-NMR spectra of **mTDBA-2Si.**

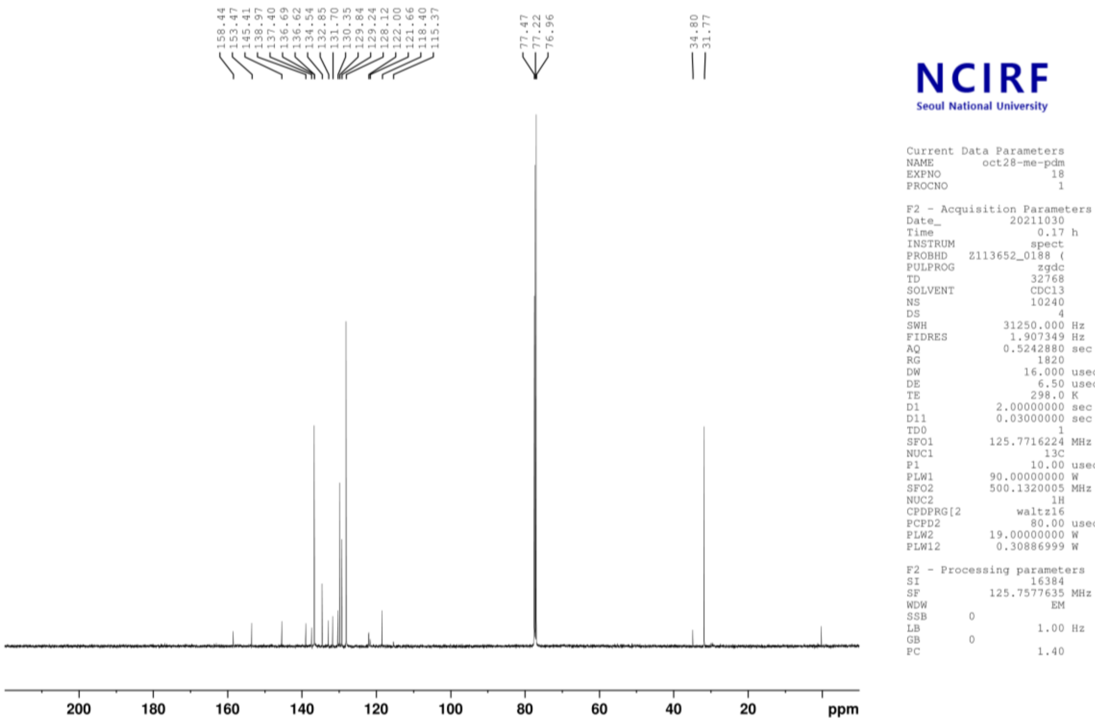

Supplementary Fig. S47 ^{13}C-NMR spectra of **mTDBA-2Si.**

Supplementary Table S1 Summary of TD-DFT calculation results of TDBA-based material calculated at the B3LYP/6-31G(d,p).

	HOMO (eV)	LUMO (eV)	$\Delta E_{HOMO \rightarrow LUMO}$ (eV)	S_1 (eV)	T_1 (eV)	ΔE_{ST}[a] (eV)	$f(S_0\text{-}S_1)$[b]
TDBA	-1.59	-5.49	3.90	3.37	2.85	0.52	0.1332
TDBA-Ph	-1.67	-5.49	3.83	3.32	2.80	0.52	0.1321
mTDBA-Ph	-1.61	-5.37	3.75	3.23	2.75	0.48	0.1047
mTDBA-2Ph	-1.63	-5.28	3.65	3.12	2.67	0.45	0.0986
TDBA-Si	-1.70	-5.50	3.80	3.30	2.78	0.52	0.1280
mTDBA-Si	-1.63	-5.38	3.76	3.23	2.76	0.48	0.1111
mTDBA-2Si	-1.65	-5.31	3.65	3.13	2.68	0.45	0.1188

[a] $\Delta E_{ST} = S_1 - T_1$. [b] Oscillator strength

Supplementary Table S2 Summary of Energy state TD-DFT calculation results of TDBA-based material calculated at the B3LYP/6-31G(d,p).

	T_1 (eV)	T_2 (eV)	S_1 (eV)
TDBA	2.85	3.31	3.37
TDBA-Ph	2.80	3.16	3.32
mTDBA-Ph	2.75	3.19	3.23
mTDBA-2Ph	2.67	3.06	3.12
TDBA-Si	2.78	3.11	3.30
mTDBA-Si	2.76	3.14	3.23
mTDBA-2Si	2.68	3.01	3.13

Supplementary Table S3 Summary of SOC values and representative electronic transition energies of TDBA-based materials.

	$\langle S_1 \vert \hat{H}_{SOC} \vert T_1 \rangle$ (cm^{-1})	$\langle S_1 \vert \hat{H}_{SOC} \vert T_2 \rangle$ (cm^{-1})
**TDBA**	0.01	0.17
**TDBA-Ph**	0.03	0.22
**mTDBA-Ph**	0.10	0.44
**mTDBA-2Ph**	0.13	0.31
**TDBA-Si**	0.03	0.13
**mTDBA-Si**	0.10	0.43
**mTDBA-2Si**	0.13	0.29

Supplementary Table S4 Rate constant for TDBA based host materials (non-doped film) at room temperature.

	**TDBA-Ph**	**mTDBA-Ph**	**mTDBA-2Ph**	**TDBA-Si**	**mTDBA-Si**	**mTDBA-2Si**
Φ	0.62	0.65	0.63	0.66	0.61	0.75
Φ_F	-	0.589	0.603	0.644	0.592	0.731
Φ_{TADF}	-	0.061	0.027	0.016	0.018	0.019
τ_f **(ns)**	9.35	10.01	8.92	7.80	11.1	6.50
τ_{TADF} **(μs)**	-	1.25	2.33	3.53	6.65	6.49
k_F **(×10^7)**	-	5.87	6.76	8.25	5.32	11.2
k_{IC}**(×10^7)**	-	3.16	3.97	4.25	3.40	3.75
k_{ISC}**(×10^7)**	-	9.27	4.85	3.14	2.63	3.92
Φ_{IC}	-	0.317	0.354	0.332	0.379	0.244
Φ_{ISC}	-	0.09	0.04	0.02	0.03	0.03
k_{TADF}**(×10^5)**	-	5.22	2.71	1.87	9.18	1.16
k_{RISC}**(×10^5)**	-	3.39	1.71	1.23	0.56	0.87

Supplementary Table S5 Fitting the decay curves triexponentially according to host materials in doped films.

Materials	τ_1 (μs)	τ_2 (μs)	τ_3 (μs)
**TDBA-Ph**	0.01	0.279	2.318
**mTDBA-Ph**	0.024	0.261	1.821
**mTDBA-2Ph**	0.015	0.228	1.250
**TDBA-Si**	0.010	0.257	1.05
**mTDBA-Si**	0.022	0.25	2.00
**mTDBA-2Si**	0.014	0.260	1.00

Supplementary Table S6 Rate constant for ν-DABNA in TDBA based host materials (2wt% doped) at room temperature.

	**TDBA-Ph**	**mTDBA-Ph**	**mTDBA-2Ph**	**TDBA-Si**	**mTDBA-Si**	**mTDBA-2Si**
Φ	0.82	0.93	0.86	0.82	0.94	0.92
Φ_F	0.742	0.859	0.782	0.735	0.834	0.789
Φ_{TADF}	0.078	0.071	0.078	0.085	0.106	0.131
τ_f **(ns)**	3.86	4.73	4.41	4.50	4.69	2.69
τ_{TADF} **(μs)**	3.00	2.76	1.80	0.96	2.25	0.98
k_F **(×10^8)**	1.92	1.82	1.77	1.63	1.78	2.94
k_{IC}**(×10^7)**	4.22	1.37	2.89	3.59	1.14	2.56
k_{ISC}**(×10^7)**	2.48	1.61	2.06	2.31	2.39	5.29
Φ_{IC}	0.163	0.065	0.127	0.161	0.053	0.069
Φ_{ISC}	0.10	0.08	0.09	0.10	0.11	0.14
k_{TADF}**(×10^5)**	2.73	3.37	4.78	8.54	4.18	9.39
k_{RISC}**(×10^5)**	2.25	3.13	3.98	7.01	4.58	8.64

Supplementary Table S7 Rate constant of energy transfer between host and dopant based on Stern-Volmer equation.

	TDBA-Ph	mTDBA-Ph	mTDBA-2Ph	TDBA-Si	mTDBA-Si	mTDBA-2Si
**Slope**	0.13	0.048	0.14	0.43	0.19	0.16
$\boldsymbol{k_q}^{a}$	3.43 x 10^7	1.02 x 10^7	3.23 x 10^7	9.48 x 10^7	4.08 x 10^7	5.90 x 10^7

[a] Energy transfer that occurs between host and dopant

Supplementary Table S8 FRET calculation summary between newly synthesized host materials and v-DABNA.

	PLQY	J (λ)[a] [mol^{-1}dm^{3}cm^{-1}nm^{4}]	R_F[b] [nm]	R[c] [nm]	k_{FRET}[d] [10^8/s]
**TDBA-Ph**	0.82	9.69 x 10^{14}	2.99	2.84	1.47
**mTDBA-Ph**	0.93	9.55 x 10^{14}	2.98	2.82	1.38
**mTDBA-2Ph**	0.86	1.00 x 10^{15}	3.01	2.93	1.32
**TDBA-Si**	0.82	1.02 x 10^{15}	3.02	3.11	1.07
**mTDBA-Si**	0.94	9.16 x 10^{14}	2.96	2.74	1.44
**mTDBA-2Si**	0.92	9.79 x 10^{14}	2.99	2.84	2.08

[a] Spectral overlap between PL emission of TDBA-based host materials and absorption spectrum of v-DABNA. [b] FRET radius. [c] Intermolecular distance. [d] FRET rate constant.

Supplementary Table S9 Charge mobility of TDBA-based host materials.

**Compounds**	**Mobility (cm^2/Vs) @ 1V**			
	Non-doped film		Doped film	
	HOD	EOD	HOD	EOD
**TDBA-Ph**	1.59×10^{-5}	1.01×10^{-6}	8.71×10^{-7}	7.79×10^{-8}
**mTDBA-Ph**	1.34×10^{-5}	9.61×10^{-7}	5.51×10^{-7}	6.51×10^{-8}
**mTDBA2Ph**	2.67×10^{-5}	8.89×10^{-7}	1.45×10^{-6}	6.96×10^{-8}
**TDBA-Si**	5.02×10^{-7}	7.04×10^{-7}	3.18×10^{-8}	3.04×10^{-8}
**mTDBA-Si**	2.83×10^{-7}	7.03×10^{-7}	2.39×10^{-8}	1.85×10^{-8}
**mTDBA-2Si**	9.40×10^{-7}	4.58×10^{-7}	5.63×10^{-8}	1.41×10^{-8}

Supplementary Table S10 Summary of the reported multiple resonance (MR) and donor-acceptor (DA) type OLEDs (CIE y < 0.15)

Dopant type	Host	Dopant	τ_d [a)] [μs]	EQE_{max} [b)] [%]	100nit [b)] [%]	1,000nit [b)] [%]	CIE y [c)]	λ [nm] [d)]	ref
	DOBNA-Tol	ν-DABNA-O-Me	7.7[e)]	29.5	28.8	26.9	0.1	465	1
	DOBNA-Tol	DABNA-NP-TB	90[e)]	19.5	17.5	12	0.11	457	2
	DOBNA-OAr	ν-DABNA	4.1[f)]	34.4	32.8	26	0.11	469	3
	DPEPO	B-O-DPA	224[g)]	16.3	6.5		0.05	443	4
	DBFPO	m-ν-DABNA	3.09 [h)]	36.2	-	-	0.12	471	5
	DBFPO	4F-ν-DABNA	3.12 [h)]	35.8	-	-	0.08	464	5
	DBFPO	4F-m-ν-DABNA	3.19 [h)]	33.7			0.06	461	5
	DBFPO	BN1	126.6 [i)]	31.2	18.3	9.3	0.08	457	6
	DBFPO	BN2	74.6 [i)]	33.2	25.5	15.5	0.11	467	6
	DBFPO	BN3	17.8 [i)]	37.6	34	26.2	0.08	458	6
	mCBP	BBCz-DB	86 [j)]	29.3	-	-	0.18	469	7
	mCBP	ν-DABNA	-	23	-	10	0.2	470	8
Multiple-resonance type	mCBP	DABNA-1	93.7 [k)]	13.5			0.09	464	9
	mCBP	DABNA-2	65.3 [k)]	20.2	13.4		0.13	468	9
	mCBP	BOBO-Z	7.7 [l)]	13.6	9.8	3.3	0.04	445	10
	mCBP	BOBS-Z	7.6 [l)]	26.9	24	15	0.06	456	10
	mCBP	BSBS-Z	6.7 [l)]	26.8	24	15.9	0.08	463	10
	mCBP	ν-DABNA	3.5 [l)]	24.6	21.2	14.9	0.12	472	10
	mCP:TSPO1	BisICz	-	6.5			0.04	437	11
	mCP:TSPO1	tBisICz	12.5 [m)]	15.1			0.05	445	11
	mCP:TSPO1	tPBisICz	1.74 [m)]	23.1			0.05	452	11
	mCBP/mCBP-CN	t-DAB-DPA	22.8 [n)]	27.6	21.8	9.2	0.08	459	12
	Polymer C	V-DABNA-Mes	2.39 [e)]	22.9	20.3	10.9	0.09	480	13
	SBON	v-DABNA	-	27.6	25.7	19.4	0.12	471	14
	SBON-Me	v-DABNA	-	22.8	19.3	13.3	0.12	471	14
	mCBP	v-DABNA	-	17.6	15.6	11.8	0.12	471	14
	DBFPO	TDBA-Ac	1.0 [o)]	21.5			0.06	458	15
	DBFPO	PXB-mIC	3.89 [p)]	12.5			0.08	450	16
	DPEPO	OBOtSAc	2.92 [q)]	31.2			0.092	452	17
	DPEPO	TDBA-SAF	1.34 [r)]	28.2			0.09	456	18
	DPEPO	CZ-TRZ3	13.0 [s)]	19.2			0.1	450	19
	DPEPO	CZ-TRZ4	10.3 [s)]	18.3			0.097	450	19
	DPEPO	ICzAc	9.86 [q)]	13.7			0.09	454	20
	DPEPO	CNICCz	6.46 [q)]	12.4	6.4		0.08	449	21
	DPEPO	CNICtCz	6.25 [q)]	16	10.7		0.13	456	21

	DPEPO	DtBuAc-DBT	136.4 [t)]	10.5	9.8		0.13	455	22
	DPEPO	DCzBN2	11.2 [q)]	7.7			0.07	417	23
	DPEPO	DCzBN3	13.5 [q)]	10.3			0.06	414	23
	DPEPO	DMACN-B	0.77 [r)]	10			0.04	444	24
	DPEPO	CzBPCN	48.22 [u)]	14			0.12	460	25
Donor-Acceptor type	DPEPO	CzOMeoB	52.0 [v)]	17.3	8.6		0.09	451	26
	DPEPO	CzMeoB	83.7 [v)]	16.1	12.4		0.1	455	26
	mCP	sAC-sDBB	134 [w)]	25.4	20		0.06	444	27
	mCP	sAC-DBB	106 [w)]	16.2	11.4		0.07	437	27
	mCP	TB-tCz	1.49 [w)]	15.9			0.06	412	28
	mCP	TB-tPCz	1.06 [w)]	14.1			0.05	420	28
	PPF	OBO-I	1.6 [x)]	21.7	20.6	16.2	0.1	457	29
	PPF	OBO-II	1.7 [x)]	31.7	29	20.8	0.13	464	29
	**TDBA-Si**	ν-DABNA	0.96	36.2	35.9	31.3	0.1	465	
This Work	**mTDBA-Si**	ν-DABNA	1.93	27.3	26.3	24.1	0.1	465	
	**mTDBA-2Si**	ν-DABNA	0.98	38.1	37.7	16.6	0.1	465	

[a)]Delayed lifetime calculated by photoluminescence decay. [b)]External quantum efficiency at maximum, 100nit, and 1000nit respectively. [c)]Commission Internationale de l'Eclairage coordinates from electroluminescence spectrum. [d)] Electroluminescence emission maximum. [e)]Obtained in 1wt% dopant in PMMA film [f)]Obtained in 1wt% dopant in DOBNA-OAr film [g)] Obtained in 10wt% dopant in DPEPO film. [h)] Obtained in 3wt% dopant in DBFPO film. [i)] Obtained in 1wt% dopant in DBFPO film. [j)] Obtained in 0.01mM toluene solution. [k)] Obtained in 1wt% dopant in mCBP film. [l)] Obtained in 3wt% dopant in mCBP film. [m)] Obtained in 1wt% dopant in mCP:TSPO1 film [n)] Obtained in 3wt% dopant in mCBP/mCBP-CN film. [p)] Obtained in 20wt% dopant in DBFPO film. [q)] Obtained in 10wt% dopant in DPEPO film. [r)] Obtained in 20wt% dopant in DPEPO film. [s)] Obtained in 6wt% dopant in DPEPO film. [t)] Obtained in 1wt% dopant in xeonex film. [u)] Obtained in 1wt% dopant in polystyrene film. [v)] Obtained in 0.02mM toluene solution. [w)] Obtained in 30wt% dopant in mCP film. [x)] Obtained in 20wt% dopant in PPF film.

Supplementary Methods

4,4'-((2-bromo-1,3-phenylene)bis(oxy))bis(*tert*-butylbenzene) (1). 2-bromo-1,3-difluorobenzene (10.00 g, 51.8 mmol), 4-*tert*-butylphenol (23.35 g, 155 mmol), and K_2CO_3 (21.48g, 155 mmol) were dissolved in *N*-methyl-2-pyrrolidone (120 mL) under argon saturated atmosphere. After stirring at 150 °C for 20 h, the reaction mixture was cooled to room temperature and the solution was poured into the water and extracted with dichloromethane three times. The organic phase was dried over anhydrous $MgSO_4$. The solution was condensed by evaporation under a vacuum and the crude product was purified through the silica gel column chromatography (CH_2Cl_2: *n*-hex = 1:9 (v/v)) to afford white solid (yield: 74%). ^{1}H NMR (500 MHz, $CDCl_3$) *δ.* 7.38-7.35 (d, *J* = 8.5 Hz, 4H), 7.13 – 7.10 (t, *J* = 8.5 Hz, 1H), 6.97 – 6.95 (d, *J* = 10.0 Hz, 4H), 6.67 – 6.65 (d, *J* = 10.0 Hz, 2H), 1.32 (s, 18H). ^{13}C NMR (126 MHz, $CDCl_3$) *δ.* 156.15, 154.41, 146.82, 128.34, 126.85, 118.39, 114.25, 34.56, 31.69. HRMS (FAB$^+$, *m*-NBA): calcd for $C_{26}H_{29}BrO_2$ ($[M+H]^+$), 452.1350, found:452.8425.

2,12-di-*tert*-butyl-5,9-dioxa-13b-boranaphtho[3,2,1-*de*]anthracene (TDBA). Compound 1 (2.01 g, 4.43 mmol) was dissolved in anhydrous *o*-xylene (30 mL) under an argon atmosphere. After stirring 15 min, *n*-butyllithium solution (3.32 mL, 1.6 M, 5.32 mmol) was slowly added to the solution at -

30 °C and stirred for 1 h at room temperature. After that, solution was cooled to -30 °C and followed by slow addition of boron tribromide (0.5 mL, 5.32 mmol). The solution was heated to room temperature and stirred for 1 h. Then, *N,N*-diisopropylethylamine (1.54 mL, 8.87 mmol) was slowly added at 0 °C. After further stirring at room temperature for 30 min, the mixture was heated at 140 °C for 20 h. Upon completion of the reaction, the solution was cooled to room temperature and extracted three times with dichloromethane and water. The organic phase was dried over $MgSO_4$ and filtrate was collected. After condensation of the solution by evaporation under a vacuum, the crude product was purified through the silica gel column chromatography (CH_2Cl_2: *n*-hex = 1:4 (v/v)) to afford white solid (yield: 15%). ^1H NMR (500 MHz, $CDCl_3$) *δ.* 8.76 – 8.75 (d, *J* = 5.0 Hz, 2H), 7.79 – 7.75 (m, 3H), 7.50 – 7.49 (d, *J* = 5.0 Hz, 2H), 7.21 – 7.19 (d, *J* = 10.0 Hz, 2H), 1.49 (s, 18H). ^{13}C NMR (126 MHz, $CDCl_3$) *δ.* 158.85, 157.75, 145.11, 134.53, 131.67, 130.44, 121.93, 118.19, 108.42, 34.76, 31.77. HRMS (FAB^+, *m*-NBA): calcd for $C_{26}H_{27}BO_2$ ($[M+H]^+$), 382.2104, found: 382.4420.

6-bromo-2,12-di-*tert*-butyl-5,9-dioxa-13b-boranaphtho[3,2,1-*de*]anthracene (mTDBA-Br). TDBA (0.5 g, 1.31 mmol) and *N*-bromosuccinimide (NBS) (0.27 g, 1.57 mmol) were dissolved in tetrahydrofuran (20 mL) under an argon atmosphere. The solution was stirred at room temperature for 8 h. After completion of the reaction, the solution was extracted with dichloromethane and water three times. The organic phase was dried over $MgSO_4$ and filtrate was collected. The solvent was condensed by evaporation under a vacuum and the crude product was purified by the silica gel column chromatography (*n*-hexane as eluent) to afford white solid (yield: 81%). ^1H NMR (500 MHz, $CDCl_3$) *δ.* 8.76 (t, *J* = 5.0 Hz, 2H), 7.96 – 7.95 (d, *J* = 5.0 Hz, 1H), 7.82 – 7.79 (m, 2H), 7.63 – 7.61 (d, *J* = 10.0 Hz, 1H), 7.51 – 7.49(d, *J* = 10 Hz, 1H), 7.15 – 7.14 (d, *J* = 5.0 Hz, 1H), 1.49 (s, 18H). ^{13}C NMR (126 MHz, $CDCl_3$) *δ.* 158.68, 158.66, 156.83, 153.45, 145.82, 145.47, 137.53, 132.03, 130.44, 130.34, 121.67, 118.49, 118.22, 110.13, 100.71, 34.81, 34.79, 31.74. HRMS (FAB^+, *m*-NBA): calcd for $C_{26}H_{26}BBrO_2$ ($[M+H]^+$), 460.1209, found:461.0514.

6,8-dibromo-2,12-di-*tert*-butyl-5,9-dioxa-13b-boranaphtho[3,2,1-*de*]anthracene (mTDBA-2Br). TDBA (1.32 g, 3.45 mmol) and NBS (1.48 g, 8.27 mmol) were dissolved in tetrahydrofuran (50 mL) under an argon atmosphere. The solution was stirred at room temperature for 8 h. After completion of the reaction, the solution was extracted with dichloromethane and water three times. The organic phase was dried over $MgSO_4$ and filtrate was collected. The solvent was condensed by evaporation under a vacuum and the crude product was purified by the silica gel column chromatography (*n*-hexane as eluent) to afford white solid (yield: 73%). ^1H NMR (500 MHz, $CDCl_3$) *δ*. 8.77-8.76 (d, *J* = 2.5 Hz, 2H), 8.12 (s, 1H), 7.84-7.82 (d, *J* = 2.5 Hz, 2H), 7.63 – 7.13 (d, *J* = 9.0 Hz, 2H), 1.49 (s, 18H). ^{13}C NMR (126 MHz, $CDCl_3$) *δ*. 158.50, 152.75, 146.16, 139.74, 132.39, 130.35, 118.51, 101.37, 31.71. HRMS (FAB^+, *m*-NBA): calcd for $C_{26}H_{25}BBr_2O_2$ ($[M+H]^+$), 538.0314, found:539.9722.

4,4'-((2,5-dibromo-1,3-phenylene)bis(oxy))bis(tert-butylbenzene) (2) Synthesis of this compounds were previously described We followed the same synthetic conditions and procedure to prepare this compounds. Please note the references for the synthetic details[15].

7-bromo-2,12-di-*tert*-butyl-5,9-dioxa-13b-boranaphtho[3,2,1-*de*]anthracene (TDBA-Br) Synthesis of this compounds were previously described. We followed the same synthetic conditions and procedure to prepare this compounds. Please note the references for the synthetic details[30].

2,12-di-*tert*-butyl-7-phenyl-5,9-dioxa-13b-boranaphtho[3,2,1-*de*]anthracene (TDBA-Ph). TDBA-Br (0.70 g, 1.52 mmol), phenylboronic acid (0.28 g, 2.28 mmol), K_2CO_3 (0.70 g, 5.06 mmol), and tetrakis(triphenylphosphine)palladium(0) (0.18 g, 0.15 mmol) were dissolved in 10 mL of toluene and 5 mL of distilled water at room temperature under an argon atmosphere. This mixture was refluxed until the reaction is completed. After completion of the reaction, the mixture was cooled to room temperature and the solution was extracted with dichloromethane and water three times. The organic phase was dried over $MgSO_4$ and filtrate was collected. The solvent was condensed by evaporation under a vacuum and the crude product was purified by the silica gel column chromatography (CH_2Cl_2:*n*-hex = 1:4 (v/v)) to afford white solid (yield: 66%). ^{1}H NMR (500 MHz, $CDCl_3$) *δ*. 8.76 (d, *J* = 2.5 Hz, 2H), 7.92 – 7.75 (m, 4H), 7.52 – 7.49 (m, 4H), 7.45 – 7.41 (m, 3H), 1.49 (s, 18H). ^{13}C NMR (126 MHz, $CDCl_3$) *δ*. 159.05, 158.06, 147.85, 145.18, 140.79, 131.63, 130.45, 129.15, 128.46, 127.83, 122.01, 118.19, 114.01, 107.25, 34.76, 31.77. HRMS (FAB$^+$, *m*-NBA): calcd for $C_{32}H_{31}BO_2$ ([M+H]$^+$), 458.2417, found: 458.5537.

2,12-di-*tert*-butyl-6-phenyl-5,9-dioxa-13b-boranaphtho[3,2,1-*de*]anthracene (mTDBA-Ph). mTDBA-Ph was prepared following the same procedure used for the synthesis of TDBA-Ph, except using mTDBA-Br in place of TDBA-Br. The crude products was purified through the silica gel column chromatography (CH_2Cl_2:*n*-hex = 1:4 (v/v)) to afford white solid (yield: 72%). ^{1}H NMR (500 MHz, $CDCl_3$) *δ*. 8.78 (dd, *J* = 2.5 Hz, 2H), 7.87 – 7.85 (d, *J* = 8.5 Hz, 1H), 7.80 – 7.73 (m, 4H), 7.54 – 7.51 (m, 3H), 7.43 – 7.40 (m, 2H), 7.31 – 7.29 (d, *J* = 8.0 Hz, 1H), 1.50 – 1.48 (d, *J* = 7.5 Hz 18H). ^{13}C NMR (126 MHz, $CDCl_3$) *δ*. 158.71, 158.65, 157.20, 153.71, 145.26, 145.19, 138.00, 135.90, 131.72, 131.63, 130.50, 130.29, 129.85, 128.49, 127.12, 121.81, 121.75, 118.40, 118.14, 115.12, 108.89, 34.77, 31.78. HRMS (FAB$^+$, *m*-NBA): calcd for $C_{32}H_{31}BO_2$ ([M+H]$^+$), 458.2417, found: 458.4464.

2,12-di-*tert*-butyl-6,8-diphenyl-5,9-dioxa-13b-boranaphtho[3,2,1-*de*]anthracene (mTDBA-2Ph). mTDBA-2Ph was prepared following the same procedure used for the synthesis of TDBA-Ph, except using mTDBA-2Br in place of TDBA-Br. The crude products was purified through the silica gel column chromatography (CH_2Cl_2:*n*-hex = 1:4 (v/v)) to afford yellow solid (yield: 73%). ^{1}H NMR (500 MHz, $CDCl_3$) *δ.* 8.80 (d, *J* = 2.5 Hz, 2H), 7.97 (s, 1H), 7.82 – 7.80 (m, 4H), 7.77 – 7.75 (dd, *J* = 2.5 Hz, 2H), 7.54 (t, *J* = 7.5 Hz, 4H), 7.45 – 7.41 (m, 4H), 1.49 (s, 18H). ^{13}C NMR (126 MHz, $CDCl_3$) *δ*. 158.52, 153.21, 145.34, 137.84, 137.26, 131.68, 130.36, 129.91, 128.53, 127.24, 122.25, 121.66, 118.34, 34.80, 31.78. HRMS (FAB$^+$, *m*-NBA): calcd for $C_{38}H_{35}BO_2$ ([M+H]$^+$), 534.2730, found: 534.4803.

4-bromo-triphenylsilylbenzene(3). Synthesis of this compounds were previously described We followed the same synthetic conditions and procedure to prepare this compounds. Please note the references for the synthetic details[30].

4-triphenylsilylphenylboronic acid(4). Synthesis of this compounds were previously described We followed the same synthetic conditions and procedure to prepare this compounds. Please note the references for the synthetic details[30].

(4-(2,12-di-*tert*-butyl-5,9-dioxa-13b-boranaphtho[3,2,1-*de*]anthracen-7-yl)phenyl) triphenylsilane (TDBA-Si). (4-(triphenylsilyl)phenyl)boronic acid (0.79 g, 2.08 mmol), TDBA-Br (0.80 g, 1.73 mmol), K_2CO_3 (2.21 g, 20.00 mmol), and $Pd(PPh_3)_4$ (0.20 g, 0.17 mmol) were dissolved in 20 mL of toluene and 10 mL of distilled water at room temperature under an argon atmosphere. This mixture was refluxed until the reaction is completed. After completion of the reaction, the mixture was cooled to room temperature and the solution was extracted with dichloromethane and water three times. The organic phase was dried over $MgSO_4$ and filtrate was collected. The solvent was condensed by evaporation under a vacuum and the crude product was purified by the silica gel column chromatography (CH_2Cl_2:*n*-hex = 1:4 (v/v)) to afford white solid (yield: 66%). ^{1}H NMR (500 MHz, $CDCl_3$) *δ*. 8.76 (d, *J* = 2.5 Hz, 2H), 7.79 – 7.77 (m, 4H), 7.72 – 7.70 (d, *J* = 8.5 Hz, 2H), 7.64 – 7.62 (m, 6H), 7.52 – 7.50 (d, *J* = 8.5 Hz, 2H), 7.48 – 7.41 (m, 5H), 7.40 (m, 6H) 1.49 (s, 18H). ^{13}C NMR (126 MHz, $CDCl_3$) *δ*. 159.03, 158.09, 147.52, 145.22, 141.77, 137.21, 136.65, 134.60, 134.27, 131.66, 130.45, 129.92, 128.17, 127.20, 122.01, 118.19, 114.21, 107.25, 34.76, 31.77. HRMS (FAB$^+$, *m*-NBA): calcd for $C_{50}H_{45}BO_2Si$ ([M+H]$^+$), 716.3282, found: 716.5239.

(4-(2,12-di-*tert*-butyl-5,9-dioxa-13b-boranaphtho[3,2,1-*de*]anthracen-6-yl)phenyl) triphenylsilane (mTDBA-Si). mTDBA-Si was prepared following the same procedure used for the synthesis of TDBA-Si, except using mTDBA-Br in place of TDBA-Br. The crude products was purified through the silica gel column chromatography (CH_2Cl_2:*n*-hex = 1:4 (v/v)) to afford yellow solid (yield: 72%). ^{1}H NMR (500 MHz, $CDCl_3$) *δ*. 8.78 (dd, *J* = 2.5 Hz, 2H), 7.90 (d, *J* = 8.5 Hz, 1H), 7.81 – 7.78 (m, 3H), 7.75 – 7.72 (m, 3H), 7.68 – 7.66 (m, 6H), 7.53 – 7.51 (d, *J* = 8.5 Hz, 1H), 7.47 – 7.41 (m, 10H), 7.31 – 7.30 (d, *J* = 5.0 Hz, 1H),1.50 – 1.48(d, *J* = 7.0 Hz 18H). ^{13}C NMR (126 MHz, $CDCl_3$) *δ*.158.68, 158.61, 157.36, 153.83, 145.31, 145.23, 139.14, 136.71, 136.58, 135.93, 134.57, 132.66, 131.73, 131.64, 130.51, 130.28, 129.84, 129.20, 128.12, 121.83, 121.43, 118.46, 118.13, 115.16, 109.03, 34.77, 31.77. HRMS (FAB$^+$, *m*-NBA): calcd for $C_{38}H_{35}BO_2$ ([M+H]$^+$), 716.3282, found: 716.4137.

((2,12-di-*tert*-butyl-5,9-dioxa-13b-boranaphtho[3,2,1-*de*]anthracene-6,8-diyl)bis(4,1-phenylene))bis(triphenylsilane) (mTDBA-2Si). mTDBA-2Si was prepared following the same procedure used for the synthesis of TDBA-Si, except using mTDBA-2Br in place of TDBA-Br. The crude products was purified through the silica gel column chromatography (CH_2Cl_2:*n*-hex = 1:4 (v/v)) to afford yellow solid (yield: 73%). ^{1}H NMR (500 MHz, $CDCl_3$) *δ*. 8.79 (d, *J* = 2.5 Hz, 2H), 8.03 (s, 1H), 7.85 – 7.83 (d, *J* = 8.0 Hz, 4H), 7.76 – 7.72 (m, 6H), 7.67 – 7.65 (m, 12H), 7.48 – 7.40 (m, 20H), 1.49(s, 18H). ^{13}C NMR (126 MHz, $CDCl_3$) *δ*. 158.44, 153.47, 145.41, 138.97, 137.40, 136.69, 136.62, 134.54, 132.85, 131.70, 130.35, 129.84, 129.24, 128.12, 122.00, 121.66, 118.40, 115.37, 34.80, 31.77. HRMS (FAB$^+$, *m*-NBA): calcd for $C_{74}H_{63}BO_2Si_2$ ([M+H]$^+$), 1050.4460, found: 1050.7910.

Supplementary References

1. Tanaka, H. et al. Hypsochromic Shift of Multiple-Resonance-Induced Thermally Activated Delayed Fluorescence by Oxygen Atom Incorporation. *Angew. Chem., Int. Ed.* **60**, 17910-17914 (2021).

2. Oda, S. et al. Carbazole-Based DABNA Analogues as Highly Efficient Thermally Activated Delayed Fluorescence Materials for Narrowband Organic Light-Emitting Diodes. *Angew. Chem., Int. Ed.* **133**, 2918-2922 (2021).

3. Kondo, Y. et al. Narrowband deep-blue organic light-emitting diode featuring an organoboron-based emitter. *Nat. Photon.* **13**, 678-682 (2019).

4. Park, J. et al. Asymmetric blue multiresonance TADF emitters with a narrow emission band. *ACS Appl. Mater. Interfaces* **13**, 45798-45805 (2021).

5. Naveen, K. R. et al. Deep blue diboron embedded multi-resonance thermally activated delayed fluorescence emitters for narrowband organic light emitting diodes. *Chem. Eng. J.* **432**, 134381 (2022).

6. Lv, X. et al. Extending the π-Skeleton of Multi-Resonance TADF Materials towards High-Efficiency Narrowband Deep-Blue Emission. *Angew. Chem., Int. Ed.* **61**, e20220158 (2022).

7. Yang, M., Park, I. S. & Yasuda, T. Full-color, narrowband, and high-efficiency electroluminescence from boron and carbazole embedded polycyclic heteroaromatics. *J. Am. Chem. Soc.* **142**, 19468-19472 (2020).

8. Chan, C. et al. Stable pure-blue hyperfluorescence organic light-emitting diodes with high-efficiency and narrow emission. *Nat. Photon.* **15**, 203-207 (2021).

9. Hatakeyama, T. et al. Ultrapure blue thermally activated delayed fluorescence molecules: efficient HOMO–LUMO separation by the multiple resonance effect. *Adv. Mater.* **28**, 2777-2781 (2016).

10. Park, I. S., Yang, M., Shibata, H., Amanokura, N. & Yasuda, T. Achieving Ultimate Narrowband and Ultrapure Blue Organic Light-Emitting Diodes Based on Polycyclo-Heteraborin Multi-Resonance Delayed-Fluorescence Emitters. *Adv. Mater.* **34**, 2107951 (2022).

11. Patil, V. V. et al. Purely Spin-Vibronic Coupling Assisted Triplet to Singlet Up-Conversion for Real Deep Blue Organic Light-Emitting Diodes with Over 20% Efficiency and y Color Coordinate of 0.05. *Adv. Sci.* **8**, 2101137 (2021).

12. Kim, J. H., Chung, W. J., Kim, J. & Lee, J. Y. Concentration quenching–resistant multiresonance thermally activated delayed fluorescence emitters. *Mater. Today Energy.* **21**, 100792 (2021).

13. Oda, Susumu, et al. One-shot synthesis of expanded heterohelicene exhibiting narrowband thermally activated delayed fluorescence. *J. Am. Chem. Soc.* **144**, 106-112 (2021).

14. Hayakawa, Masahiro, et al. "Spiroborate-Based Host Materials with High Triplet Energies and Ambipolar Charge-Transport Properties." *Angew. Chem., Int. Ed.* **62***,* e202217512 (2023).

15. Ahn, D. H. et al. Highly efficient blue thermally activated delayed fluorescence emitters based on symmetrical and rigid oxygen-bridged boron acceptors. *Nat. Photon.* **13**, 540-546 (2019).

16. Ahn, D. H. et al. Highly twisted donor–acceptor boron emitter and high triplet host material for highly efficient blue thermally activated delayed fluorescent device. *ACS Appl. Mater. Interfaces* **11**, 14909-14916 (2019).

17. Lee, Y. & Hong, J. High-Efficiency Thermally Activated Delayed Fluorescence Emitters with High Horizontal Orientation and Narrow Deep-Blue Emission. *Adv. Opt. Mater.* **9**, 2100406 (2021).

18. Lim, H. et al. Highly Efficient Deep-Blue OLEDs using a TADF Emitter with a Narrow Emission Spectrum and High Horizontal Emitting Dipole Ratio. *Adv. Mater.* **32**, 2004083 (2020).

19. Cui, L. et al. Controlling singlet–triplet energy splitting for deep-blue thermally activated delayed fluorescence emitters. *Angew. Chem.* **56**, 1571-1575 (2017).

20. Seo, J., Im, Y., Han, S. H., Lee, C. W. & Lee, J. Y. Unconventional molecular design approach of high-efficiency deep blue thermally activated delayed fluorescent emitters using indolocarbazole as an acceptor. *ACS Appl. Mater. Interfaces* **9**, 37864-37872 (2017).

21. Im, Y., Han, S. H. & Lee, J. Y. Deep blue thermally activated delayed fluorescent emitters using CN-modified indolocarbazole as an acceptor and carbazole-derived donors. *J. Mater. Chem. C* **6**, 5012-5017 (2018).

22. Huang, R. et al. Balancing charge-transfer strength and triplet states for deep-blue thermally activated delayed fluorescence with an unconventional electron rich dibenzothiophene acceptor. *J. Mater. Chem. C* **7**, 13224-13234 (2019).

23. Chan, C., Cui, L., Kim, J. U., Nakanotani, H. & Adachi, C. Rational molecular design for deep-blue thermally activated delayed fluorescence emitters. *Adv. Funct. Mater.* **28**, 1706023 (2018).

24. Khan, A. et al. Intramolecular-Locked High Efficiency Ultrapure Violet-Blue (CIE-y< 0.046) Thermally Activated Delayed Fluorescence Emitters Exhibiting Amplified Spontaneous Emission. *Adv. Funct. Mater.* **31**, 2009488 (2021).

25. Cho, Y.J., Jeon, S.K., Lee, S.-S., Yu, E. & Lee, J.Y. Donor Interlocked Molecular Design for Fluorescence-like Narrow Emission in Deep Blue Thermally Activated Delayed Fluorescent Emitters. *Chem. Mater.* **28**, 5400-5405 (2016).

26. Lee, Y. H. et al. High-Efficiency Sky Blue to Ultradeep Blue Thermally Activated Delayed Fluorescent Diodes Based on Ortho-Carbazole-Appended Triarylboron Emitters: Above 32% External Quantum Efficiency in Blue Devices. *Adv. Opt. Mater.* **6**, 1800385 (2018).

27. Xia, G. et al. A TADF Emitter Featuring Linearly Arranged Spiro-Donor and Spiro-Acceptor Groups: Efficient Nondoped and Doped Deep-Blue OLEDs with CIEy< 0.1. *Angew. Chem., Int. Ed.* **133**, 9684-9689 (2021).

28. Kim, H. J. et al. Ultra-Deep-Blue Aggregation-Induced Delayed Fluorescence Emitters: Achieving Nearly 16% EQE in Solution-Processed Nondoped and Doped OLEDs with CIEy< 0.1. *Adv. Funct. Mater*. **31**, 2102588 (2021).

29. Park, I. S., Min, H., Kim, J. U. & Yasuda, T. Deep-blue OLEDs based on organoboron–phenazasiline-hybrid delayed fluorescence emitters concurrently achieving 30% external quantum efficiency and small efficiency roll-off. *Adv. Opt. Mater.* **9**, 2101282 (2021).

30. You, Y., An, C.-G., Lee, D.-S., Kim, J.-J. & Park, S.Y. Silicon-containing dendritic tris-cyclometalated Ir(iii) complex and its electrophosphorescence in a polymer host. *J. Mater. Chem*. **16**, 4706-4713 (2006).
