# Supplementary material for: High-performance blue OLED using multiresonance thermally activated delayed fluorescence host materials containing silicon atoms
Source: Nat Commun. 2023 Sep 11;14:5589. doi: 10.1038/s41467-023-41440-1 (PMC10495399; doi:10.1038/s41467-023-41440-1)
Supplement: Supplementary file 3 — Description of Additional Supplementary Files [file 41467_2023_41440_MOESM3_ESM.pdf]

Description of Additional Supplementary Files

File name: Supplementary Data 1

Description: ORTEP of TDBA-Ph.

File name: Supplementary Data 2

Description: ORTEP of mTDBA-Ph.

File name: Supplementary Data 3

Description: ORTEP of TDBA-Si.

File name: Supplementary Data 4

Description: ORTEP of mTDBA-Si.

File name: Supplementary Data 5

Description: CheckCIF of TDBA-Ph.

File name: Supplementary Data 6

Description: CheckCIF of mTDBA-Ph.

File name: Supplementary Data 7

Description: CheckCIF of TDBA-Si.

File name: Supplementary Data 8

Description: CheckCIF of mTDBA-Si.

File name: Supplementary Data 9

Description: TD-DFT calcualtion of TDBA

File name: Supplementary Data 10

Description: TD-DFT calcualtion of TDBA-Ph.

File name: Supplementary Data 11

Description: TD-DFT calcualtion of mTDBA-Ph.

File name: Supplementary Data 12

Description: TD-DFT calcualtion of mTDBA-2Ph.

File name: Supplementary Data 13

Description: TD-DFT calcualtion of TDBA-Si.

File name: Supplementary Data 14

Description: TD-DFT calcualtion of mTDBA-Si.

File name: Supplementary Data 15

Description: TD-DFT calcualtion of mTDBA-2Si.

File name: Supplementary Data 16

Description: Spin-orbital coupling (SOC) calcualtion of TDBA.

File name: Supplementary Data 17

Description: Spin-orbital coupling (SOC) calcualtion of TDBA-Ph.

File name: Supplementary Data 18

Description: Spin-orbital coupling (SOC) calcualtion of mTDBA-Ph.

File name: Supplementary Data 19

Description: Spin-orbital coupling (SOC) calcualtion of mTDBA-2Ph.

File name: Supplementary Data 20

Description: Spin-orbital coupling (SOC) calcualtion of TDBA-Si.

File name: Supplementary Data 21

Description: Spin-orbital coupling (SOC) calcualtion of mTDBA-Si.

File name: Supplementary Data 22

Description: Spin-orbital coupling (SOC) calcualtion of mTDBA-2Si.
