# Supplementary figures and images for: High-performance blue OLED using multiresonance thermally activated delayed fluorescence host materials containing silicon atoms
Source: Nat Commun. 2023 Sep 11;14:5589. doi: 10.1038/s41467-023-41440-1 (PMC10495399; doi:10.1038/s41467-023-41440-1)

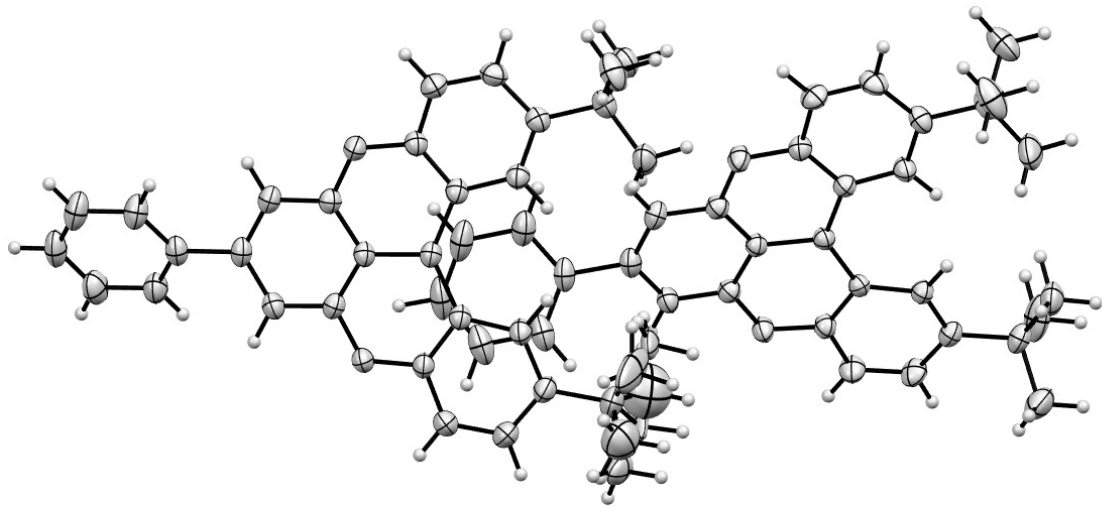

Supplement: Supplementary file 4 — Supplementary Data 1 [file 41467_2023_41440_MOESM4_ESM.pdf]

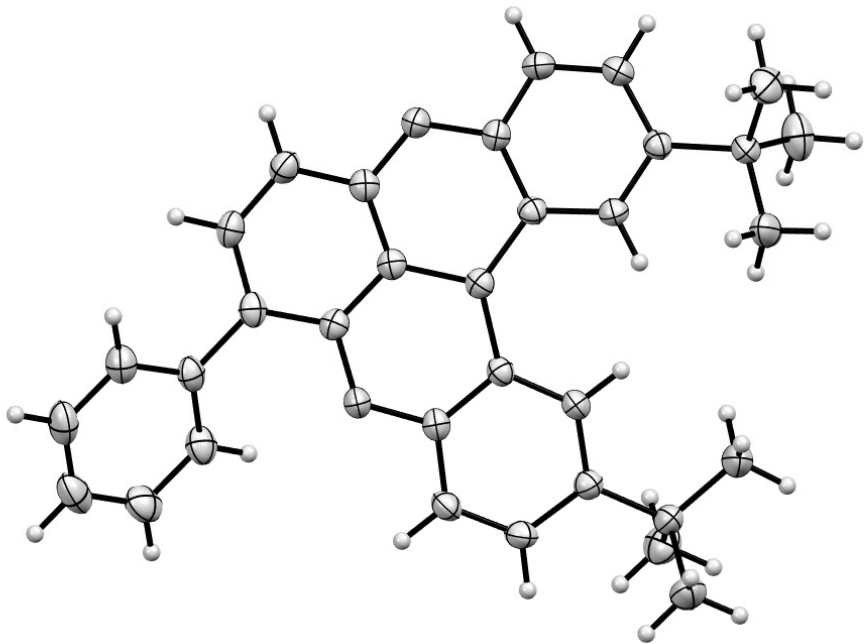

Supplement: Supplementary file 5 — Supplementary Data 2 [file 41467_2023_41440_MOESM5_ESM.pdf]

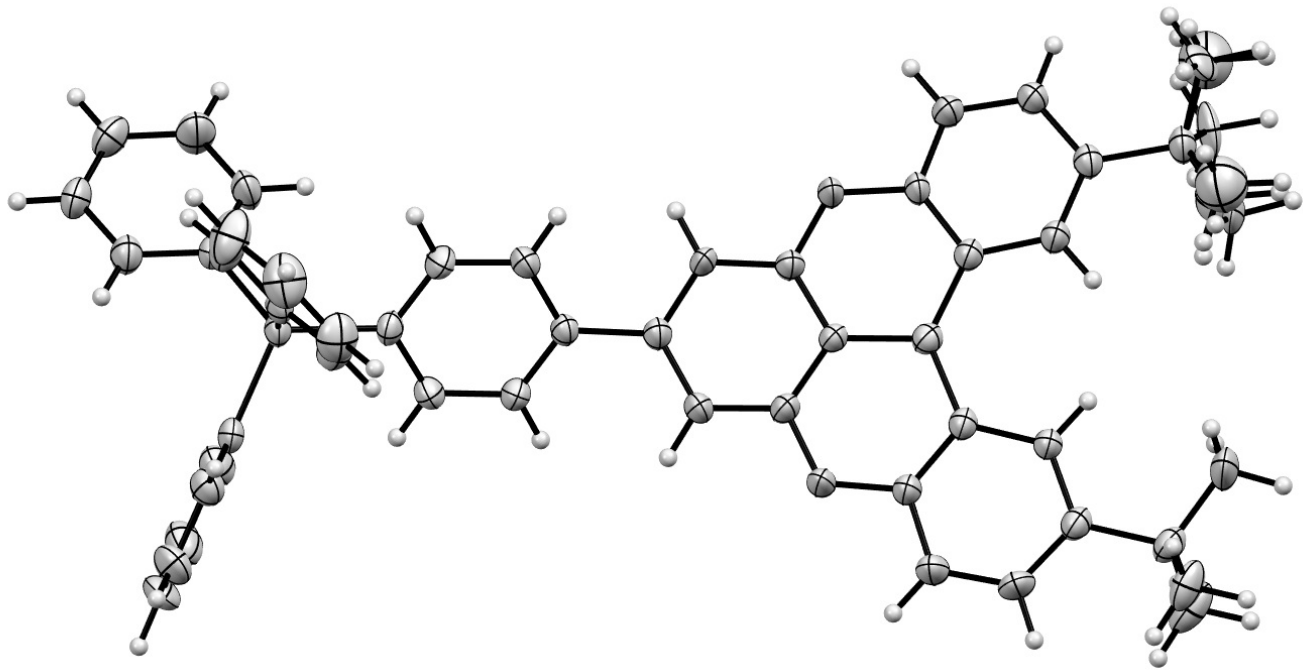

Supplement: Supplementary file 6 — Supplementary Data 3 [file 41467_2023_41440_MOESM6_ESM.pdf]

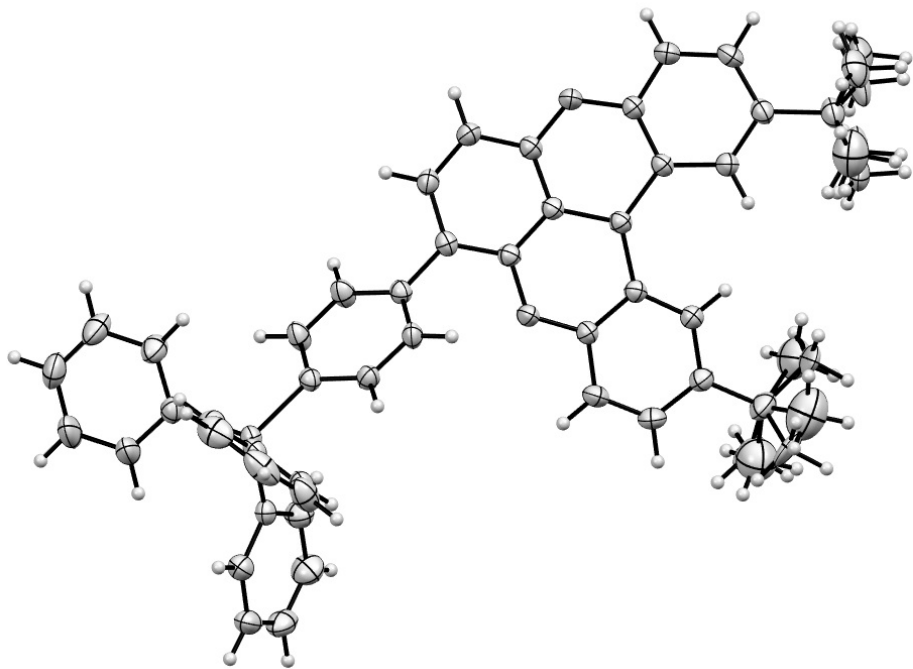

Supplement: Supplementary file 7 — Supplementary Data 4 [file 41467_2023_41440_MOESM7_ESM.pdf]
