# Supplementary material for: High-performance blue OLED using multiresonance thermally activated delayed fluorescence host materials containing silicon atoms
Source: Nat Commun. 2023 Sep 11;14:5589. doi: 10.1038/s41467-023-41440-1 (PMC10495399; doi:10.1038/s41467-023-41440-1)

checkCIF/PLATON report

Structure factors have been supplied for datablock(s) 3_a

THIS REPORT IS FOR GUIDANCE ONLY. IF USED AS PART OF A REVIEW PROCEDURE FOR PUBLICATION, IT SHOULD NOT REPLACE THE EXPERTISE OF AN EXPERIENCED CRYSTALLOGRAPHIC REFEREE.

No syntax errors found. CIF dictionary Interpreting this report

Datablock: 3_a

Bond precision: C-C = 0.0024 A Wavelength=0.71073

Cell:	a=15.7327(11)	b=6.1644(4)	c=25.6935(17)
	alpha=90	beta=95.532(1)	gamma=90
Temperature:	173 K		

	Calculated	Reported
Volume	2480.2(3)	2480.2(3)
Space group	P 21/c	P 21/c
Hall group	-P 2ybc	-P 2ybc
Moiety formula	C32 H31 B O2	?
Sum formula	C32 H31 B O2	C32 H31 B O2
Mr	458.38	458.38
Dx,g cm-3	1.228	1.228
Z	4	4
Mu (mm-1)	0.074	0.074
F000	976.0	976.0
F000'	976.39	
h,k,lmax	20,8,34	20,8,31
Nref	6156	5837
Tmin,Tmax	0.991,0.993	0.683,0.746
Tmin'	0.954	

Correction method= # Reported T Limits: Tmin=0.683 Tmax=0.746
AbsCorr = MULTI-SCAN

Data completeness= 0.948 Theta(max)= 28.290

R(reflections)= 0.0494(4215) wR2(reflections)= 0.1284(5837)

S = 1.029 Npar= 316

The following ALERTS were generated. Each ALERT has the format
test-name_ALERT_alert-type_alert-level.
Click on the hyperlinks for more details of the test.

Alert level C

```
PLAT029_ALERT_3_C _diffrn_measured_fraction_theta_full value Low .      0.974 Why?
PLAT906_ALERT_3_C Large K Value in the Analysis of Variance ......      2.388 Check
PLAT911_ALERT_3_C Missing FCF Refl Between Thmin & STh/L=    0.600        115 Report
```

Alert level G

```
PLAT063_ALERT_4_G Crystal Size Possibly too Large for Beam Size ..       0.64 mm
PLAT883_ALERT_1_G No Info/Value for _atom_sites_solution_primary .     Please Do !
PLAT912_ALERT_4_G Missing # of FCF Reflections Above STh/L=  0.600        143 Note
PLAT941_ALERT_3_G Average HKL Measurement Multiplicity ..........         3.6 Low
PLAT952_ALERT_5_G Calculated (ThMax) and CIF-Reported Lmax Differ           3 Units
PLAT958_ALERT_1_G Calculated (ThMax) and Actual (FCF) Lmax Differ           3 Units
PLAT978_ALERT_2_G Number C-C Bonds with Positive Residual Density.         11 Info
PLAT992_ALERT_5_G Repd & Actual _reflns_number_gt Values Differ by          2 Check
```

0 **ALERT level A** = Most likely a serious problem - resolve or explain
0 **ALERT level B** = A potentially serious problem, consider carefully
3 **ALERT level C** = Check. Ensure it is not caused by an omission or oversight
8 **ALERT level G** = General information/check it is not something unexpected

2 ALERT type 1 CIF construction/syntax error, inconsistent or missing data
1 ALERT type 2 Indicator that the structure model may be wrong or deficient
4 ALERT type 3 Indicator that the structure quality may be low
2 ALERT type 4 Improvement, methodology, query or suggestion
2 ALERT type 5 Informative message, check

It is advisable to attempt to resolve as many as possible of the alerts in all categories. Often the minor alerts point to easily fixed oversights, errors and omissions in your CIF or refinement strategy, so attention to these fine details can be worthwhile. In order to resolve some of the more serious problems it may be necessary to carry out additional measurements or structure refinements. However, the purpose of your study may justify the reported deviations and the more serious of these should normally be commented upon in the discussion or experimental section of a paper or in the "special_details" fields of the CIF. checkCIF was carefully designed to identify outliers and unusual parameters, but every test has its limitations and alerts that are not important in a particular case may appear. Conversely, the absence of alerts does not guarantee there are no aspects of the results needing attention. It is up to the individual to critically assess their own results and, if necessary, seek expert advice.

Publication of your CIF in IUCr journals

A basic structural check has been run on your CIF. These basic checks will be run on all CIFs submitted for publication in IUCr journals (*Acta Crystallographica*, *Journal of Applied Crystallography*, *Journal of Synchrotron Radiation*); however, if you intend to submit to *Acta Crystallographica Section C* or *E* or *IUCrData*, you should make sure that full publication checks are run on the final version of your CIF prior to submission.

Publication of your CIF in other journals

Please refer to the *Notes for Authors* of the relevant journal for any special instructions relating to CIF submission.

PLATON version of 18/12/2021; check.def file version of 18/12/2021

Datablock 3_a - ellipsoid plot

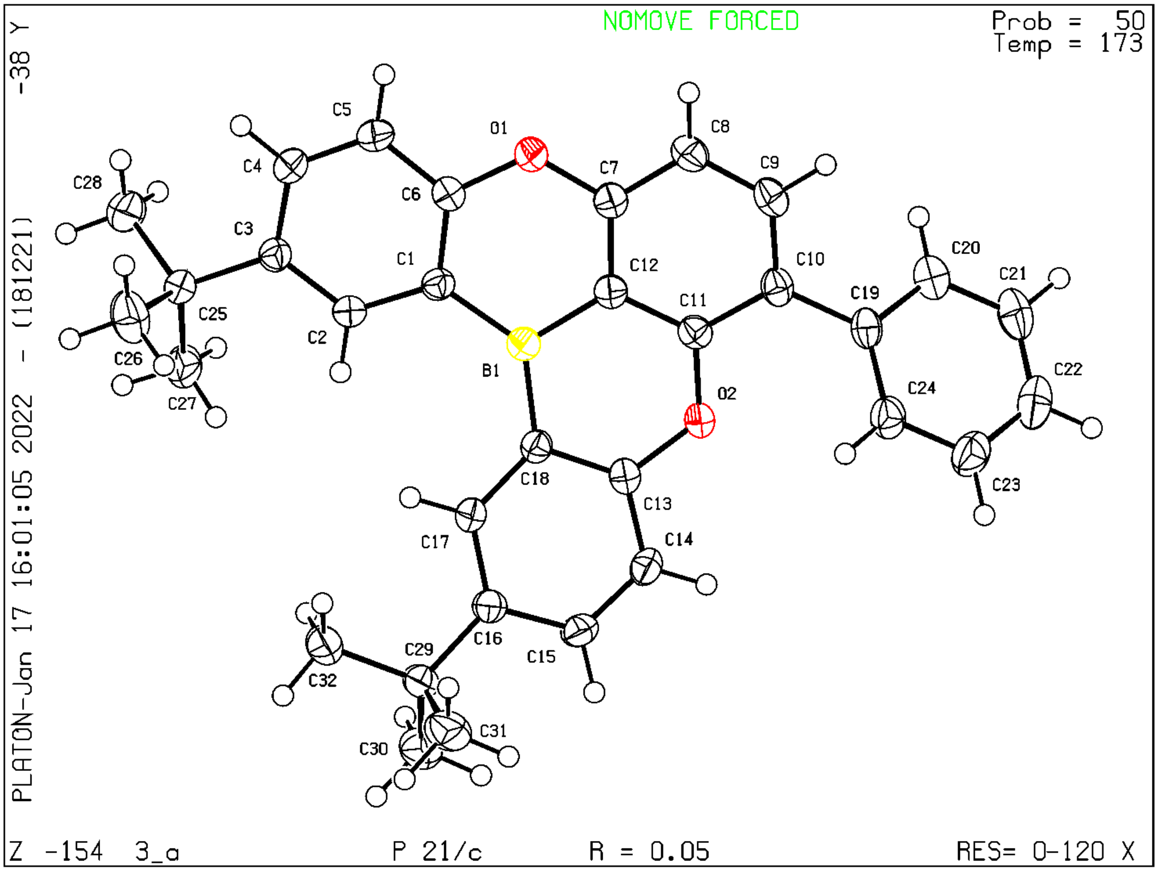

Supplement: Supplementary file 8 — Supplementary Data 5 [file 41467_2023_41440_MOESM8_ESM.pdf]
