# Supplementary material for: High-performance blue OLED using multiresonance thermally activated delayed fluorescence host materials containing silicon atoms
Source: Nat Commun. 2023 Sep 11;14:5589. doi: 10.1038/s41467-023-41440-1 (PMC10495399; doi:10.1038/s41467-023-41440-1)

checkCIF/PLATON report

Structure factors have been supplied for datablock(s) 6_A

THIS REPORT IS FOR GUIDANCE ONLY. IF USED AS PART OF A REVIEW PROCEDURE FOR PUBLICATION, IT SHOULD NOT REPLACE THE EXPERTISE OF AN EXPERIENCED CRYSTALLOGRAPHIC REFEREE.

No syntax errors found. CIF dictionary Interpreting this report

Datablock: 6_A

Bond precision: C-C = 0.0029 A Wavelength=0.71073

Cell:	a=9.6904(2)	b=13.9608(3)	c=14.9313(2)
	alpha=86.878(1)	beta=83.413(1)	gamma=86.479(1)
Temperature:	173 K		

	Calculated	Reported
Volume	2000.58(7)	2000.58(7)
Space group	P -1	P -1
Hall group	-P 1	-P 1
Moiety formula	C50 H45 B O2 Si	?
Sum formula	C50 H45 B O2 Si	C50 H45 B O2 Si
Mr	716.76	716.76
Dx,g cm-3	1.190	1.190
Z	2	2
Mu (mm-1)	0.098	0.098
F000	760.0	760.0
F000’	760.44	
h,k,lmax	12,18,19	12,18,19
Nref	9969	9943
Tmin,Tmax	0.983,0.988	0.714,0.746
Tmin’	0.946	

Correction method= # Reported T Limits: Tmin=0.714 Tmax=0.746
AbsCorr = MULTI-SCAN

Data completeness= 0.997 Theta(max)= 28.343

R(reflections)= 0.0558(7132) wR2(reflections)= 0.1414(9943)

S = 1.023 Npar= 544

The following ALERTS were generated. Each ALERT has the format
test-name_ALERT_alert-type_alert-level.
Click on the hyperlinks for more details of the test.

Alert level C

```
PLAT215_ALERT_3_C Disordered C20A      has ADP max/min Ratio .....         3.7 Note
PLAT220_ALERT_2_C NonSolvent   Resd 1  C   Ueq(max)/Ueq(min) Range         4.0 Ratio
PLAT222_ALERT_3_C NonSolvent Resd 1  H   Uiso(max)/Uiso(min) Range         4.6 Ratio
PLAT242_ALERT_2_C Low    'MainMol' Ueq as Compared to Neighbors of         C19 Check
PLAT242_ALERT_2_C Low    'MainMol' Ueq as Compared to Neighbors of         C29 Check
PLAT906_ALERT_3_C Large K Value in the Analysis of Variance ......       3.311 Check
```

Alert level G

```
PLAT003_ALERT_2_G Number of Uiso or Uij Restrained non-H Atoms ...           4 Report
PLAT154_ALERT_1_G The s.u.'s on the Cell Angles are Equal ..(Note)       0.001 Degree
PLAT186_ALERT_4_G The CIF-Embedded .res File Contains ISOR Records           1 Report
PLAT230_ALERT_2_G Hirshfeld Test Diff for    C19      --C20B     .         9.0 s.u.
PLAT230_ALERT_2_G Hirshfeld Test Diff for    C19      --C21B     .        12.5 s.u.
PLAT230_ALERT_2_G Hirshfeld Test Diff for    C29      --C30B     .         8.8 s.u.
PLAT300_ALERT_4_G Atom Site Occupancy of C20A       Constrained at         0.5 Check
PLAT300_ALERT_4_G Atom Site Occupancy of C20B       Constrained at         0.5 Check
PLAT300_ALERT_4_G Atom Site Occupancy of C21A       Constrained at         0.5 Check
PLAT300_ALERT_4_G Atom Site Occupancy of C21B       Constrained at         0.5 Check
PLAT300_ALERT_4_G Atom Site Occupancy of C22A       Constrained at         0.5 Check
PLAT300_ALERT_4_G Atom Site Occupancy of C22B       Constrained at         0.5 Check
PLAT300_ALERT_4_G Atom Site Occupancy of C30A       Constrained at         0.6 Check
PLAT300_ALERT_4_G Atom Site Occupancy of C31A       Constrained at         0.7 Check
PLAT300_ALERT_4_G Atom Site Occupancy of C32A       Constrained at         0.6 Check
PLAT300_ALERT_4_G Atom Site Occupancy of C30B       Constrained at         0.4 Check
PLAT300_ALERT_4_G Atom Site Occupancy of C31B       Constrained at         0.3 Check
PLAT300_ALERT_4_G Atom Site Occupancy of C32B       Constrained at         0.4 Check
PLAT300_ALERT_4_G Atom Site Occupancy of H20A       Constrained at         0.5 Check
PLAT300_ALERT_4_G Atom Site Occupancy of H20B       Constrained at         0.5 Check
PLAT300_ALERT_4_G Atom Site Occupancy of H20C       Constrained at         0.5 Check
PLAT300_ALERT_4_G Atom Site Occupancy of H20D       Constrained at         0.5 Check
PLAT300_ALERT_4_G Atom Site Occupancy of H20E       Constrained at         0.5 Check
PLAT300_ALERT_4_G Atom Site Occupancy of H20F       Constrained at         0.5 Check
PLAT300_ALERT_4_G Atom Site Occupancy of H21A       Constrained at         0.5 Check
PLAT300_ALERT_4_G Atom Site Occupancy of H21B       Constrained at         0.5 Check
PLAT300_ALERT_4_G Atom Site Occupancy of H21C       Constrained at         0.5 Check
PLAT300_ALERT_4_G Atom Site Occupancy of H21D       Constrained at         0.5 Check
PLAT300_ALERT_4_G Atom Site Occupancy of H21E       Constrained at         0.5 Check
PLAT300_ALERT_4_G Atom Site Occupancy of H21F       Constrained at         0.5 Check
PLAT300_ALERT_4_G Atom Site Occupancy of H22A       Constrained at         0.5 Check
PLAT300_ALERT_4_G Atom Site Occupancy of H22B       Constrained at         0.5 Check
PLAT300_ALERT_4_G Atom Site Occupancy of H22C       Constrained at         0.5 Check
PLAT300_ALERT_4_G Atom Site Occupancy of H22D       Constrained at         0.5 Check
PLAT300_ALERT_4_G Atom Site Occupancy of H22E       Constrained at         0.5 Check
PLAT300_ALERT_4_G Atom Site Occupancy of H22F       Constrained at         0.5 Check
PLAT300_ALERT_4_G Atom Site Occupancy of H30A       Constrained at         0.6 Check
PLAT300_ALERT_4_G Atom Site Occupancy of H30B       Constrained at         0.6 Check
PLAT300_ALERT_4_G Atom Site Occupancy of H30C       Constrained at         0.6 Check
PLAT300_ALERT_4_G Atom Site Occupancy of H31A       Constrained at         0.7 Check
PLAT300_ALERT_4_G Atom Site Occupancy of H31B       Constrained at         0.7 Check
```

```
PLAT300_ALERT_4_G Atom Site Occupancy of H31C       Constrained at        0.7 Check
PLAT300_ALERT_4_G Atom Site Occupancy of H32A       Constrained at        0.6 Check
PLAT300_ALERT_4_G Atom Site Occupancy of H32B       Constrained at        0.6 Check
PLAT300_ALERT_4_G Atom Site Occupancy of H32C       Constrained at        0.6 Check
PLAT300_ALERT_4_G Atom Site Occupancy of H30D       Constrained at        0.4 Check
PLAT300_ALERT_4_G Atom Site Occupancy of H30E       Constrained at        0.4 Check
PLAT300_ALERT_4_G Atom Site Occupancy of H30F       Constrained at        0.4 Check
PLAT300_ALERT_4_G Atom Site Occupancy of H31D       Constrained at        0.3 Check
PLAT300_ALERT_4_G Atom Site Occupancy of H31E       Constrained at        0.3 Check
PLAT300_ALERT_4_G Atom Site Occupancy of H31F       Constrained at        0.3 Check
PLAT300_ALERT_4_G Atom Site Occupancy of H32D       Constrained at        0.4 Check
PLAT300_ALERT_4_G Atom Site Occupancy of H32E       Constrained at        0.4 Check
PLAT300_ALERT_4_G Atom Site Occupancy of H32F       Constrained at        0.4 Check
PLAT301_ALERT_3_G Main Residue  Disorder ..............(Resd  1  )        11% Note
PLAT412_ALERT_2_G Short Intra XH3 .. XHn     H16A     ..H20A     .       2.10 Ang.
                                                        x,y,z  =      1_555 Check
PLAT412_ALERT_2_G Short Intra XH3 .. XHn     H18A     ..H22D     .       2.05 Ang.
                                                        x,y,z  =      1_555 Check
PLAT412_ALERT_2_G Short Intra XH3 .. XHn     H26A     ..H31D     .       2.14 Ang.
                                                        x,y,z  =      1_555 Check
PLAT412_ALERT_2_G Short Intra XH3 .. XHn     H28A     ..H30D     .       2.06 Ang.
                                                        x,y,z  =      1_555 Check
PLAT860_ALERT_3_G Number of Least-Squares Restraints .............         24 Note
PLAT883_ALERT_1_G No Info/Value for _atom_sites_solution_primary .     Please Do !
PLAT912_ALERT_4_G Missing # of FCF Reflections Above STh/L=  0.600         24 Note
PLAT941_ALERT_3_G Average HKL Measurement Multiplicity ...........        3.3 Low
PLAT978_ALERT_2_G Number C-C Bonds with Positive Residual Density.          9 Info
```

---

0 **ALERT level A** = Most likely a serious problem - resolve or explain
0 **ALERT level B** = A potentially serious problem, consider carefully
6 **ALERT level C** = Check. Ensure it is not caused by an omission or oversight
64 **ALERT level G** = General information/check it is not something unexpected

2 ALERT type 1 CIF construction/syntax error, inconsistent or missing data
12 ALERT type 2 Indicator that the structure model may be wrong or deficient
6 ALERT type 3 Indicator that the structure quality may be low
50 ALERT type 4 Improvement, methodology, query or suggestion
0 ALERT type 5 Informative message, check

It is advisable to attempt to resolve as many as possible of the alerts in all categories. Often the minor alerts point to easily fixed oversights, errors and omissions in your CIF or refinement strategy, so attention to these fine details can be worthwhile. In order to resolve some of the more serious problems it may be necessary to carry out additional measurements or structure refinements. However, the purpose of your study may justify the reported deviations and the more serious of these should normally be commented upon in the discussion or experimental section of a paper or in the "special_details" fields of the CIF. checkCIF was carefully designed to identify outliers and unusual parameters, but every test has its limitations and alerts that are not important in a particular case may appear. Conversely, the absence of alerts does not guarantee there are no aspects of the results needing attention. It is up to the individual to critically assess their own results and, if necessary, seek expert advice.

Publication of your CIF in IUCr journals

A basic structural check has been run on your CIF. These basic checks will be run on all CIFs submitted for publication in IUCr journals (*Acta Crystallographica*, *Journal of Applied Crystallography*, *Journal of Synchrotron Radiation*); however, if you intend to submit to *Acta Crystallographica Section C* or *E* or *IUCrData*, you should make sure that full publication checks are run on the final version of your CIF prior to submission.

Publication of your CIF in other journals

Please refer to the *Notes for Authors* of the relevant journal for any special instructions relating to CIF submission.

PLATON version of 20/01/2022; check.def file version of 19/01/2022

Datablock 6_A - ellipsoid plot

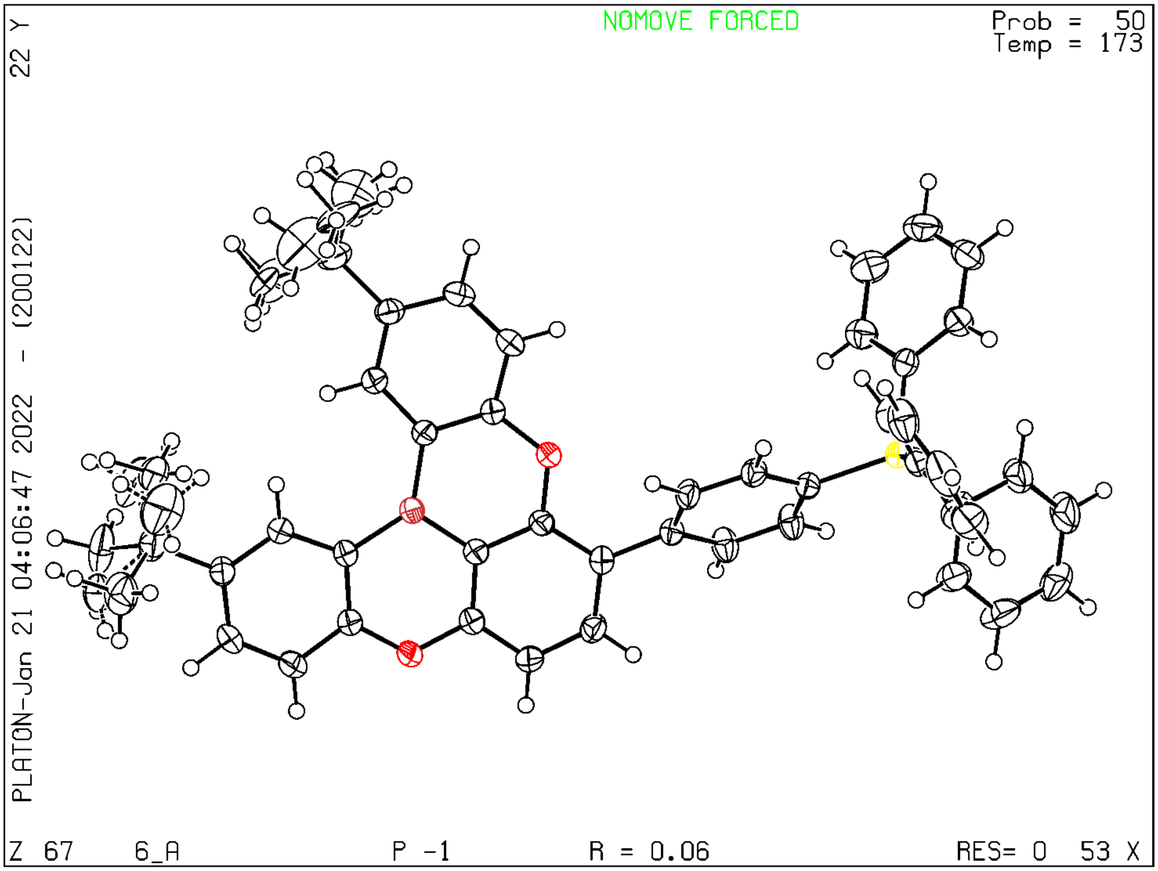

Supplement: Supplementary file 9 — Supplementary Data 6 [file 41467_2023_41440_MOESM9_ESM.pdf]
