# Supplementary material for: High-performance blue OLED using multiresonance thermally activated delayed fluorescence host materials containing silicon atoms
Source: Nat Commun. 2023 Sep 11;14:5589. doi: 10.1038/s41467-023-41440-1 (PMC10495399; doi:10.1038/s41467-023-41440-1)

checkCIF/PLATON report

Structure factors have been supplied for datablock(s) 2_a

THIS REPORT IS FOR GUIDANCE ONLY. IF USED AS PART OF A REVIEW PROCEDURE FOR PUBLICATION, IT SHOULD NOT REPLACE THE EXPERTISE OF AN EXPERIENCED CRYSTALLOGRAPHIC REFEREE.

No syntax errors found. CIF dictionary Interpreting this report

Datablock: 2_a

```
Bond precision:   C-C = 0.0044 A          Wavelength=0.71073

Cell:             a=16.8570(9)     b=13.4848(7)        c=22.8866(12)
                  alpha=90         beta=106.049(3)     gamma=90
Temperature:      173 K

                 Calculated                  Reported
Volume           4999.7(5)                   4999.7(5)
Space group      P 21/n                      P 21/n
Hall group       -P 2yn                      -P 2yn
Moiety formula   C32 H31 B O2                ?
Sum formula      C32 H31 B O2                C32 H31 B O2
Mr               458.38                      458.38
Dx,g cm-3        1.218                       1.218
Z                8                           8
Mu (mm-1)        0.073                       0.073
F000             1952.0                      1952.0
F000'            1952.79
h,k,lmax         21,17,29                    21,17,29
Nref             11553                       11393
Tmin,Tmax        0.978,0.986                 0.690,0.746
Tmin'            0.975

Correction method= # Reported T Limits: Tmin=0.690 Tmax=0.746
AbsCorr = MULTI-SCAN

Data completeness= 0.986           Theta(max)= 27.558

R(reflections)= 0.0827( 8315)                         wR2(reflections)=
                                                      0.2366( 11393)
S = 1.096                  Npar= 658
```

The following ALERTS were generated. Each ALERT has the format
test-name_ALERT_alert-type_alert-level.
Click on the hyperlinks for more details of the test.

Alert level C

```
PLAT215_ALERT_3_C Disordered C53A      has ADP max/min Ratio .....         3.4 Note
PLAT215_ALERT_3_C Disordered C54B      has ADP max/min Ratio .....         3.5 Note
PLAT220_ALERT_2_C NonSolvent   Resd 1  C   Ueq(max)/Ueq(min) Range         5.4 Ratio
PLAT222_ALERT_3_C NonSolvent Resd 1  H   Uiso(max)/Uiso(min) Range         6.0 Ratio
PLAT234_ALERT_4_C Large Hirshfeld Difference C51      --C54B     .        0.16 Ang.
PLAT242_ALERT_2_C Low    'MainMol' Ueq as Compared to Neighbors of         C51 Check
PLAT340_ALERT_3_C Low Bond Precision on  C-C Bonds ..............      0.00437 Ang.
PLAT906_ALERT_3_C Large K Value in the Analysis of Variance ......       6.792 Check
PLAT911_ALERT_3_C Missing FCF Refl Between Thmin & STh/L=    0.600          56 Report
```

Alert level G

```
PLAT003_ALERT_2_G Number of Uiso or Uij Restrained non-H Atoms ...           2 Report
PLAT083_ALERT_2_G SHELXL Second Parameter in WGHT  Unusually Large        7.64 Why ?
PLAT186_ALERT_4_G The CIF-Embedded .res File Contains ISOR Records           2 Report
PLAT230_ALERT_2_G Hirshfeld Test Diff for    C51      --C53B     .        12.3 s.u.
PLAT300_ALERT_4_G Atom Site Occupancy of C52A       Constrained at         0.5 Check
PLAT300_ALERT_4_G Atom Site Occupancy of C52B       Constrained at         0.5 Check
PLAT300_ALERT_4_G Atom Site Occupancy of C53A       Constrained at         0.5 Check
PLAT300_ALERT_4_G Atom Site Occupancy of C53B       Constrained at         0.5 Check
PLAT300_ALERT_4_G Atom Site Occupancy of C54A       Constrained at         0.5 Check
PLAT300_ALERT_4_G Atom Site Occupancy of C54B       Constrained at         0.5 Check
PLAT300_ALERT_4_G Atom Site Occupancy of H52A       Constrained at         0.5 Check
PLAT300_ALERT_4_G Atom Site Occupancy of H52B       Constrained at         0.5 Check
PLAT300_ALERT_4_G Atom Site Occupancy of H52C       Constrained at         0.5 Check
PLAT300_ALERT_4_G Atom Site Occupancy of H52D       Constrained at         0.5 Check
PLAT300_ALERT_4_G Atom Site Occupancy of H52E       Constrained at         0.5 Check
PLAT300_ALERT_4_G Atom Site Occupancy of H52F       Constrained at         0.5 Check
PLAT300_ALERT_4_G Atom Site Occupancy of H53A       Constrained at         0.5 Check
PLAT300_ALERT_4_G Atom Site Occupancy of H53B       Constrained at         0.5 Check
PLAT300_ALERT_4_G Atom Site Occupancy of H53C       Constrained at         0.5 Check
PLAT300_ALERT_4_G Atom Site Occupancy of H53D       Constrained at         0.5 Check
PLAT300_ALERT_4_G Atom Site Occupancy of H53E       Constrained at         0.5 Check
PLAT300_ALERT_4_G Atom Site Occupancy of H53F       Constrained at         0.5 Check
PLAT300_ALERT_4_G Atom Site Occupancy of H54A       Constrained at         0.5 Check
PLAT300_ALERT_4_G Atom Site Occupancy of H54B       Constrained at         0.5 Check
PLAT300_ALERT_4_G Atom Site Occupancy of H54C       Constrained at         0.5 Check
PLAT300_ALERT_4_G Atom Site Occupancy of H54D       Constrained at         0.5 Check
PLAT300_ALERT_4_G Atom Site Occupancy of H54E       Constrained at         0.5 Check
PLAT300_ALERT_4_G Atom Site Occupancy of H54F       Constrained at         0.5 Check
PLAT301_ALERT_3_G Main Residue  Disorder ..............(Resd  1  )          9% Note
PLAT412_ALERT_2_G Short Intra XH3 .. XHn     H48A      ..H52E     .        2.09 Ang.
                                                   x,y,z  =      1_555 Check
PLAT860_ALERT_3_G Number of Least-Squares Restraints ............           12 Note
PLAT883_ALERT_1_G No Info/Value for _atom_sites_solution_primary .     Please Do !
PLAT910_ALERT_3_G Missing # of FCF Reflection(s) Below Theta(Min).           1 Note
PLAT912_ALERT_4_G Missing # of FCF Reflections Above STh/L=  0.600         103 Note
PLAT933_ALERT_2_G Number of HKL-OMIT Records in Embedded .res File           9 Note
PLAT978_ALERT_2_G Number C-C Bonds with Positive Residual Density.           6 Info
PLAT992_ALERT_5_G Repd & Actual _reflns_number_gt Values Differ by           2 Check
```

```
 0 ALERT level A = Most likely a serious problem - resolve or explain
 0 ALERT level B = A potentially serious problem, consider carefully
 9 ALERT level C = Check. Ensure it is not caused by an omission or oversight
37 ALERT level G = General information/check it is not something unexpected

 1 ALERT type 1 CIF construction/syntax error, inconsistent or missing data
 8 ALERT type 2 Indicator that the structure model may be wrong or deficient
 9 ALERT type 3 Indicator that the structure quality may be low
27 ALERT type 4 Improvement, methodology, query or suggestion
 1 ALERT type 5 Informative message, check
```

It is advisable to attempt to resolve as many as possible of the alerts in all categories. Often the minor alerts point to easily fixed oversights, errors and omissions in your CIF or refinement strategy, so attention to these fine details can be worthwhile. In order to resolve some of the more serious problems it may be necessary to carry out additional measurements or structure refinements. However, the purpose of your study may justify the reported deviations and the more serious of these should normally be commented upon in the discussion or experimental section of a paper or in the "special_details" fields of the CIF. checkCIF was carefully designed to identify outliers and unusual parameters, but every test has its limitations and alerts that are not important in a particular case may appear. Conversely, the absence of alerts does not guarantee there are no aspects of the results needing attention. It is up to the individual to critically assess their own results and, if necessary, seek expert advice.

Publication of your CIF in IUCr journals

A basic structural check has been run on your CIF. These basic checks will be run on all CIFs submitted for publication in IUCr journals (*Acta Crystallographica*, *Journal of Applied Crystallography*, *Journal of Synchrotron Radiation*); however, if you intend to submit to *Acta Crystallographica Section C* or *E* or *IUCrData*, you should make sure that full publication checks are run on the final version of your CIF prior to submission.

Publication of your CIF in other journals

Please refer to the *Notes for Authors* of the relevant journal for any special instructions relating to CIF submission.

PLATON version of 18/12/2021; check.def file version of 18/12/2021

Datablock 2_a - ellipsoid plot

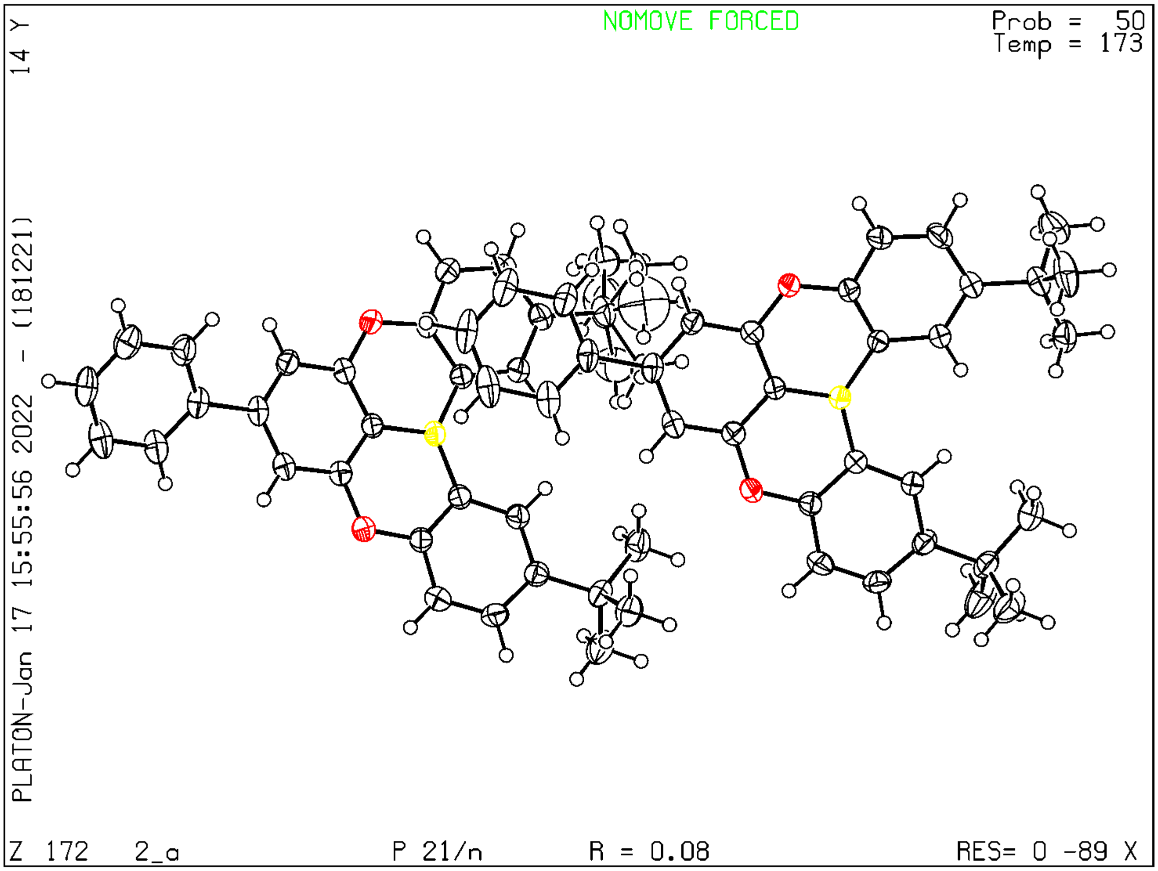

Supplement: Supplementary file 10 — Supplementary Data 7 [file 41467_2023_41440_MOESM10_ESM.pdf]
