# Supplementary material for: High-performance blue OLED using multiresonance thermally activated delayed fluorescence host materials containing silicon atoms
Source: Nat Commun. 2023 Sep 11;14:5589. doi: 10.1038/s41467-023-41440-1 (PMC10495399; doi:10.1038/s41467-023-41440-1)

checkCIF/PLATON report

Structure factors have been supplied for datablock(s) 5_a

THIS REPORT IS FOR GUIDANCE ONLY. IF USED AS PART OF A REVIEW PROCEDURE FOR PUBLICATION, IT SHOULD NOT REPLACE THE EXPERTISE OF AN EXPERIENCED CRYSTALLOGRAPHIC REFEREE.

No syntax errors found. CIF dictionary Interpreting this report

Datablock: 5_a

Bond precision: C-C = 0.0055 A Wavelength=0.71073

Cell:	a=7.4090(2)	b=17.7778(7)	c=15.1799(5)
	alpha=90	beta=98.606(2)	gamma=90
Temperature:	173 K		

	Calculated	Reported
Volume	1976.92(12)	1976.92(12)
Space group	P 21	P 21
Hall group	P 2yb	P 2yb
Moiety formula	C50 H45 B O2 Si	?
Sum formula	C50 H45 B O2 Si	C50 H45 B O2 Si
Mr	716.76	716.76
Dx,g cm-3	1.204	1.204
Z	2	2
Mu (mm-1)	0.100	0.100
F000	760.0	760.0
F000'	760.44	
h,k,lmax	9,23,19	9,23,19
Nref	9056[4676]	6658
Tmin,Tmax	0.988,0.997	0.686,0.746
Tmin'	0.943	

Correction method= # Reported T Limits: Tmin=0.686 Tmax=0.746
AbsCorr = MULTI-SCAN

Data completeness= 1.42/0.74 Theta(max)= 27.462

R(reflections)= 0.0528(4931) wR2(reflections)= 0.1166(6658)

S = 1.024 Npar= 521

The following ALERTS were generated. Each ALERT has the format
test-name_ALERT_alert-type_alert-level.
Click on the hyperlinks for more details of the test.

Alert level B

```
PLAT915_ALERT_3_B No Flack x Check Done: Low Friedel Pair Coverage         45 %
```

Alert level C

```
PLAT220_ALERT_2_C NonSolvent   Resd 1  C   Ueq(max)/Ueq(min) Range        3.5 Ratio
PLAT222_ALERT_3_C NonSolvent Resd 1  H   Uiso(max)/Uiso(min) Range        4.1 Ratio
PLAT242_ALERT_2_C Low    'MainMol' Ueq as Compared to Neighbors of        C23 Check
PLAT340_ALERT_3_C Low Bond Precision on  C-C Bonds ..............      0.0055 Ang.
PLAT790_ALERT_4_C Centre of Gravity not Within Unit Cell: Resd.  #          1 Note
              C50 H45 B O2 Si
```

Alert level G

```
PLAT003_ALERT_2_G Number of Uiso or Uij Restrained non-H Atoms ...          3 Report
PLAT186_ALERT_4_G The CIF-Embedded .res File Contains ISOR Records          1 Report
PLAT230_ALERT_2_G Hirshfeld Test Diff for    C23      --C24B     .        6.0 s.u.
PLAT230_ALERT_2_G Hirshfeld Test Diff for    C23      --C26B     .        7.6 s.u.
PLAT300_ALERT_4_G Atom Site Occupancy of C24A       Constrained at        0.6 Check
PLAT300_ALERT_4_G Atom Site Occupancy of C25A       Constrained at        0.6 Check
PLAT300_ALERT_4_G Atom Site Occupancy of C26A       Constrained at        0.6 Check
PLAT300_ALERT_4_G Atom Site Occupancy of C24B       Constrained at        0.4 Check
PLAT300_ALERT_4_G Atom Site Occupancy of C25B       Constrained at        0.4 Check
PLAT300_ALERT_4_G Atom Site Occupancy of C26B       Constrained at        0.4 Check
PLAT300_ALERT_4_G Atom Site Occupancy of H24A       Constrained at        0.6 Check
PLAT300_ALERT_4_G Atom Site Occupancy of H24B       Constrained at        0.6 Check
PLAT300_ALERT_4_G Atom Site Occupancy of H24C       Constrained at        0.6 Check
PLAT300_ALERT_4_G Atom Site Occupancy of H25A       Constrained at        0.6 Check
PLAT300_ALERT_4_G Atom Site Occupancy of H25B       Constrained at        0.6 Check
PLAT300_ALERT_4_G Atom Site Occupancy of H25C       Constrained at        0.6 Check
PLAT300_ALERT_4_G Atom Site Occupancy of H26A       Constrained at        0.6 Check
PLAT300_ALERT_4_G Atom Site Occupancy of H26B       Constrained at        0.6 Check
PLAT300_ALERT_4_G Atom Site Occupancy of H26C       Constrained at        0.6 Check
PLAT300_ALERT_4_G Atom Site Occupancy of H24D       Constrained at        0.4 Check
PLAT300_ALERT_4_G Atom Site Occupancy of H24E       Constrained at        0.4 Check
PLAT300_ALERT_4_G Atom Site Occupancy of H24F       Constrained at        0.4 Check
PLAT300_ALERT_4_G Atom Site Occupancy of H25D       Constrained at        0.4 Check
PLAT300_ALERT_4_G Atom Site Occupancy of H25E       Constrained at        0.4 Check
PLAT300_ALERT_4_G Atom Site Occupancy of H25F       Constrained at        0.4 Check
PLAT300_ALERT_4_G Atom Site Occupancy of H26D       Constrained at        0.4 Check
PLAT300_ALERT_4_G Atom Site Occupancy of H26E       Constrained at        0.4 Check
PLAT300_ALERT_4_G Atom Site Occupancy of H26F       Constrained at        0.4 Check
PLAT301_ALERT_3_G Main Residue  Disorder ..............(Resd  1  )          6% Note
PLAT412_ALERT_2_G Short Intra XH3 .. XHn     H15A      ..H24D     .       1.98 Ang.
                                                      x,y,z  =      1_555 Check
PLAT412_ALERT_2_G Short Intra XH3 .. XHn     H17A      ..H26D     .       2.04 Ang.
                                                      x,y,z  =      1_555 Check
PLAT860_ALERT_3_G Number of Least-Squares Restraints ............          19 Note
PLAT883_ALERT_1_G No Info/Value for _atom_sites_solution_primary .     Please Do !
PLAT910_ALERT_3_G Missing # of FCF Reflection(s) Below Theta(Min).          1 Note
```

```
PLAT912_ALERT_4_G Missing # of FCF Reflections Above STh/L=  0.600          7 Note
PLAT941_ALERT_3_G Average HKL Measurement Multiplicity ..........         4.1 Low
PLAT965_ALERT_2_G The SHELXL WEIGHT Optimisation has not Converged     Please Check
PLAT978_ALERT_2_G Number C-C Bonds with Positive Residual Density.          5 Info
PLAT992_ALERT_5_G Repd & Actual _reflns_number_gt Values Differ by          3 Check
```

```
   0 ALERT level A = Most likely a serious problem - resolve or explain
   1 ALERT level B = A potentially serious problem, consider carefully
   5 ALERT level C = Check. Ensure it is not caused by an omission or oversight
  39 ALERT level G = General information/check it is not something unexpected

   1 ALERT type 1 CIF construction/syntax error, inconsistent or missing data
   9 ALERT type 2 Indicator that the structure model may be wrong or deficient
   7 ALERT type 3 Indicator that the structure quality may be low
  27 ALERT type 4 Improvement, methodology, query or suggestion
   1 ALERT type 5 Informative message, check
```

It is advisable to attempt to resolve as many as possible of the alerts in all categories. Often the minor alerts point to easily fixed oversights, errors and omissions in your CIF or refinement strategy, so attention to these fine details can be worthwhile. In order to resolve some of the more serious problems it may be necessary to carry out additional measurements or structure refinements. However, the purpose of your study may justify the reported deviations and the more serious of these should normally be commented upon in the discussion or experimental section of a paper or in the "special_details" fields of the CIF. checkCIF was carefully designed to identify outliers and unusual parameters, but every test has its limitations and alerts that are not important in a particular case may appear. Conversely, the absence of alerts does not guarantee there are no aspects of the results needing attention. It is up to the individual to critically assess their own results and, if necessary, seek expert advice.

Publication of your CIF in IUCr journals

A basic structural check has been run on your CIF. These basic checks will be run on all CIFs submitted for publication in IUCr journals (*Acta Crystallographica*, *Journal of Applied Crystallography*, *Journal of Synchrotron Radiation*); however, if you intend to submit to *Acta Crystallographica Section C* or *E* or *IUCrData*, you should make sure that full publication checks are run on the final version of your CIF prior to submission.

Publication of your CIF in other journals

Please refer to the *Notes for Authors* of the relevant journal for any special instructions relating to CIF submission.

PLATON version of 20/01/2022; check.def file version of 19/01/2022

Datablock 5_a - ellipsoid plot

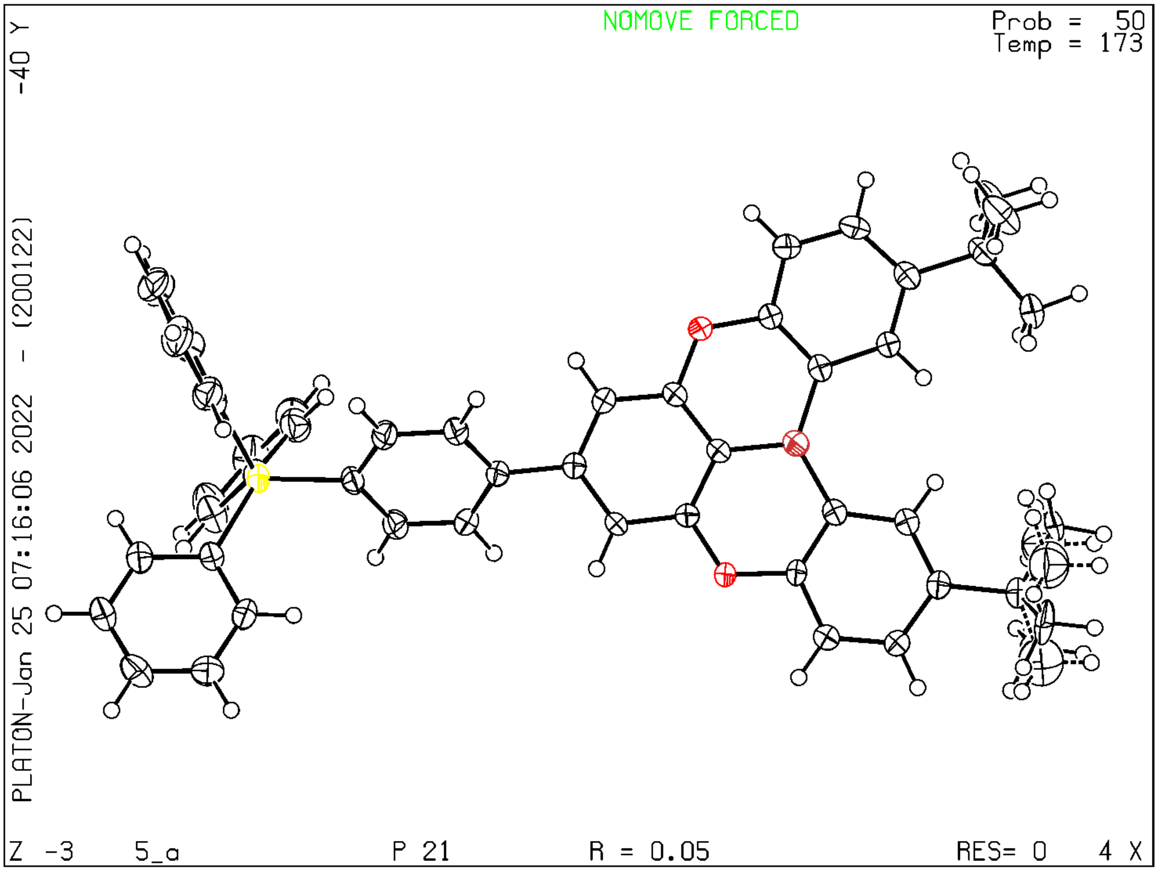

Supplement: Supplementary file 11 — Supplementary Data 8 [file 41467_2023_41440_MOESM11_ESM.pdf]
